# Agreement of MyotonPRO Measurements Across Standing and Prone Positions in Adolescents with Idiopathic Scoliosis

**DOI:** 10.3390/jcm15135051

**Published:** 2026-06-29

**Authors:** Oana-Cristina Rădulescu, Alina-Daniela Totorean, Oana Suciu, Andreea Niță, Liliana Cațan, Alexandra Barzuca, Elena Amăricăi

**Affiliations:** 1 Doctoral School, “Victor Babeș” University of Medicine and Pharmacy, 300041 Timișoara, Romania; oana.radulescu@umft.ro; 2 Department of Rehabilitation, Physical Medicine and Rheumatology, Faculty of Medicine, “Victor Babeș” University of Medicine and Pharmacy, 300041 Timișoara, Romania; oanasuciu78@umft.ro (O.S.); nita.andreea@umft.ro (A.N.); catan.liliana@umft.ro (L.C.); amaricai.elena@umft.ro (E.A.); 3 Transdisciplinary Research Center in Medical Rehabilitation, Balneology and Rheumatology, “Victor Babeș” University of Medicine and Pharmacy, 300041 Timișoara, Romania; 4 Research Center for Assessment of Human Motion, Functionality and Disability, “Victor Babeș” University of Medicine and Pharmacy, 300041 Timișoara, Romania; 5 Physical Education, University Sports Center for Fitness Level Assessment, “Victor Babeș” University of Medicine and Pharmacy, 300041 Timișoara, Romania; barzuca.alexia@umft.ro

**Keywords:** scoliosis, adolescent idiopathic scoliosis (AIS), myotonometry, assessment in standing and prone position, cross-positional agreement

## Abstract

**Background**: Adolescent idiopathic scoliosis (AIS) is associated with alterations in muscle tone, stiffness, and viscoelastic properties that may affect musculoskeletal function and rehabilitation outcomes. Myotonometry offers a non-invasive means of quantifying these properties, but its agreement across testing positions in scoliotic populations remains insufficiently characterized. This study aimed to evaluate the agreement across positions of the MyotonPRO device for assessing superficial back muscle properties in adolescents with mild-to-moderate S-shaped AIS across standing and prone positions, and to examine positional and side-to-side differences. **Methods**: Nineteen adolescents (18 female, 1 male; mean age 15.3 ± 1.8 years) with mild-to-moderate dextroconvex thoracic and sinistroconvex lumbar idiopathic scoliosis were assessed bilaterally at the middle trapezius, lower trapezius, latissimus dorsi, and lumbar erector spinae. Muscle tone, stiffness, elasticity (logarithmic decrement), stress relaxation time, and creep were recorded by a single examiner in both standing and prone positions. Agreement across positions was quantified using intraclass correlation coefficients (ICCs), and between-position differences were tested with paired *t*-tests. **Results**: Agreement across positions was poor for most muscle and parameter combinations. The decrement of the lumbar erector spinae showed the highest agreement bilaterally (ICCs 0.829–0.844, good), and the left middle trapezius showed moderate agreement for tone, stiffness, stress relaxation time and creep (ICCs 0.567–0.649); all other muscle and parameter combinations showed predominantly poor agreement (ICCs < 0.50). Between-position differences were muscle- and side-specific: the middle trapezius differed significantly in all five parameters bilaterally (with higher tone and stiffness in standing), whereas both lumbar erectors showed no significant positional differences. The left lower trapezius and both latissimus dorsi showed lower tone in standing, in contrast to the middle trapezius pattern. **Conclusions**: Across standing and prone positions, MyotonPRO measurements showed predominantly poor absolute agreement for the superficial back muscles examined in adolescents with S-shaped AIS; only the decrement of the lumbar erector spinae reached good agreement.

## 1. Introduction

Adolescent idiopathic scoliosis (AIS) represents the most common form of scoliosis in the pediatric population and is characterized by a three-dimensional spinal deformity arising during adolescence, typically between 10 and 18 years of age, with no identifiable etiology [1]. Beyond physical deformity, AIS may be accompanied by psychological distress, pain, and, in severe cases, impaired pulmonary and cardiac function. Consequently, management strategies for AIS focus not only on preventing curve progression but also on reducing its overall impact on patients’ health and quality of life [1]. In addition, these patients experience a significantly higher incidence of psychological disorders compared with healthy peers, which is often related to their altered physical appearance [2]. Management of AIS includes surgical treatment for severe deformities (Cobb angle > 45°) and conservative approaches for mild (10–25°) and moderate (25–45°) curves. As outlined in the 2016 SOSORT guidelines, conservative treatment goals encompass both morphological objectives, such as aesthetic improvement, and functional objectives, including prevention of curve progression and pain reduction, all of which are integral to quality of life [3].

During sports activities, scoliosis may increase the risk of lower-extremity joint injuries, particularly involving the knees and ankles, due to biomechanical abnormalities that result in uncoordinated and atypical joint movements [4,5]. In scoliosis, alterations occur in muscle tone, elasticity, stiffness, and relaxation, and the paravertebral muscles exhibit distinct properties on the convex versus concave sides of the curve [6]. Muscle tone, stiffness, and viscoelasticity are key properties that influence movement, musculoskeletal (MSK) health, and rehabilitation outcomes [7]. The MyotonPRO device has demonstrated strong reliability across various muscle groups, supporting its clinical and research application [7]. Despite growing interest in the influence of biological and lifestyle factors—such as sex, age, body mass index (BMI), and physical activity—on muscle stiffness, the evidence remains inconclusive. Some studies report higher stiffness in males, age-related changes, or associations with BMI, whereas others find weak or no significant relationships [7].

In clinical practice, myotonometry assessments may be performed in different body positions, most commonly in standing and prone postures, depending on the clinical setting and patient condition. However, the choice of position is not standardized, which may introduce variability in measured muscle mechanical properties. Standing measurements are generally considered to reflect functional, weight-bearing conditions where postural control and neuromuscular activation influence tissue behavior, whereas prone measurements are assumed to better represent passive muscle and soft tissue mechanical properties with minimal postural demand. This distinction is clinically relevant, as posture-dependent differences may affect the interpretation of muscle tone and stiffness and consequently influence clinical decision-making and treatment planning. Furthermore, in rehabilitation contexts, monitoring changes over time requires consistent and meaningful measurement conditions; thus, understanding the extent of variability between positions is essential to ensure accurate tracking of patient progress and treatment effects. Recent systematic reviews confirm the high intra- and inter-rater reliability of myotonometry across diverse populations, showing excellent agreement for parameters like frequency and stiffness [8], including neck and back muscles [9]. Individual and test–retest investigations further demonstrate strong temporal stability, with intraclass correlation coefficient (ICC) values consistently ranging from 0.75 to 0.97 when standardized protocols are applied [10,11].

Despite these promising results, methodological heterogeneity and variability in measurement protocols remain limitations, emphasizing the need for further standardization to improve the clinical applicability and comparability of Myoton-based assessments.

The aim of our study was to evaluate the agreement (cross-positional consistency) of MyotonPRO for assessing biomechanical and viscoelastic properties of superficial back muscles in standing and prone positions in children and adolescents with idiopathic scoliosis. Another objective was to compare the changes in these parameters between standing and prone position for both the right and left sides of scoliosis curves.

## 2. Materials and Methods

This study involves patients with mild to moderate idiopathic dextroconvex thoracic scoliosis and sinistroconvex lumbar scoliosis. Before participation, patients received verbal and written information about the study. The subjects were recruited from the Department of Pediatric Surgery and the Pediatric Rehabilitation Unit of the Louis Țurcanu Children’s Hospital in Timișoara, Romania. Participation was voluntary, and written informed consent was obtained from the parents of all participants. The study was conducted in accordance with the Declaration of Helsinki and received approval from the Institutional Ethics Committee of the Louis Țurcanu Children’s Emergency Hospital (no. 173/2024), as well as the Ethics Committee of the Victor Babeș University of Medicine and Pharmacy in Timișoara (no. 52; approval date: 4 November 2024).

### 2.1. Participants

Nineteen adolescents participated in the study, comprising 18 females and 1 male, which resulted in a notably unbalanced gender distribution. Participants’ ages ranged from 12 to 19 years (M = 15.3, SD = 1.8). Mean height was 162.5 ± 6.8 cm (range: 150–173 cm), mean weight was 48.5 ± 6.9 kg (range: 38–62 kg), and mean body mass index (BMI) was 17.6 ± 1.8 kg/m^2^ (range: 15.37–21.87 kg/m^2^) (Table 1). All participants were recruited according to inclusion criteria, such as being adolescents diagnosed with idiopathic scoliosis and free of other musculoskeletal disorders. The marked gender imbalance in the sample is acknowledged as a limitation that may constrain the generalizability of the results.

#### 2.1.1. Inclusion Criteria

The study population consists of children and adolescents aged between 10 and 19 years who are diagnosed with mild to moderate idiopathic dextroconvex thoracic and sinistroconvex lumbar scoliosis. Participants present both clinical and radiological evidence of the condition and provide voluntary consent to participate in the study. Curve severity was restricted to the mild-to-moderate range, corresponding to a Cobb angle of 10–45° according to the definitions used in this study.

#### 2.1.2. Exclusion Criteria

Patients were excluded if they had undergone orthopedic surgery of the lower limbs within the previous three months, presented disorders of static balance or gait, or had neurological conditions affecting balance, orthostasis, or gait. Additionally, individuals with impaired ability to cooperate or with cognitive impairments were excluded from the study.

### 2.2. Measurements

Patient measurements were performed using the MyotonPRO. The subject was assessed in two positions: standing and prone, with bilateral comparisons between the left and right sides. Participants were instructed to remain as relaxed as possible during the measurements. The ambient environment was maintained at a constant and appropriate temperature, and subjects were not exposed to internal or external disturbing factors within the measurement setting. In the standing position, the patient stood upright with the arms alongside the body in a relaxed posture. The spinal curvature was used as a reference point; the contour of the spinous processes was marked with a red point, and measurement points were marked in black approximately 2 cm lateral to the spinous processes. The muscles evaluated included the middle and lower trapezius, latissimus dorsi, and lumbar erector spinae. For each participant, the first assessment was conducted in the standing position, followed by a 10 min rest period. Measurements in the prone decubitus position were then performed on an examination couch, the same couch being used for all participants. In this position, the patient lay prone with the arms alongside the body and the head in a neutral position to ensure maximal relaxation. All measurements were conducted by the same examiner, using the same device, under identical testing conditions. Although measurements were performed by a single examiner (intra-rater condition), the ICC analysis in this study reflects agreement between two distinct biomechanical testing conditions (standing vs. prone), rather than traditional same-position repeatability. Myotonometric assessment performed in the standing position should be interpreted with caution, as the obtained parameters represent a composite outcome influenced by both passive and active components of muscle function. While devices such as the MyotonPRO have demonstrated good to excellent reliability across different muscle groups and populations [8,10], the measurement conditions substantially influence the results. In contrast to the prone position, the standing posture requires continuous postural control, leading to persistent low-level activation of trunk musculature, which may increase apparent muscle tone and stiffness [11]. Additionally, gravitational loading and postural demands further modify tissue mechanical behavior, limiting the ability to isolate intrinsic viscoelastic properties [12]. In individuals with scoliosis, these effects are exacerbated by asymmetrical load distribution and altered neuromuscular strategies, which may confound side-to-side comparisons. Moreover, factors such as probe placement variability, postural sway, and respiratory-induced fluctuations contribute to measurement variability and may reduce reproducibility under functional conditions. Therefore, myotonometric data obtained in the standing position should be considered reflective of functional muscle behavior under load rather than purely passive mechanical properties, and comparisons with unloaded conditions should be made with caution [8,13].

### 2.3. Myotonometry

Myotonometry is a non-invasive method used to evaluate the mechanical properties of skeletal muscles, including muscle tone, stiffness, and elasticity. This assessment is performed using the MyotonPRO device, a portable instrument that applies a controlled mechanical impulse to the muscle and records its response. The device simultaneously calculates the following parameters:

- Muscle tone (tension state): Natural oscillation frequency (Hz), reflecting the intrinsic tension of the muscle at rest;
- Biomechanical properties: Dynamic stiffness (N/m) and logarithmic decrement, the latter indicating muscle elasticity and the dissipation of oscillatory energy;
- Viscoelastic properties: Mechanical stress relaxation time (ms) and the ratio between relaxation and deformation time, which characterizes creep behavior (Deborah number) [14].

It applied five mechanical pulses at one-second intervals (0.18 N), followed by an additional 15 ms pulse (0.40 N) that compressed the subcutaneous tissue at the center of the muscles. These pulses transmitted data to the accelerometer probe attached to the MyotonPRO, which calculated the biomechanical and viscoelastic properties [14].

Patients were asked to lie down with their paravertebral muscles completely relaxed, arms at their sides, and head held in a neutral position with the aid of support. The contours of the spinous processes were traced with a marker pen. Three reference points were identified: at the apex of the curve, at the top and bottom of the main curves (thoracic and lumbar), and 2 cm lateral to the spinous process. Measurements were taken in two positions—standing and prone—at the level of the left and right middle trapezius, left and right lower trapezius, left and right latissimus dorsi, and left and right lumbar erector muscles.

The myotonometric parameters of the paravertebral muscles on the convex side were compared with those on the concave side, and the paravertebral muscles on both the convex and concave sides were analyzed across the three assessments.

All myotonometric assessments were performed using standardized anatomical landmarks. Trapezius measurements were conducted at predefined vertebral levels (C7–T12 region) using consistent reference points to ensure reproducibility across participants. The middle trapezius was assessed at the midpoint between the spinous process of T3–T4 and the medial border of the scapular spine, while the lower trapezius was evaluated approximately 2 cm lateral to the spinous processes at the T7–T12 level. Latissimus dorsi measurements were standardized relative to the inferior angle of the scapula and the posterior axillary line. For each site, three consecutive measurements were obtained, and the mean value was used for statistical analysis. The coefficient of variation provided by the device was monitored to ensure measurement reliability; if variability exceeded the manufacturer’s recommended threshold, additional measurements were performed. To minimize the influence of respiratory activity, all measurements were taken at the end of a normal expiration while participants remained relaxed in each testing position.

Myotonometric parameters were measured bilaterally at the concave and convex sides of the major thoracic curve. Because the cohort presented an S-shaped pattern (dextroconvex thoracic and sinistroconvex lumbar curves), the convex and concave sides differed by spinal region: for the thoracic curve the right side was convex and the left side concave, whereas for the lumbar curve the left side was convex and the right side concave. Accordingly, the middle trapezius, lower trapezius and latissimus dorsi, which relate to the thoracic region, were on the convex side when measured on the right, while the lumbar erector spinae was on the convex side when measured on the left. For clarity, the results are reported using right and left side labels, with the corresponding convex or concave designation noted where relevant. The middle trapezius, lower trapezius, and latissimus dorsi muscles were selected because they are superficial muscles that can be reliably assessed using myotonometry and have previously been investigated in adolescents with idiopathic scoliosis. Gökalp et al. evaluated the viscoelastic properties of the trapezius and latissimus dorsi muscles in adolescents with Lenke type 1 thoracic scoliosis and reported alterations in muscle stiffness and elasticity compared with healthy controls. Furthermore, these muscles contribute to postural control and scapulothoracic stability, functions that may be affected by thoracic spinal deformity [15].

Deep paraspinal muscles such as the multifidus and rotatores were not examined because their anatomical depth limits the validity and reliability of surface myotonometric assessment. Studies investigating these muscles generally employ imaging techniques such as ultrasound, MRI, or elastography [16].

### 2.4. Statistical Analysis

The data analysis was carried out using GraphPad Prism (version 11.0.0) and IBM SPSS Statistics 31 (IBM, Armonk, NY, USA). Intraclass correlation coefficients (ICCs) together with 95% confidence intervals (CI) were used to assess the agreement between the standing and prone positions. The ICCs were estimated using a two-way mixed-effects model with the absolute-agreement definition and the single-measures unit, denoted ICC (A,1) in the McGraw and Wong convention. The two-way mixed-effects formulation was used because the two positions (standing and prone) constituted the only conditions of interest rather than a random sample drawn from a larger population of positions, and the absolute-agreement definition was chosen because the aim was to determine whether the values obtained in the two positions were interchangeable rather than only consistent in rank order. Because the point estimate for absolute agreement is numerically identical under the random- and mixed-effects formulations, this coefficient is equal in value to the two-way random-effects, absolute-agreement, single-measures coefficient denoted ICC(2,1) in the Shrout and Fleiss notation. The absolute-agreement definition was preferred over the consistency definition because systematic differences between positions, which the consistency definition disregards, are directly relevant to the interpretation of myotonometric measurements. The test agreement across positions measured by the ICC was considered excellent (0.90–1.00), good (0.70–0.89), moderate (0.50–0.69) and poor (<0.50) [17].

Descriptive statistics were computed for all variables (mean and standard deviation). Before statistical applications, the normal distribution of values in this study was verified by the D’Agostino-Pearson normality test. The intragroup data (myotonometric parameters in the two testing conditions) were compared with the paired *t*-test. A *p*-value of less than 0.05 was considered statistically significant. The magnitude of the differences between positions was quantified using the standardized effect size for paired samples (Cohen’s dz), interpreted as small (0.20), medium (0.50) or large (0.80). Because 40 muscle–parameter comparisons were performed, the *p*-values were additionally adjusted for multiple testing using the Benjamini–Hochberg false discovery rate (FDR) procedure, and significance was confirmed at an FDR-adjusted threshold of 0.05. In addition, for each muscle and parameter the agreement between positions was characterised by the mean standing/prone difference with its 95% confidence interval, the 95% Bland–Altman limits of agreement (mean difference ± 1.96 SD of the differences), the standard error of measurement (SEM, computed as the standard deviation of the differences divided by √2) and the minimal detectable change at the 95% confidence level (MDC95 = 1.96 × √2 × SEM).

## 3. Results

**Figure 1:**
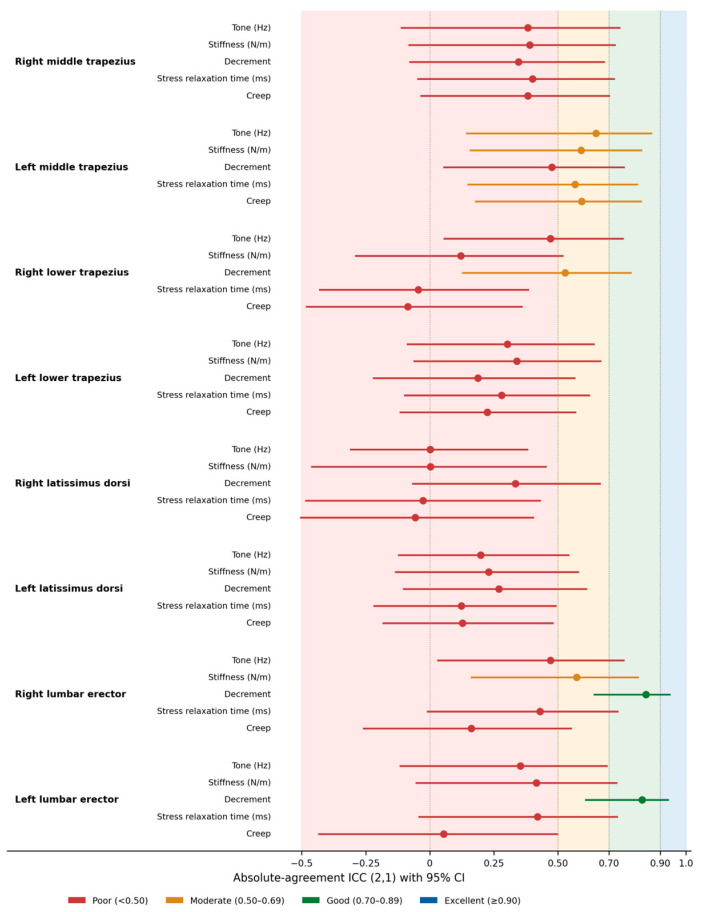
Forest plot of the cross-positional agreement (absolute-agreement, single-measures intraclass correlation coefficient, ICC(A,1), numerically equivalent to the two-way random-effects ICC(2,1)) between the standing and prone positions, with 95% confidence intervals, computed from the raw data for all eight muscle sites and five myotonometric parameters. Shaded bands denote the interpretation thresholds (poor < 0.50, moderate 0.50–0.69, good 0.70–0.89, excellent ≥ 0.90); negative coefficients indicate agreement no greater than that expected by chance.

The agreement between the standing and prone positions, expressed as absolute-agreement single-measures intraclass correlation coefficients, was poor for most muscle and parameter combinations (Table 2, Figure 1). For the right middle trapezius, all five parameters showed poor agreement (ICCs 0.347–0.401). For the left middle trapezius, the tone, stiffness, stress relaxation time and creep showed moderate agreement (ICCs 0.567–0.649), whereas the decrement showed poor agreement (ICC = 0.477). For the right lower trapezius, only the decrement reached moderate agreement (ICC = 0.528), while the remaining parameters showed poor agreement. For the left lower trapezius, and for both the right and left latissimus dorsi, all parameters showed poor agreement (ICCs ≤ 0.34). For the lumbar erector spinae, the decrement showed good agreement bilaterally (ICCs 0.844 and 0.829), the right stiffness showed moderate agreement (ICC = 0.574), and all remaining parameters showed poor agreement. Overall, only the decrement of the lumbar erector spinae reached an agreement level approaching that generally required for clinical use, indicating that for most of the superficial back muscles and parameters examined the standing and prone measurements were not interchangeable.

**Figure 2:**
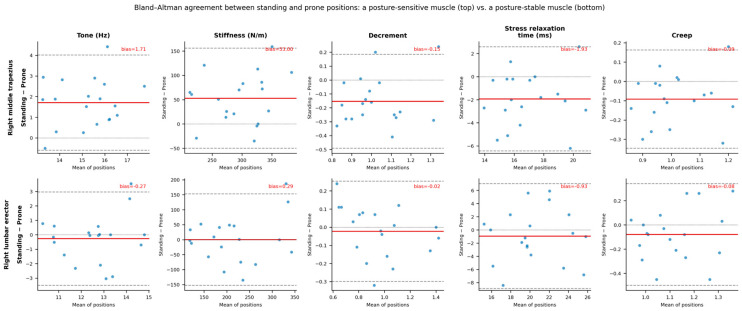
Bland–Altman plots of the agreement between the standing and prone positions for a posture-sensitive muscle (right middle trapezius, (**top row**)) and a posture-stable muscle (right lumbar erector, (**bottom row**)) across the five myotonometric parameters. The solid line denotes the mean difference (bias) and the dashed lines the 95% limits of agreement.

The comparisons of myotonometric parameters between the two testing positions are presented in Table 3 and illustrated for representative muscles in Figure 2. For both the right and left middle trapezius there were statistically significant differences for all five assessed parameters. The tone and stiffness were higher while tested in the standing position. The decrement was lower during the standing testing (higher elasticity in standing position compared to prone one). Both the viscoelastic properties (stress relaxation time and creep) had decreased values when tested during standing position.

For the left lower trapezius there were statistically significant differences for tone and stiffness (lower values in standing position), stress relaxation time and creep (higher values in standing position). When comparing the standing and prone testing positions, for the right lower trapezius (the convex side of the thoracic scoliosis) there were no statistically significant differences for any of the five assessed parameters.

For both the right and left latissimus dorsi there were statistically significant differences for tone (decreased values during standing) and decrement (increased values during standing, thus higher elasticity). For the left latissimus dorsi (the convex side of the lumbar scoliosis) the stiffness was significantly lower during the standing position. In contrast, the stress relaxation time and creep were significantly higher during the standing position.

When comparing the standing and prone testing positions, for both the right and left lumbar erector there were no statistically significant differences for any of the five assessed parameters.

To quantify the practical agreement between the two positions, the mean standing − prone differences with their 95% confidence intervals, the 95% Bland–Altman limits of agreement, the standard error of measurement (SEM) and the minimal detectable change (MDC95) were computed for every muscle and parameter (Table 4); representative Bland–Altman plots are shown in Figure 2. For most muscle–parameter combinations the limits of agreement were wide relative to the measured values, confirming that standing and prone measurements frequently differed by amounts that are clinically relevant. The narrowest relative limits of agreement, together with the smallest SEM and MDC95, were observed for the decrement of the lumbar erector spinae, consistent with its good intraclass agreement, whereas the lower trapezius, the latissimus dorsi and the stiffness parameter showed the widest limits of agreement and the largest measurement error. Systematic biases were also evident, most notably a higher tone and stiffness in standing for the middle trapezius (for example, a mean difference of 1.71 Hz for the right middle trapezius tone), in line with the paired comparisons reported above.

## 4. Discussion

As a three-dimensional spinal deformity, scoliosis interacts dynamically with bipedal posture and gravitational loading, which may be accompanied by neuromuscular adaptations and imbalances in postural muscle groups. Although the structural deformity exists independently, its functional consequences are most pronounced in the standing position, which directly reflects the weight-bearing conditions of the musculoskeletal system [18]. Under these standing conditions, gravitational forces accelerate curvature progression and amplify muscle tone asymmetry, characteristically driven by the continuous activation of the spinal erector musculature [19]. Conversely, prone myotonometric evaluation minimizes gravitational loading and involuntary postural firing, providing a reproducible environment to assess intrinsic viscoelastic properties such as baseline tone, stiffness, and elasticity. Integrating both standing and prone assessments therefore provides a comprehensive approach that effectively differentiates posture-dependent functional adaptations from baseline structural muscle characteristics [20].

The selection of S-shaped scoliosis was based on biomechanical and clinical considerations, as the presence of opposing curves provides complex, multi-regional muscular environments (concave vs. convex, thoracic vs. lumbar) within the same subject. Compared to the uniform and relatively limited imbalance patterns characteristic of simple C-shaped scoliosis, this configuration enhances the sensitivity of detecting intra-individual variations. Consequently, utilizing an S-shaped pattern improves the interpretability and clinical relevance of myotonometric evaluations by allowing direct regional comparisons of muscle mechanical properties. However, given the exploratory nature and the limited sample size of this study, these findings should be considered preliminary. While this S-shaped model provides valuable insights into intra-individual muscular adaptations, further large-scale clinical investigations are required to firmly establish the broader clinical relevance and generalizability of these myotonometric evaluations.

Our study indicates that body position has a clear, muscle-specific influence on myotonometric outcomes in adolescents with mild-to-moderate S-shaped idiopathic scoliosis. While both lumbar erectors remained stable between standing and prone positions, the middle trapezius was the most posture-sensitive muscle, showing significant differences across all five parameters. This impact of posture on muscle mechanical behavior is well-supported by literature. Prior studies reported that the biomechanical properties of the lumbar erector spinae vary based on body position, spinal level, gender, and activation state [21], and that lumbar erector spinae stiffness increases from prone to sitting in patients with chronic low back pain [22]. Consequently, these findings confirm that testing posture modifies myotonometric measurements in a clinically meaningful way, with the response pattern depending strictly on the examined muscle and its biomechanical demands [21,22].

An important finding of the present study is that the effect of body position was not homogeneous across the posterior trunk muscles. In the middle trapezius, standing was associated with higher tone and stiffness, together with lower decrement, relaxation time, and creep, indicating a mechanically more loaded state with greater elasticity during upright postural control. By contrast, the left lower trapezius and both latissimus dorsi exhibited an opposite pattern for several parameters, whereas the right lower trapezius and both lumbar erectors did not show significant positional differences. This variability may reflect the complex three-dimensional compensatory mechanisms of S-shaped scoliosis, in which different muscle groups may assume distinct stabilizing functions according to curve side and anatomical level [21,22].

Previous myotonometric studies in adolescent idiopathic scoliosis (AIS) mainly examined paravertebral muscles close to the main curve, reporting more pronounced side-related asymmetry than observed in our study. For instance, Pan et al. and Liu et al. evaluated patients with severe scoliosis (mean Cobb angle of ~66°) and found significantly higher tone and stiffness on the concave side, alongside longer relaxation times on the convex side, concluding that paraspinal asymmetry correlates with curve severity [16,19]. By contrast, our cohort comprised adolescents with mild-to-moderate S-shaped scoliosis and focused on more superficial posterior trunk muscles (middle/lower trapezius, latissimus dorsi, and lumbar erectors) across two testing positions. This functional design explains the absence of a uniform concave-versus-convex pattern in our results, yielding instead a muscle-specific and posture-dependent response. This interpretation aligns with Chan et al., who highlighted that paraspinal muscle abnormalities in conservatively treated AIS are heterogeneous and highly influenced by anatomical level, curve pattern, and disease severity [23]. Furthermore, Oliva-Pascual-Vaca et al. reported no significant concave-versus-convex differences in milder, single-curve deformities [24]. Thus, our less uniform findings do not contradict previous literature, but rather reflect differences in curve severity, morphology, muscle selection, and testing design [16,19,22,23,24].

Unlike previous studies that primarily focused on deep paraspinal muscles adjacent to the curve apex, the present study investigated predominantly superficial posterior trunk muscles, including the middle trapezius, lower trapezius, latissimus dorsi, and lumbar erector spinae. Recent electromyographic studies have demonstrated that these muscle groups are commonly assessed using surface EMG in both static and dynamic tasks in adolescent idiopathic scoliosis (AIS), showing consistent evidence of asymmetrical activation patterns [25].

These muscles differ functionally from local spinal stabilizers such as the multifidus, which are primarily responsible for segmental spinal control and intervertebral stability [26]. In contrast, superficial trunk muscles contribute predominantly to global postural control, trunk movement, and scapular stabilization, with variable and task-dependent activation patterns reported for latissimus dorsi and erector spinae [27].

Therefore, observed asymmetries in these muscles should be interpreted as functional adaptations of the superficial postural musculature to asymmetric loading conditions associated with scoliosis, rather than direct evidence of alterations in the deep spinal stabilizing system [14]. This interpretation is consistent with recent evidence suggesting that neuromuscular imbalance in AIS reflects both compensatory postural control mechanisms and altered motor strategies rather than isolated dysfunction of deep stabilizers.

Body position is known to influence MyotonPRO measurements, particularly in the lumbar erector spinae [21,22,28]. In the present cohort, however, absolute agreement between the standing and prone positions was poor for most muscles and parameters, the main exception being the decrement of the lumbar erector spinae, which showed good agreement bilaterally (ICC = 0.844 and 0.829). It is important to emphasise that the ICC quantifies the statistical agreement between the two positions and not a physiological property of the muscle itself: a low ICC indicates that the standing and prone values are not interchangeable, but it does not, on its own, demonstrate that a given muscle is biologically more responsive to posture. The direct evidence for genuine positional effects in this study comes from the systematic between-position differences identified in the paired comparisons (Table 3), which were most pronounced for the middle trapezius and minimal for the lumbar erectors. The good absolute agreement of the lumbar erector decrement is therefore consistent with the small and stable positional differences observed at this site, whereas the poor agreement of the more mobile scapulothoracic muscles is consistent with their larger and more variable positional shifts. Taken together, these findings indicate that, for most of the superficial back muscles examined, standing and prone myotonometric values cannot be used interchangeably, and that any interpretation of the underlying muscle behaviour should rely on the measured between-position differences rather than on the magnitude of the agreement coefficient alone.

MyotonPRO performance varies by population, anatomical region, and the specific parameter assessed. In our study, the lumbar erectors exhibited the most reproducible profile under standardized conditions, whereas the lower trapezius and latissimus dorsi were less consistent. This agrees with evidence showing higher reliability for well-defined muscles and primary parameters like tone and stiffness, compared to the greater non-uniformity often seen in viscoelastic indices. Specifically, Lettner et al. demonstrated promising reliability across various muscles, while Koterba and Saulicz noted that back region reliability is generally good but variable [8,9]. The lower consistency of the lower trapezius and latissimus dorsi likely stems from their broad anatomy, oblique fiber orientation, and close interaction with scapulothoracic and thoracolumbar movements. In scoliosis, these features amplify sensitivity to minor variations in probe placement, baseline muscle states, and compensatory activation. Furthermore, the lower stability of creep reflects its complex viscoelastic nature, making it highly susceptible to subtle variations in preload and tissue composition compared to tone or stiffness.

Positional comparisons can be meaningfully discussed in relation to previous studies comparing prone, sitting, standing, or flexed postures, because all of these designs address the effect of body configuration on muscle mechanical behavior. In our study the ICC values reflect consistency across two different testing positions rather than classical same-position intra-rater reliability. Therefore, ICC values in this study should be interpreted as indicators of cross-postural agreement under identical examiner conditions rather than true test–retest reliability within a single standardized position. Our findings suggest that the middle trapezius is highly posture-sensitive, the lumbar erectors are comparatively posture-stable in mild-to-moderate S-shaped AIS, and some viscoelastic variables–especially creep–may be more vulnerable to between-position variation than tone or stiffness [8,9,21,22].

These findings should be interpreted within a framework that clearly separates statistical significance, measurement reliability, and clinical usefulness, as these concepts are not equivalent. A statistically significant difference between positions (Table 3) demonstrates only that a systematic positional effect exists, not that either position yields interchangeable measurements. Similarly, an ICC that differs significantly from zero does not by itself establish that agreement is adequate for practical use. Moderate coefficients (0.50–0.69) reflect limited interchangeability, and most clinical applications, particularly those informing decisions in individual patients, require ICC values above 0.75, with values above 0.90 recommended when measurements guide important clinical judgments [17]. Accordingly, the moderate cross-positional agreement observed for several muscles and parameters in this exploratory study should be regarded as preliminary evidence of consistency rather than confirmation that standing and prone measurements are clinically interchangeable.

The standard error of measurement and the minimal detectable change provide a clinically oriented complement to the agreement coefficients, since they express, in the original measurement units, the smallest between-position change that exceeds measurement noise. For most muscles and parameters the minimal detectable change was large relative to the observed standing–prone differences, which means that only sizeable differences could be attributed with confidence to a true position effect rather than to measurement error. These values reinforce the conclusion that, with the notable exception of the lumbar erector spinae decrement, standing and prone MyotonPRO measurements should not be regarded as interchangeable. The present findings should nevertheless be interpreted as preliminary measurement observations rather than as evidence that the MyotonPRO is ready for clinical monitoring of treatment effects in adolescent idiopathic scoliosis: the study did not include a healthy control group, age-matched normative values, correlation with Cobb angle or curve severity, pain scores, patient-reported outcomes, or functional and rehabilitation-response measures. Confirmation in larger, prospectively designed studies that incorporate these elements, together with true same-position repeatability testing, is required before the device can be recommended for routine treatment monitoring in this population.

## 5. Limitations of the Study

Several limitations must be acknowledged. The most important methodological limitation concerns the nature of the primary analysis: the intraclass coefficients reported here quantify the agreement between two different body positions (standing and prone) rather than true same-position intra-rater repeatability. Because the two positions represent different biomechanical and physiological states, a low coefficient may reflect a genuine postural difference rather than poor measurement repeatability, and the present results should therefore be read as estimates of between-position agreement, not of classical intra-rater reliability. A further methodological constraint is that the testing order was fixed, with all participants assessed first in the standing and then in the prone position; because this order was neither randomised nor counterbalanced, genuine position effects cannot be fully separated from order, relaxation, habituation or fatigue effects, and the position-related findings should be interpreted with corresponding caution. The final sample size was constrained to 19 participants due to the strict inclusion criteria and the single-center design of the study, and a formal sample size calculation was not conducted. Consequently, the agreement estimates and wide confidence intervals must be interpreted with caution. It should also be emphasised that the MyotonPRO quantifies the mechanical (viscoelastic) properties of superficial tissues at the measurement site; the present findings therefore describe tissue-level mechanical behaviour rather than deeper neuromuscular activity, and any interpretation in terms of neuromuscular adaptation or functional stabilisation remains indirect and should be made with caution. Furthermore, the cohort predominantly reflects female adolescents (18 females, 1 male), limiting the external validity of the findings regarding the entire AIS population. Due to these sample characteristics, our findings are inherently exploratory. Future multi-center studies with larger, systematically calculated sample sizes are required to validate these outcomes. Additionally, the measurements were conducted under static conditions (standing and prone positions), which may not fully reflect muscle behavior during dynamic activities. The use of a single measurement device, while ensuring standardization, may also introduce device-specific bias.

Another limitation is the potential variability in subjects’ ability to maintain complete relaxation during measurements, which could influence muscle-related outcomes despite standardized instructions. Furthermore, only selected muscle groups (middle and lower trapezius, latissimus dorsi, and lumbar erector spinae) were evaluated, which may not provide a comprehensive overview of overall muscular function or postural balance. Environmental conditions were controlled; however, subtle uncontrolled factors may still have influenced the results.

## 6. Future Research Directions

Future studies should include larger and more diverse populations to improve external validity. The inclusion of multiple examiners would allow for the evaluation of inter-rater reliability. Moreover, future research could incorporate dynamic assessments and functional tasks to better reflect real-life muscle activity. The use of additional measurement tools or multimodal assessment approaches (e.g., imaging or electromyography) could provide more comprehensive insights. Expanding the range of analyzed muscle groups and investigating different postural conditions or clinical populations would further enhance the applicability of the findings.

The absence of a comparative analysis with an age-matched normative group may limit the accuracy of the clinical outcome interpretation; therefore, further studies are warranted.

## 7. Conclusions

When comparing the two testing positions (standing vs. prone), MyotonPRO showed predominantly poor absolute agreement for the superficial back muscles examined in adolescents with S-shaped AIS. Only the decrement of the lumbar erector spinae reached good agreement across positions, while a small number of muscle and parameter combinations—most consistently in the left middle trapezius—showed moderate agreement. Standing versus prone comparisons revealed a clear muscle-specific pattern: the middle trapezius was the most posture-sensitive muscle, showing significant differences in all five parameters bilaterally—with higher tone and stiffness in standing—while both lumbar erectors remained stable across positions. Side-to-side analysis showed that the lower trapezius and latissimus dorsi were more posture-sensitive on the left side (convex for the lumbar curve, concave for the thoracic curve), whereas the right lower trapezius did not differ significantly between positions, indicating that positional changes in superficial back muscles depend not only on the muscle examined but also on curve side in S-shaped AIS. Future research is needed to find out which testing position is more adequate for myotonometer testing in scoliosis.

## Figures and Tables

**Table 1:** Anthropometric characteristics of participants (*N* = 19).

Variable	Mean ± SD
Age (years)	15.3 ± 1.8
Height (cm)	162.5 ± 6.8
Weight (kg)	48.5 ± 6.9
BMI (kg/m^2^)	17.6 ± 1.8
Gender (F/M)	18/1

cm: centimeter; kg: kilogram; BMI: body mass index; SD: standard deviation.

**Table 2:** Agreement between the two testing positions (standing and prone) for MyotonPRO measurements, expressed as intraclass correlation coefficients (two-way mixed-effects, absolute agreement, single measures; ICC(A,1), numerically equivalent to the two-way random-effects ICC(2,1) with 95% confidence intervals. Negative ICC values denote agreement no greater than that expected by chance and are interpreted as poor agreement.

Tested Muscle	Myotonometric Parameters	ICC	95% CI
Right middle trapezius	Tone (Hz)	0.383	−0.111–0.741
	Stiffness (N/m)	0.391	−0.081–0.722
	Decrement	0.347	−0.077–0.680
	Stress relaxation time (ms)	0.401	−0.046–0.719
	Creep	0.383	−0.035–0.699
Left middle trapezius	Tone (Hz)	0.649	0.145–0.864
	Stiffness (N/m)	0.591	0.159–0.825
	Decrement	0.477	0.056–0.758
	Stress relaxation time (ms)	0.567	0.150–0.810
	Creep	0.593	0.179–0.824
Right lower trapezius	Tone (Hz)	0.471	0.057–0.753
	Stiffness (N/m)	0.121	−0.289–0.519
	Decrement	0.528	0.128–0.785
	Stress relaxation time (ms)	−0.045	−0.429–0.385
	Creep	−0.086	−0.481–0.359
Left lower trapezius	Tone (Hz)	0.303	−0.086–0.640
	Stiffness (N/m)	0.340	−0.061–0.666
	Decrement	0.188	−0.220–0.565
	Stress relaxation time (ms)	0.280	−0.098–0.622
	Creep	0.225	−0.115–0.569
Right latissimus dorsi	Tone (Hz)	0.001	−0.308–0.381
	Stiffness (N/m)	0.002	−0.461–0.453
	Decrement	0.335	−0.066–0.664
	Stress relaxation time (ms)	−0.026	−0.483–0.430
	Creep	−0.057	−0.504–0.403
Left latissimus dorsi	Tone (Hz)	0.199	−0.121–0.541
	Stiffness (N/m)	0.230	−0.133–0.579
	Decrement	0.270	−0.102–0.611
	Stress relaxation time (ms)	0.123	−0.217–0.491
	Creep	0.127	−0.181–0.480
Right lumbar erector	Tone (Hz)	0.471	0.032–0.757
	Stiffness (N/m)	0.574	0.163–0.813
	Decrement	0.844	0.642–0.937
	Stress relaxation time (ms)	0.430	−0.008–0.733
	Creep	0.162	−0.258–0.551
Left lumbar erector	Tone (Hz)	0.354	−0.115–0.691
	Stiffness (N/m)	0.416	−0.053–0.729
	Decrement	0.829	0.609–0.930
	Stress relaxation time (ms)	0.421	−0.041–0.731
	Creep	0.054	−0.433–0.498

ICC: intraclass correlation coefficient; CI: confidence interval.

**Table 3:** Comparison of the myotonometric parameters between the two testing positions. Values are mean (SD); *p*-values are derived from paired *t*-tests and effect sizes are expressed as Cohen’s dz (small ≈ 0.2, medium ≈ 0.5, large ≈ 0.8). All comparisons that were significant at *p* < 0.05 remained significant after Benjamini–Hochberg false discovery rate correction. The bold formatting is to emphasize the relevant information.

Tested Muscle	Myotonometric Parameters	Standing	Prone	*p*-Value (Cohen’s dz)
**Right middle trapezius**	Tone (Hz)	16.08 (1.5)	14.37 (1.32)	**<0.0001 (1.45)**
	Stiffness (N/m)	322.4 (60.46)	269.4 (50.75)	**0.0003 (1.01)**
	Decrement	0.94 (0.18)	1.09 (0.15)	**0.0011 (−0.89)**
	Stress relaxation time (ms)	15.98 (2.43)	17.9 (2.28)	**0.0018 (−0.84)**
	Creep	0.97 (0.13)	1.06 (0.11)	**0.0066 (−0.70)**
**Left middle trapezius**	Tone (Hz)	15.75 (1.76)	14.74 (1.79)	**0.0028 (0.80)**
	Stiffness (N/m)	319.8 (66.14)	283.6 (70.69)	**0.0118 (0.64)**
	Decrement	0.98 (0.15)	1.06 (0.12)	**0.0167 (−0.61)**
	Stress relaxation time (ms)	16.03 (2.51)	17.45 (3)	**0.0184 (−0.59)**
	Creep	0.96 (0.13)	1.03 (0.15)	**0.0179 (−0.60)**
**Right lower trapezius**	Tone (Hz)	16.03 (1.82)	15.72 (1.77)	0.184 (0.32)
	Stiffness (N/m)	350.9 (71.25)	316.6 (64.79)	0.112 (0.38)
	Decrement	1.06 (0.1)	1.09 (0.12)	0.199 (−0.31)
	Stress relaxation time (ms)	14.91 (2.24)	16.10 (2.08)	0.117 (−0.38)
	Creep	0.91 (0.11)	0.96 (0.1)	0.172 (−0.33)
**Left lower trapezius**	Tone (Hz)	15.35 (1.55)	16.61 (1.33)	**0.0027 (−0.80)**
	Stiffness (N/m)	323.4 (81.52)	366.6 (56.12)	**0.025 (−0.56)**
	Decrement	1.18 (0.22)	1.08 (0.17)	0.087 (0.42)
	Stress relaxation time (ms)	16.2 (2.41)	14.28 (1.57)	**0.0015 (0.86)**
	Creep	0.98 (0.12)	0.87 (0.07)	**0.007 (0.94)**
**Right latissimus dorsi**	Tone (Hz)	13.51 (1.72)	14.98 (1.78)	**0.0184 (−0.59)**
	Stiffness (N/m)	276.2 (76.36)	292.2 (70.59)	0.509 (−0.15)
	Decrement	1.08 (0.14)	0.97 (0.15)	**0.0056 (0.72)**
	Stress relaxation time (ms)	17.45 (2.76)	16.79 (2.98)	0.489 (0.16)
	Creep	1.01 (0.13)	0.97 (0.12)	0.442 (0.18)
**Left latissimus dorsi**	Tone (Hz)	13.47 (1.9)	15.69 (2.12)	**0.0007 (−0.94)**
	Stiffness (N/m)	272.4 (68.17)	332 (84.6)	**0.0109 (−0.65)**
	Decrement	1.21 (0.17)	1.05 (0.18)	**0.004 (0.76)**
	Stress relaxation time (ms)	18.05 (2.78)	15.78 (3.04)	**0.018 (0.60)**
	Creep	1.07 (0.15)	0.94 (0.13)	**0.0056 (0.72)**
**Right lumbar erector**	Tone (Hz)	12.45 (1.69)	12.72 (1.48)	0.490 (−0.16)
	Stiffness (N/m)	216 (90.08)	215.7 (75.32)	0.987 (0.00)
	Decrement	0.94 (0.22)	0.97 (0.27)	0.493 (−0.16)
	Stress relaxation time (ms)	19.95 (3.87)	20.88 (3.73)	0.329 (−0.23)
	Creep	1.08 (0.18)	1.15 (0.15)	0.125 (−0.37)
**Left lumbar erector**	Tone (Hz)	13.03 (2.76)	13.41 (1.88)	0.537 (−0.14)
	Stiffness (N/m)	256.8 (142.8)	259.3 (92.47)	0.936 (−0.02)
	Decrement	1.00 (0.27)	1.06 (0.3)	0.112 (−0.38)
	Stress relaxation time (ms)	18.84 (4.27)	18.42 (3.55)	0.673 (0.10)
	Creep	1.04 (0.19)	1.03 (0.13)	0.896 (0.03)

**Table 4:** Between-position measurement error and agreement statistics for MyotonPRO measurements (standing versus prone). For each muscle and parameter, the mean difference (standing − prone) with its 95% confidence interval, the 95% Bland–Altman limits of agreement, the standard error of measurement (SEM) and the minimal detectable change at the 95% level (MDC95) are reported in the original measurement units. SEM was calculated as the standard deviation of the differences divided by √2, and MDC95 as 1.96 × √2 × SEM.

Tested Muscle	Myotonometric Parameters	Mean Difference, Standing − Prone (95% CI)	95% Limits of Agreement	SEM	MDC95
Right middle trapezius	Tone (Hz)	1.71 (1.14–2.28)	−0.60–4.02	0.83	2.31
	Stiffness (N/m)	53.00 (27.70–78.30)	−49.88–155.88	37.12	102.88
	Decrement	−0.153 (−0.236–−0.070)	−0.491–0.185	0.122	0.338
	Stress relaxation time (ms)	−1.93 (−3.03–−0.82)	−6.44–2.58	1.63	4.51
	Creep	−0.092 (−0.154–−0.029)	−0.346–0.163	0.092	0.255
Left middle trapezius	Tone (Hz)	1.01 (0.40–1.62)	−1.48–3.50	0.90	2.49
	Stiffness (N/m)	36.12 (9.01–63.23)	−74.11–146.35	39.77	110.23
	Decrement	−0.083 (−0.148–−0.017)	−0.350–0.185	0.096	0.267
	Stress relaxation time (ms)	−1.42 (−2.56–−0.27)	−6.08–3.25	1.68	4.66
	Creep	−0.073 (−0.131–−0.014)	−0.311–0.166	0.086	0.238
Right lower trapezius	Tone (Hz)	0.58 (−0.30–1.46)	−3.00–4.16	1.29	3.58
	Stiffness (N/m)	34.33 (−8.94–77.59)	−141.61–210.26	63.47	175.93
	Decrement	−0.034 (−0.087–0.019)	−0.250–0.182	0.078	0.216
	Stress relaxation time (ms)	−1.18 (−2.70–0.33)	−7.34–4.97	2.22	6.15
	Creep	−0.054 (−0.133–0.026)	−0.376–0.269	0.116	0.323
Left lower trapezius	Tone (Hz)	−1.25 (−2.01–−0.49)	−4.33–1.83	1.11	3.08
	Stiffness (N/m)	−43.22 (−80.37–−6.07)	−194.30–107.85	54.50	151.08
	Decrement	0.105 (−0.017–0.227)	−0.391–0.602	0.179	0.496
	Stress relaxation time (ms)	1.92 (0.84–3.00)	−2.47–6.30	1.58	4.39
	Creep	0.114 (0.055–0.172)	−0.123–0.351	0.086	0.237
Right latissimus dorsi	Tone (Hz)	−1.47 (−2.67–−0.28)	−6.33–3.38	1.75	4.85
	Stiffness (N/m)	−16.04 (−66.11–34.02)	−219.64–187.55	73.45	203.59
	Decrement	0.114 (0.038–0.191)	−0.196–0.425	0.112	0.310
	Stress relaxation time (ms)	0.67 (−1.32–2.65)	−7.40–8.73	2.91	8.06
	Creep	0.034 (−0.057–0.126)	−0.338–0.406	0.134	0.372
Left latissimus dorsi	Tone (Hz)	−2.23 (−3.37–−1.09)	−6.87–2.41	1.68	4.64
	Stiffness (N/m)	−59.59 (−103.72–−15.46)	−239.03–119.85	64.74	179.44
	Decrement	0.155 (0.056–0.254)	−0.247–0.557	0.145	0.402
	Stress relaxation time (ms)	2.27 (0.44–4.09)	−5.17–9.70	2.68	7.44
	Creep	0.132 (0.044–0.220)	−0.227–0.491	0.130	0.359
Right lumbar erector	Tone (Hz)	−0.27 (−1.06–0.53)	−3.50–2.97	1.17	3.23
	Stiffness (N/m)	0.29 (−37.21–37.80)	−152.24–152.82	55.03	152.53
	Decrement	−0.023 (−0.091–0.045)	−0.299–0.254	0.100	0.277
	Stress relaxation time (ms)	−0.93 (−2.89–1.02)	−8.89–7.02	2.87	7.95
	Creep	−0.079 (−0.182–0.024)	−0.499–0.341	0.151	0.420
Left lumbar erector	Tone (Hz)	−0.39 (−1.69–0.91)	−5.68–4.90	1.91	5.29
	Stiffness (N/m)	−2.45 (−65.83–60.92)	−260.16–255.26	92.97	257.71
	Decrement	−0.063 (−0.143–0.016)	−0.386–0.260	0.117	0.323
	Stress relaxation time (ms)	0.42 (−1.64–2.48)	−7.95–8.79	3.02	8.37
	Creep	0.007 (−0.102–0.116)	−0.436–0.450	0.160	0.443

## Data Availability

The original contributions presented in this study are included in the article/Supplementary Material. Further inquiries can be directed to the corresponding author.

## Supplementary Materials

The following supporting information can be downloaded at: https://www.mdpi.com/article/10.3390/jcm15135051/s1. Table S1: Raw data. Agreement of MyotonPRO measurements across standing and prone positions for the biomechanical and viscoelastic properties of superficial back muscles in children and adolescents with idiopathic scoliosis.
