# Supplementary material for: Agreement of MyotonPRO Measurements Across Standing and Prone Positions in Adolescents with Idiopathic Scoliosis
Source: J Clin Med. 2026 Jun 29;15(13):5051. doi: 10.3390/jcm15135051 (PMC13362030; doi:10.3390/jcm15135051)
Supplement: Supplementary file 1 [file jcm-15-05051-s001.zip › jcm-4353544-supplementary.pdf]

Table S1. Raw data. Agreement of MyotonPRO measurements across standing and prone posi-tions for the biomechanical and viscoelastic properties of superficial back muscles in children and adolescents with idiopathic scoliosis.

SubjectID	Muscle	Side	Region	Convexity	Position	Parameter	Unit	Value
S01	Middle trapezius	Left	Thoracic	Concave	Standing	Tone (oscillation frequency)	Hz	14,79
S01	Middle trapezius	Left	Thoracic	Concave	Prone	Tone (oscillation frequency)	Hz	13,2
S01	Middle trapezius	Left	Thoracic	Concave	Standing	Dynamic stiffness	N/m	260,3
S01	Middle trapezius	Left	Thoracic	Concave	Prone	Dynamic stiffness	N/m	238
S01	Middle trapezius	Left	Thoracic	Concave	Standing	Logarithmic decrement (elasticity)	dimensionless	1,09
S01	Middle trapezius	Left	Thoracic	Concave	Prone	Logarithmic decrement (elasticity)	dimensionless	1,22
S01	Middle trapezius	Left	Thoracic	Concave	Standing	Mechanical stress relaxation time	ms	18,46
S01	Middle trapezius	Left	Thoracic	Concave	Prone	Mechanical stress relaxation time	ms	18,96
S01	Middle trapezius	Left	Thoracic	Concave	Standing	Creep (Deborah number ratio)	dimensionless	1,09
S01	Middle trapezius	Left	Thoracic	Concave	Prone	Creep (Deborah number ratio)	dimensionless	1,09
S01	Middle trapezius	Right	Thoracic	Convex	Standing	Tone (oscillation frequency)	Hz	17
S01	Middle trapezius	Right	Thoracic	Convex	Prone	Tone (oscillation frequency)	Hz	14,1
S01	Middle trapezius	Right	Thoracic	Convex	Standing	Dynamic stiffness	N/m	370
S01	Middle trapezius	Right	Thoracic	Convex	Prone	Dynamic stiffness	N/m	298
S01	Middle trapezius	Right	Thoracic	Convex	Standing	Logarithmic decrement (elasticity)	dimensionless	1,17
S01	Middle trapezius	Right	Thoracic	Convex	Prone	Logarithmic decrement (elasticity)	dimensionless	1,46
S01	Middle trapezius	Right	Thoracic	Convex	Standing	Mechanical stress relaxation time	ms	13,95
S01	Middle trapezius	Right	Thoracic	Convex	Prone	Mechanical stress relaxation time	ms	16,84
S01	Middle trapezius	Right	Thoracic	Convex	Standing	Creep (Deborah number ratio)	dimensionless	0,86
S01	Middle trapezius	Right	Thoracic	Convex	Prone	Creep (Deborah number ratio)	dimensionless	1,02
S01	Lower trapezius	Left	Thoracic	Concave	Standing	Tone (oscillation frequency)	Hz	15,8
S01	Lower trapezius	Left	Thoracic	Concave	Prone	Tone (oscillation frequency)	Hz	15,96
S01	Lower trapezius	Left	Thoracic	Concave	Standing	Dynamic stiffness	N/m	341,2
S01	Lower trapezius	Left	Thoracic	Concave	Prone	Dynamic stiffness	N/m	326,4
S01	Lower trapezius	Left	Thoracic	Concave	Standing	Logarithmic decrement (elasticity)	dimensionless	1,41
S01	Lower trapezius	Left	Thoracic	Concave	Prone	Logarithmic decrement (elasticity)	dimensionless	1,2
S01	Lower trapezius	Left	Thoracic	Concave	Standing	Mechanical stress relaxation time	ms	15,16
S01	Lower trapezius	Left	Thoracic	Concave	Prone	Mechanical stress relaxation time	ms	15,42
S01	Lower trapezius	Left	Thoracic	Concave	Standing	Creep (Deborah number ratio)	dimensionless	0,93
S01	Lower trapezius	Left	Thoracic	Concave	Prone	Creep (Deborah number ratio)	dimensionless	0,94
S01	Lower trapezius	Right	Thoracic	Convex	Standing	Tone (oscillation frequency)	Hz	18,42
S01	Lower trapezius	Right	Thoracic	Convex	Prone	Tone (oscillation frequency)	Hz	15,14
S01	Lower trapezius	Right	Thoracic	Convex	Standing	Dynamic stiffness	N/m	427
S01	Lower trapezius	Right	Thoracic	Convex	Prone	Dynamic stiffness	N/m	305,8
S01	Lower trapezius	Right	Thoracic	Convex	Standing	Logarithmic decrement (elasticity)	dimensionless	1,29
S01	Lower trapezius	Right	Thoracic	Convex	Prone	Logarithmic decrement (elasticity)	dimensionless	1,38
S01	Lower trapezius	Right	Thoracic	Convex	Standing	Mechanical stress relaxation time	ms	12,24
S01	Lower trapezius	Right	Thoracic	Convex	Prone	Mechanical stress relaxation time	ms	16,64
S01	Lower trapezius	Right	Thoracic	Convex	Standing	Creep (Deborah number ratio)	dimensionless	0,76
S01	Lower trapezius	Right	Thoracic	Convex	Prone	Creep (Deborah number ratio)	dimensionless	0,99
S01	Latissimus dorsi	Left	Thoracic/thoracolumbar	Concave	Standing	Tone (oscillation frequency)	Hz	15,34
S01	Latissimus dorsi	Left	Thoracic/thoracolumbar	Concave	Prone	Tone (oscillation frequency)	Hz	16,12
S01	Latissimus dorsi	Left	Thoracic/thoracolumbar	Concave	Standing	Dynamic stiffness	N/m	352
S01	Latissimus dorsi	Left	Thoracic/thoracolumbar	Concave	Prone	Dynamic stiffness	N/m	329,2
S01	Latissimus dorsi	Left	Thoracic/thoracolumbar	Concave	Standing	Logarithmic decrement (elasticity)	dimensionless	1,58
S01	Latissimus dorsi	Left	Thoracic/thoracolumbar	Concave	Prone	Logarithmic decrement (elasticity)	dimensionless	1,31
S01	Latissimus dorsi	Left	Thoracic/thoracolumbar	Concave	Standing	Mechanical stress relaxation time	ms	14,9
S01	Latissimus dorsi	Left	Thoracic/thoracolumbar	Concave	Prone	Mechanical stress relaxation time	ms	16,04
S01	Latissimus dorsi	Left	Thoracic/thoracolumbar	Concave	Standing	Creep (Deborah number ratio)	dimensionless	0,92
S01	Latissimus dorsi	Left	Thoracic/thoracolumbar	Concave	Prone	Creep (Deborah number ratio)	dimensionless	0,99
S01	Latissimus dorsi	Right	Thoracic/thoracolumbar	Convex	Standing	Tone (oscillation frequency)	Hz	16,92
S01	Latissimus dorsi	Right	Thoracic/thoracolumbar	Convex	Prone	Tone (oscillation frequency)	Hz	14,74
S01	Latissimus dorsi	Right	Thoracic/thoracolumbar	Convex	Standing	Dynamic stiffness	N/m	427,6
S01	Latissimus dorsi	Right	Thoracic/thoracolumbar	Convex	Prone	Dynamic stiffness	N/m	273,4
S01	Latissimus dorsi	Right	Thoracic/thoracolumbar	Convex	Standing	Logarithmic decrement (elasticity)	dimensionless	1,37
S01	Latissimus dorsi	Right	Thoracic/thoracolumbar	Convex	Prone	Logarithmic decrement (elasticity)	dimensionless	1,12
S01	Latissimus dorsi	Right	Thoracic/thoracolumbar	Convex	Standing	Mechanical stress relaxation time	ms	12,8
S01	Latissimus dorsi	Right	Thoracic/thoracolumbar	Convex	Prone	Mechanical stress relaxation time	ms	17,34
S01	Latissimus dorsi	Right	Thoracic/thoracolumbar	Convex	Standing	Creep (Deborah number ratio)	dimensionless	0,81
S01	Latissimus dorsi	Right	Thoracic/thoracolumbar	Convex	Prone	Creep (Deborah number ratio)	dimensionless	1,03
S01	Lumbar erector spinae	Left	Lumbar	Convex	Standing	Tone (oscillation frequency)	Hz	17,54
S01	Lumbar erector spinae	Left	Lumbar	Convex	Prone	Tone (oscillation frequency)	Hz	14,2
S01	Lumbar erector spinae	Left	Lumbar	Convex	Standing	Dynamic stiffness	N/m	441,2
S01	Lumbar erector spinae	Left	Lumbar	Convex	Prone	Dynamic stiffness	N/m	308,8
S01	Lumbar erector spinae	Left	Lumbar	Convex	Standing	Logarithmic decrement (elasticity)	dimensionless	1,41
S01	Lumbar erector spinae	Left	Lumbar	Convex	Prone	Logarithmic decrement (elasticity)	dimensionless	1,69
S01	Lumbar erector spinae	Left	Lumbar	Convex	Standing	Mechanical stress relaxation time	ms	11,8
S01	Lumbar erector spinae	Left	Lumbar	Convex	Prone	Mechanical stress relaxation time	ms	16,64
S01	Lumbar erector spinae	Left	Lumbar	Convex	Standing	Creep (Deborah number ratio)	dimensionless	0,75
S01	Lumbar erector spinae	Left	Lumbar	Convex	Prone	Creep (Deborah number ratio)	dimensionless	1,02
S01	Lumbar erector spinae	Right	Lumbar	Concave	Standing	Tone (oscillation frequency)	Hz	14,3
S01	Lumbar erector spinae	Right	Lumbar	Concave	Prone	Tone (oscillation frequency)	Hz	15
S01	Lumbar erector spinae	Right	Lumbar	Concave	Standing	Dynamic stiffness	N/m	321,8
S01	Lumbar erector spinae	Right	Lumbar	Concave	Prone	Dynamic stiffness	N/m	363,2
S01	Lumbar erector spinae	Right	Lumbar	Concave	Standing	Logarithmic decrement (elasticity)	dimensionless	1,39
S01	Lumbar erector spinae	Right	Lumbar	Concave	Prone	Logarithmic decrement (elasticity)	dimensionless	1,45
S01	Lumbar erector spinae	Right	Lumbar	Concave	Standing	Mechanical stress relaxation time	ms	15,66
S01	Lumbar erector spinae	Right	Lumbar	Concave	Prone	Mechanical stress relaxation time	ms	14,78
S01	Lumbar erector spinae	Right	Lumbar	Concave	Standing	Creep (Deborah number ratio)	dimensionless	0,96
S01	Lumbar erector spinae	Right	Lumbar	Concave	Prone	Creep (Deborah number ratio)	dimensionless	0,92
S02	Middle trapezius	Left	Thoracic	Concave	Standing	Tone (oscillation frequency)	Hz	17,68
S02	Middle trapezius	Left	Thoracic	Concave	Prone	Tone (oscillation frequency)	Hz	17,12
S02	Middle trapezius	Left	Thoracic	Concave	Standing	Dynamic stiffness	N/m	386
S02	Middle trapezius	Left	Thoracic	Concave	Prone	Dynamic stiffness	N/m	395
S02	Middle trapezius	Left	Thoracic	Concave	Standing	Logarithmic decrement (elasticity)	dimensionless	0,99
S02	Middle trapezius	Left	Thoracic	Concave	Prone	Logarithmic decrement (elasticity)	dimensionless	1,24
S02	Middle trapezius	Left	Thoracic	Concave	Standing	Mechanical stress relaxation time	ms	13,4
S02	Middle trapezius	Left	Thoracic	Concave	Prone	Mechanical stress relaxation time	ms	13,5
S02	Middle trapezius	Left	Thoracic	Concave	Standing	Creep (Deborah number ratio)	dimensionless	0,83

S02	Middle trapezius	Left	Thoracic	Concave	Prone	Creep (Deborah number ratio)	dimensionless	0,84
S02	Middle trapezius	Right	Thoracic	Convex	Standing	Tone (oscillation frequency)	Hz	16,62
S02	Middle trapezius	Right	Thoracic	Convex	Prone	Tone (oscillation frequency)	Hz	15,74
S02	Middle trapezius	Right	Thoracic	Convex	Standing	Dynamic stiffness	N/m	327
S02	Middle trapezius	Right	Thoracic	Convex	Prone	Dynamic stiffness	N/m	327
S02	Middle trapezius	Right	Thoracic	Convex	Standing	Logarithmic decrement (elasticity)	dimensionless	0,9
S02	Middle trapezius	Right	Thoracic	Convex	Prone	Logarithmic decrement (elasticity)	dimensionless	1,04
S02	Middle trapezius	Right	Thoracic	Convex	Standing	Mechanical stress relaxation time	ms	15,8
S02	Middle trapezius	Right	Thoracic	Convex	Prone	Mechanical stress relaxation time	ms	16
S02	Middle trapezius	Right	Thoracic	Convex	Standing	Creep (Deborah number ratio)	dimensionless	0,95
S02	Middle trapezius	Right	Thoracic	Convex	Prone	Creep (Deborah number ratio)	dimensionless	0,97
S02	Lower trapezius	Left	Thoracic	Concave	Standing	Tone (oscillation frequency)	Hz	15,84
S02	Lower trapezius	Left	Thoracic	Concave	Prone	Tone (oscillation frequency)	Hz	17,2
S02	Lower trapezius	Left	Thoracic	Concave	Standing	Dynamic stiffness	N/m	328
S02	Lower trapezius	Left	Thoracic	Concave	Prone	Dynamic stiffness	N/m	365
S02	Lower trapezius	Left	Thoracic	Concave	Standing	Logarithmic decrement (elasticity)	dimensionless	1,1
S02	Lower trapezius	Left	Thoracic	Concave	Prone	Logarithmic decrement (elasticity)	dimensionless	0,99
S02	Lower trapezius	Left	Thoracic	Concave	Standing	Mechanical stress relaxation time	ms	15,3
S02	Lower trapezius	Left	Thoracic	Concave	Prone	Mechanical stress relaxation time	ms	14,4
S02	Lower trapezius	Left	Thoracic	Concave	Standing	Creep (Deborah number ratio)	dimensionless	0,92
S02	Lower trapezius	Left	Thoracic	Concave	Prone	Creep (Deborah number ratio)	dimensionless	0,88
S02	Lower trapezius	Right	Thoracic	Convex	Standing	Tone (oscillation frequency)	Hz	15,4
S02	Lower trapezius	Right	Thoracic	Convex	Prone	Tone (oscillation frequency)	Hz	17,8
S02	Lower trapezius	Right	Thoracic	Convex	Standing	Dynamic stiffness	N/m	305
S02	Lower trapezius	Right	Thoracic	Convex	Prone	Dynamic stiffness	N/m	383
S02	Lower trapezius	Right	Thoracic	Convex	Standing	Logarithmic decrement (elasticity)	dimensionless	1,03
S02	Lower trapezius	Right	Thoracic	Convex	Prone	Logarithmic decrement (elasticity)	dimensionless	1,1
S02	Lower trapezius	Right	Thoracic	Convex	Standing	Mechanical stress relaxation time	ms	16,2
S02	Lower trapezius	Right	Thoracic	Convex	Prone	Mechanical stress relaxation time	ms	14,2
S02	Lower trapezius	Right	Thoracic	Convex	Standing	Creep (Deborah number ratio)	dimensionless	0,97
S02	Lower trapezius	Right	Thoracic	Convex	Prone	Creep (Deborah number ratio)	dimensionless	0,87
S02	Latissimus dorsi	Left	Thoracic/thoracolumbar	Concave	Standing	Tone (oscillation frequency)	Hz	14,92
S02	Latissimus dorsi	Left	Thoracic/thoracolumbar	Concave	Prone	Tone (oscillation frequency)	Hz	17,04
S02	Latissimus dorsi	Left	Thoracic/thoracolumbar	Concave	Standing	Dynamic stiffness	N/m	306
S02	Latissimus dorsi	Left	Thoracic/thoracolumbar	Concave	Prone	Dynamic stiffness	N/m	380
S02	Latissimus dorsi	Left	Thoracic/thoracolumbar	Concave	Standing	Logarithmic decrement (elasticity)	dimensionless	1,12
S02	Latissimus dorsi	Left	Thoracic/thoracolumbar	Concave	Prone	Logarithmic decrement (elasticity)	dimensionless	1,02
S02	Latissimus dorsi	Left	Thoracic/thoracolumbar	Concave	Standing	Mechanical stress relaxation time	ms	16,1
S02	Latissimus dorsi	Left	Thoracic/thoracolumbar	Concave	Prone	Mechanical stress relaxation time	ms	13,9
S02	Latissimus dorsi	Left	Thoracic/thoracolumbar	Concave	Standing	Creep (Deborah number ratio)	dimensionless	0,96
S02	Latissimus dorsi	Left	Thoracic/thoracolumbar	Concave	Prone	Creep (Deborah number ratio)	dimensionless	0,85
S02	Latissimus dorsi	Right	Thoracic/thoracolumbar	Convex	Standing	Tone (oscillation frequency)	Hz	12,86
S02	Latissimus dorsi	Right	Thoracic/thoracolumbar	Convex	Prone	Tone (oscillation frequency)	Hz	16,42
S02	Latissimus dorsi	Right	Thoracic/thoracolumbar	Convex	Standing	Dynamic stiffness	N/m	208
S02	Latissimus dorsi	Right	Thoracic/thoracolumbar	Convex	Prone	Dynamic stiffness	N/m	359
S02	Latissimus dorsi	Right	Thoracic/thoracolumbar	Convex	Standing	Logarithmic decrement (elasticity)	dimensionless	0,95
S02	Latissimus dorsi	Right	Thoracic/thoracolumbar	Convex	Prone	Logarithmic decrement (elasticity)	dimensionless	1,2
S02	Latissimus dorsi	Right	Thoracic/thoracolumbar	Convex	Standing	Mechanical stress relaxation time	ms	18,9
S02	Latissimus dorsi	Right	Thoracic/thoracolumbar	Convex	Prone	Mechanical stress relaxation time	ms	14,8
S02	Latissimus dorsi	Right	Thoracic/thoracolumbar	Convex	Standing	Creep (Deborah number ratio)	dimensionless	1,03
S02	Latissimus dorsi	Right	Thoracic/thoracolumbar	Convex	Prone	Creep (Deborah number ratio)	dimensionless	0,91
S02	Lumbar erector spinae	Left	Lumbar	Convex	Standing	Tone (oscillation frequency)	Hz	11,36
S02	Lumbar erector spinae	Left	Lumbar	Convex	Prone	Tone (oscillation frequency)	Hz	14,02
S02	Lumbar erector spinae	Left	Lumbar	Convex	Standing	Dynamic stiffness	N/m	183
S02	Lumbar erector spinae	Left	Lumbar	Convex	Prone	Dynamic stiffness	N/m	308
S02	Lumbar erector spinae	Left	Lumbar	Convex	Standing	Logarithmic decrement (elasticity)	dimensionless	1,2
S02	Lumbar erector spinae	Left	Lumbar	Convex	Prone	Logarithmic decrement (elasticity)	dimensionless	1,2
S02	Lumbar erector spinae	Left	Lumbar	Convex	Standing	Mechanical stress relaxation time	ms	22,1
S02	Lumbar erector spinae	Left	Lumbar	Convex	Prone	Mechanical stress relaxation time	ms	16,2
S02	Lumbar erector spinae	Left	Lumbar	Convex	Standing	Creep (Deborah number ratio)	dimensionless	1,26
S02	Lumbar erector spinae	Left	Lumbar	Convex	Prone	Creep (Deborah number ratio)	dimensionless	0,99
S02	Lumbar erector spinae	Right	Lumbar	Concave	Standing	Tone (oscillation frequency)	Hz	11,58
S02	Lumbar erector spinae	Right	Lumbar	Concave	Prone	Tone (oscillation frequency)	Hz	14,62
S02	Lumbar erector spinae	Right	Lumbar	Concave	Standing	Dynamic stiffness	N/m	168
S02	Lumbar erector spinae	Right	Lumbar	Concave	Prone	Dynamic stiffness	N/m	303
S02	Lumbar erector spinae	Right	Lumbar	Concave	Standing	Logarithmic decrement (elasticity)	dimensionless	0,95
S02	Lumbar erector spinae	Right	Lumbar	Concave	Prone	Logarithmic decrement (elasticity)	dimensionless	1,18
S02	Lumbar erector spinae	Right	Lumbar	Concave	Standing	Mechanical stress relaxation time	ms	22,6
S02	Lumbar erector spinae	Right	Lumbar	Concave	Prone	Mechanical stress relaxation time	ms	17
S02	Lumbar erector spinae	Right	Lumbar	Concave	Standing	Creep (Deborah number ratio)	dimensionless	1,3
S02	Lumbar erector spinae	Right	Lumbar	Concave	Prone	Creep (Deborah number ratio)	dimensionless	1,04
S03	Middle trapezius	Left	Thoracic	Concave	Standing	Tone (oscillation frequency)	Hz	19,1
S03	Middle trapezius	Left	Thoracic	Concave	Prone	Tone (oscillation frequency)	Hz	18,8
S03	Middle trapezius	Left	Thoracic	Concave	Standing	Dynamic stiffness	N/m	434
S03	Middle trapezius	Left	Thoracic	Concave	Prone	Dynamic stiffness	N/m	435
S03	Middle trapezius	Left	Thoracic	Concave	Standing	Logarithmic decrement (elasticity)	dimensionless	1,11
S03	Middle trapezius	Left	Thoracic	Concave	Prone	Logarithmic decrement (elasticity)	dimensionless	1,04
S03	Middle trapezius	Left	Thoracic	Concave	Standing	Mechanical stress relaxation time	ms	12,5
S03	Middle trapezius	Left	Thoracic	Concave	Prone	Mechanical stress relaxation time	ms	12,6
S03	Middle trapezius	Left	Thoracic	Concave	Standing	Creep (Deborah number ratio)	dimensionless	0,78
S03	Middle trapezius	Left	Thoracic	Concave	Prone	Creep (Deborah number ratio)	dimensionless	0,79
S03	Middle trapezius	Right	Thoracic	Convex	Standing	Tone (oscillation frequency)	Hz	19
S03	Middle trapezius	Right	Thoracic	Convex	Prone	Tone (oscillation frequency)	Hz	16,5
S03	Middle trapezius	Right	Thoracic	Convex	Standing	Dynamic stiffness	N/m	436
S03	Middle trapezius	Right	Thoracic	Convex	Prone	Dynamic stiffness	N/m	330
S03	Middle trapezius	Right	Thoracic	Convex	Standing	Logarithmic decrement (elasticity)	dimensionless	1,12
S03	Middle trapezius	Right	Thoracic	Convex	Prone	Logarithmic decrement (elasticity)	dimensionless	0,92
S03	Middle trapezius	Right	Thoracic	Convex	Standing	Mechanical stress relaxation time	ms	12,6
S03	Middle trapezius	Right	Thoracic	Convex	Prone	Mechanical stress relaxation time	ms	15,3
S03	Middle trapezius	Right	Thoracic	Convex	Standing	Creep (Deborah number ratio)	dimensionless	0,79

S03	Middle trapezius	Right	Thoracic	Convex	Prone	Creep (Deborah number ratio)	dimensionless	0,93
S03	Lower trapezius	Left	Thoracic	Concave	Standing	Tone (oscillation frequency)	Hz	19,9
S03	Lower trapezius	Left	Thoracic	Concave	Prone	Tone (oscillation frequency)	Hz	17,7
S03	Lower trapezius	Left	Thoracic	Concave	Standing	Dynamic stiffness	N/m	495
S03	Lower trapezius	Left	Thoracic	Concave	Prone	Dynamic stiffness	N/m	411
S03	Lower trapezius	Left	Thoracic	Concave	Standing	Logarithmic decrement (elasticity)	dimensionless	1,35
S03	Lower trapezius	Left	Thoracic	Concave	Prone	Logarithmic decrement (elasticity)	dimensionless	1,24
S03	Lower trapezius	Left	Thoracic	Concave	Standing	Mechanical stress relaxation time	ms	11,6
S03	Lower trapezius	Left	Thoracic	Concave	Prone	Mechanical stress relaxation time	ms	13
S03	Lower trapezius	Left	Thoracic	Concave	Standing	Creep (Deborah number ratio)	dimensionless	0,75
S03	Lower trapezius	Left	Thoracic	Concave	Prone	Creep (Deborah number ratio)	dimensionless	0,83
S03	Lower trapezius	Right	Thoracic	Convex	Standing	Tone (oscillation frequency)	Hz	22,3
S03	Lower trapezius	Right	Thoracic	Convex	Prone	Tone (oscillation frequency)	Hz	20,7
S03	Lower trapezius	Right	Thoracic	Convex	Standing	Dynamic stiffness	N/m	507
S03	Lower trapezius	Right	Thoracic	Convex	Prone	Dynamic stiffness	N/m	494
S03	Lower trapezius	Right	Thoracic	Convex	Standing	Logarithmic decrement (elasticity)	dimensionless	1,01
S03	Lower trapezius	Right	Thoracic	Convex	Prone	Logarithmic decrement (elasticity)	dimensionless	1,17
S03	Lower trapezius	Right	Thoracic	Convex	Standing	Mechanical stress relaxation time	ms	10,3
S03	Lower trapezius	Right	Thoracic	Convex	Prone	Mechanical stress relaxation time	ms	11,5
S03	Lower trapezius	Right	Thoracic	Convex	Standing	Creep (Deborah number ratio)	dimensionless	0,66
S03	Lower trapezius	Right	Thoracic	Convex	Prone	Creep (Deborah number ratio)	dimensionless	0,74
S03	Latissimus dorsi	Left	Thoracic/thoracolumbar	Concave	Standing	Tone (oscillation frequency)	Hz	19,3
S03	Latissimus dorsi	Left	Thoracic/thoracolumbar	Concave	Prone	Tone (oscillation frequency)	Hz	18,4
S03	Latissimus dorsi	Left	Thoracic/thoracolumbar	Concave	Standing	Dynamic stiffness	N/m	482
S03	Latissimus dorsi	Left	Thoracic/thoracolumbar	Concave	Prone	Dynamic stiffness	N/m	450
S03	Latissimus dorsi	Left	Thoracic/thoracolumbar	Concave	Standing	Logarithmic decrement (elasticity)	dimensionless	1,36
S03	Latissimus dorsi	Left	Thoracic/thoracolumbar	Concave	Prone	Logarithmic decrement (elasticity)	dimensionless	1,43
S03	Latissimus dorsi	Left	Thoracic/thoracolumbar	Concave	Standing	Mechanical stress relaxation time	ms	11,5
S03	Latissimus dorsi	Left	Thoracic/thoracolumbar	Concave	Prone	Mechanical stress relaxation time	ms	12,1
S03	Latissimus dorsi	Left	Thoracic/thoracolumbar	Concave	Standing	Creep (Deborah number ratio)	dimensionless	0,73
S03	Latissimus dorsi	Left	Thoracic/thoracolumbar	Concave	Prone	Creep (Deborah number ratio)	dimensionless	0,77
S03	Latissimus dorsi	Right	Thoracic/thoracolumbar	Convex	Standing	Tone (oscillation frequency)	Hz	16,9
S03	Latissimus dorsi	Right	Thoracic/thoracolumbar	Convex	Prone	Tone (oscillation frequency)	Hz	15,8
S03	Latissimus dorsi	Right	Thoracic/thoracolumbar	Convex	Standing	Dynamic stiffness	N/m	409
S03	Latissimus dorsi	Right	Thoracic/thoracolumbar	Convex	Prone	Dynamic stiffness	N/m	342
S03	Latissimus dorsi	Right	Thoracic/thoracolumbar	Convex	Standing	Logarithmic decrement (elasticity)	dimensionless	1,17
S03	Latissimus dorsi	Right	Thoracic/thoracolumbar	Convex	Prone	Logarithmic decrement (elasticity)	dimensionless	1,19
S03	Latissimus dorsi	Right	Thoracic/thoracolumbar	Convex	Standing	Mechanical stress relaxation time	ms	12,9
S03	Latissimus dorsi	Right	Thoracic/thoracolumbar	Convex	Prone	Mechanical stress relaxation time	ms	14,7
S03	Latissimus dorsi	Right	Thoracic/thoracolumbar	Convex	Standing	Creep (Deborah number ratio)	dimensionless	0,79
S03	Latissimus dorsi	Right	Thoracic/thoracolumbar	Convex	Prone	Creep (Deborah number ratio)	dimensionless	0,9
S03	Lumbar erector spinae	Left	Lumbar	Convex	Standing	Tone (oscillation frequency)	Hz	13,8
S03	Lumbar erector spinae	Left	Lumbar	Convex	Prone	Tone (oscillation frequency)	Hz	16,4
S03	Lumbar erector spinae	Left	Lumbar	Convex	Standing	Dynamic stiffness	N/m	372
S03	Lumbar erector spinae	Left	Lumbar	Convex	Prone	Dynamic stiffness	N/m	435
S03	Lumbar erector spinae	Left	Lumbar	Convex	Standing	Logarithmic decrement (elasticity)	dimensionless	1,21
S03	Lumbar erector spinae	Left	Lumbar	Convex	Prone	Logarithmic decrement (elasticity)	dimensionless	1,34
S03	Lumbar erector spinae	Left	Lumbar	Convex	Standing	Mechanical stress relaxation time	ms	15,1
S03	Lumbar erector spinae	Left	Lumbar	Convex	Prone	Mechanical stress relaxation time	ms	12,7
S03	Lumbar erector spinae	Left	Lumbar	Convex	Standing	Creep (Deborah number ratio)	dimensionless	0,92
S03	Lumbar erector spinae	Left	Lumbar	Convex	Prone	Creep (Deborah number ratio)	dimensionless	0,8
S03	Lumbar erector spinae	Right	Lumbar	Concave	Standing	Tone (oscillation frequency)	Hz	15,4
S03	Lumbar erector spinae	Right	Lumbar	Concave	Prone	Tone (oscillation frequency)	Hz	12,9
S03	Lumbar erector spinae	Right	Lumbar	Concave	Standing	Dynamic stiffness	N/m	398
S03	Lumbar erector spinae	Right	Lumbar	Concave	Prone	Dynamic stiffness	N/m	272
S03	Lumbar erector spinae	Right	Lumbar	Concave	Standing	Logarithmic decrement (elasticity)	dimensionless	1,08
S03	Lumbar erector spinae	Right	Lumbar	Concave	Prone	Logarithmic decrement (elasticity)	dimensionless	1,07
S03	Lumbar erector spinae	Right	Lumbar	Concave	Standing	Mechanical stress relaxation time	ms	13,4
S03	Lumbar erector spinae	Right	Lumbar	Concave	Prone	Mechanical stress relaxation time	ms	18,9
S03	Lumbar erector spinae	Right	Lumbar	Concave	Standing	Creep (Deborah number ratio)	dimensionless	0,84
S03	Lumbar erector spinae	Right	Lumbar	Concave	Prone	Creep (Deborah number ratio)	dimensionless	1,13
S04	Middle trapezius	Left	Thoracic	Concave	Standing	Tone (oscillation frequency)	Hz	18,3
S04	Middle trapezius	Left	Thoracic	Concave	Prone	Tone (oscillation frequency)	Hz	15,6
S04	Middle trapezius	Left	Thoracic	Concave	Standing	Dynamic stiffness	N/m	421
S04	Middle trapezius	Left	Thoracic	Concave	Prone	Dynamic stiffness	N/m	311
S04	Middle trapezius	Left	Thoracic	Concave	Standing	Logarithmic decrement (elasticity)	dimensionless	1,13
S04	Middle trapezius	Left	Thoracic	Concave	Prone	Logarithmic decrement (elasticity)	dimensionless	1,24
S04	Middle trapezius	Left	Thoracic	Concave	Standing	Mechanical stress relaxation time	ms	13
S04	Middle trapezius	Left	Thoracic	Concave	Prone	Mechanical stress relaxation time	ms	15,8
S04	Middle trapezius	Left	Thoracic	Concave	Standing	Creep (Deborah number ratio)	dimensionless	0,83
S04	Middle trapezius	Left	Thoracic	Concave	Prone	Creep (Deborah number ratio)	dimensionless	0,97
S04	Middle trapezius	Right	Thoracic	Convex	Standing	Tone (oscillation frequency)	Hz	17,1
S04	Middle trapezius	Right	Thoracic	Convex	Prone	Tone (oscillation frequency)	Hz	16
S04	Middle trapezius	Right	Thoracic	Convex	Standing	Dynamic stiffness	N/m	358
S04	Middle trapezius	Right	Thoracic	Convex	Prone	Dynamic stiffness	N/m	331
S04	Middle trapezius	Right	Thoracic	Convex	Standing	Logarithmic decrement (elasticity)	dimensionless	0,99
S04	Middle trapezius	Right	Thoracic	Convex	Prone	Logarithmic decrement (elasticity)	dimensionless	1,24
S04	Middle trapezius	Right	Thoracic	Convex	Standing	Mechanical stress relaxation time	ms	14,4
S04	Middle trapezius	Right	Thoracic	Convex	Prone	Mechanical stress relaxation time	ms	14,7
S04	Middle trapezius	Right	Thoracic	Convex	Standing	Creep (Deborah number ratio)	dimensionless	0,88
S04	Middle trapezius	Right	Thoracic	Convex	Prone	Creep (Deborah number ratio)	dimensionless	0,89
S04	Lower trapezius	Left	Thoracic	Concave	Standing	Tone (oscillation frequency)	Hz	15,9
S04	Lower trapezius	Left	Thoracic	Concave	Prone	Tone (oscillation frequency)	Hz	15,8
S04	Lower trapezius	Left	Thoracic	Concave	Standing	Dynamic stiffness	N/m	332
S04	Lower trapezius	Left	Thoracic	Concave	Prone	Dynamic stiffness	N/m	307
S04	Lower trapezius	Left	Thoracic	Concave	Standing	Logarithmic decrement (elasticity)	dimensionless	1,34
S04	Lower trapezius	Left	Thoracic	Concave	Prone	Logarithmic decrement (elasticity)	dimensionless	1,27
S04	Lower trapezius	Left	Thoracic	Concave	Standing	Mechanical stress relaxation time	ms	15,5
S04	Lower trapezius	Left	Thoracic	Concave	Prone	Mechanical stress relaxation time	ms	15,6
S04	Lower trapezius	Left	Thoracic	Concave	Standing	Creep (Deborah number ratio)	dimensionless	0,95

S04	Lower trapezius	Left	Thoracic	Concave	Prone	Creep (Deborah number ratio)	dimensionless	0,95
S04	Lower trapezius	Right	Thoracic	Convex	Standing	Tone (oscillation frequency)	Hz	16,6
S04	Lower trapezius	Right	Thoracic	Convex	Prone	Tone (oscillation frequency)	Hz	16,3
S04	Lower trapezius	Right	Thoracic	Convex	Standing	Dynamic stiffness	N/m	329
S04	Lower trapezius	Right	Thoracic	Convex	Prone	Dynamic stiffness	N/m	326
S04	Lower trapezius	Right	Thoracic	Convex	Standing	Logarithmic decrement (elasticity)	dimensionless	1,13
S04	Lower trapezius	Right	Thoracic	Convex	Prone	Logarithmic decrement (elasticity)	dimensionless	1,26
S04	Lower trapezius	Right	Thoracic	Convex	Standing	Mechanical stress relaxation time	ms	15,2
S04	Lower trapezius	Right	Thoracic	Convex	Prone	Mechanical stress relaxation time	ms	15,3
S04	Lower trapezius	Right	Thoracic	Convex	Standing	Creep (Deborah number ratio)	dimensionless	0,92
S04	Lower trapezius	Right	Thoracic	Convex	Prone	Creep (Deborah number ratio)	dimensionless	0,93
S04	Latissimus dorsi	Left	Thoracic/thoracolumbar	Concave	Standing	Tone (oscillation frequency)	Hz	13,3
S04	Latissimus dorsi	Left	Thoracic/thoracolumbar	Concave	Prone	Tone (oscillation frequency)	Hz	16,9
S04	Latissimus dorsi	Left	Thoracic/thoracolumbar	Concave	Standing	Dynamic stiffness	N/m	296
S04	Latissimus dorsi	Left	Thoracic/thoracolumbar	Concave	Prone	Dynamic stiffness	N/m	346
S04	Latissimus dorsi	Left	Thoracic/thoracolumbar	Concave	Standing	Logarithmic decrement (elasticity)	dimensionless	1,4
S04	Latissimus dorsi	Left	Thoracic/thoracolumbar	Concave	Prone	Logarithmic decrement (elasticity)	dimensionless	1,14
S04	Latissimus dorsi	Left	Thoracic/thoracolumbar	Concave	Standing	Mechanical stress relaxation time	ms	16,5
S04	Latissimus dorsi	Left	Thoracic/thoracolumbar	Concave	Prone	Mechanical stress relaxation time	ms	14,4
S04	Latissimus dorsi	Left	Thoracic/thoracolumbar	Concave	Standing	Creep (Deborah number ratio)	dimensionless	1
S04	Latissimus dorsi	Left	Thoracic/thoracolumbar	Concave	Prone	Creep (Deborah number ratio)	dimensionless	0,89
S04	Latissimus dorsi	Right	Thoracic/thoracolumbar	Convex	Standing	Tone (oscillation frequency)	Hz	12,3
S04	Latissimus dorsi	Right	Thoracic/thoracolumbar	Convex	Prone	Tone (oscillation frequency)	Hz	17,3
S04	Latissimus dorsi	Right	Thoracic/thoracolumbar	Convex	Standing	Dynamic stiffness	N/m	237
S04	Latissimus dorsi	Right	Thoracic/thoracolumbar	Convex	Prone	Dynamic stiffness	N/m	344
S04	Latissimus dorsi	Right	Thoracic/thoracolumbar	Convex	Standing	Logarithmic decrement (elasticity)	dimensionless	1,22
S04	Latissimus dorsi	Right	Thoracic/thoracolumbar	Convex	Prone	Logarithmic decrement (elasticity)	dimensionless	1,13
S04	Latissimus dorsi	Right	Thoracic/thoracolumbar	Convex	Standing	Mechanical stress relaxation time	ms	19,8
S04	Latissimus dorsi	Right	Thoracic/thoracolumbar	Convex	Prone	Mechanical stress relaxation time	ms	14,8
S04	Latissimus dorsi	Right	Thoracic/thoracolumbar	Convex	Standing	Creep (Deborah number ratio)	dimensionless	1,18
S04	Latissimus dorsi	Right	Thoracic/thoracolumbar	Convex	Prone	Creep (Deborah number ratio)	dimensionless	0,91
S04	Lumbar erector spinae	Left	Lumbar	Convex	Standing	Tone (oscillation frequency)	Hz	13,9
S04	Lumbar erector spinae	Left	Lumbar	Convex	Prone	Tone (oscillation frequency)	Hz	13,9
S04	Lumbar erector spinae	Left	Lumbar	Convex	Standing	Dynamic stiffness	N/m	299
S04	Lumbar erector spinae	Left	Lumbar	Convex	Prone	Dynamic stiffness	N/m	299
S04	Lumbar erector spinae	Left	Lumbar	Convex	Standing	Logarithmic decrement (elasticity)	dimensionless	1,34
S04	Lumbar erector spinae	Left	Lumbar	Convex	Prone	Logarithmic decrement (elasticity)	dimensionless	1,34
S04	Lumbar erector spinae	Left	Lumbar	Convex	Standing	Mechanical stress relaxation time	ms	16,5
S04	Lumbar erector spinae	Left	Lumbar	Convex	Prone	Mechanical stress relaxation time	ms	16,5
S04	Lumbar erector spinae	Left	Lumbar	Convex	Standing	Creep (Deborah number ratio)	dimensionless	1,01
S04	Lumbar erector spinae	Left	Lumbar	Convex	Prone	Creep (Deborah number ratio)	dimensionless	1,01
S04	Lumbar erector spinae	Right	Lumbar	Concave	Standing	Tone (oscillation frequency)	Hz	14,8
S04	Lumbar erector spinae	Right	Lumbar	Concave	Prone	Tone (oscillation frequency)	Hz	14,8
S04	Lumbar erector spinae	Right	Lumbar	Concave	Standing	Dynamic stiffness	N/m	316
S04	Lumbar erector spinae	Right	Lumbar	Concave	Prone	Dynamic stiffness	N/m	316
S04	Lumbar erector spinae	Right	Lumbar	Concave	Standing	Logarithmic decrement (elasticity)	dimensionless	1,4
S04	Lumbar erector spinae	Right	Lumbar	Concave	Prone	Logarithmic decrement (elasticity)	dimensionless	1,4
S04	Lumbar erector spinae	Right	Lumbar	Concave	Standing	Mechanical stress relaxation time	ms	15,9
S04	Lumbar erector spinae	Right	Lumbar	Concave	Prone	Mechanical stress relaxation time	ms	15,9
S04	Lumbar erector spinae	Right	Lumbar	Concave	Standing	Creep (Deborah number ratio)	dimensionless	0,99
S04	Lumbar erector spinae	Right	Lumbar	Concave	Prone	Creep (Deborah number ratio)	dimensionless	0,99
S05	Middle trapezius	Left	Thoracic	Concave	Standing	Tone (oscillation frequency)	Hz	13,2
S05	Middle trapezius	Left	Thoracic	Concave	Prone	Tone (oscillation frequency)	Hz	14,2
S05	Middle trapezius	Left	Thoracic	Concave	Standing	Dynamic stiffness	N/m	253
S05	Middle trapezius	Left	Thoracic	Concave	Prone	Dynamic stiffness	N/m	282
S05	Middle trapezius	Left	Thoracic	Concave	Standing	Logarithmic decrement (elasticity)	dimensionless	1
S05	Middle trapezius	Left	Thoracic	Concave	Prone	Logarithmic decrement (elasticity)	dimensionless	1,03
S05	Middle trapezius	Left	Thoracic	Concave	Standing	Mechanical stress relaxation time	ms	18,6
S05	Middle trapezius	Left	Thoracic	Concave	Prone	Mechanical stress relaxation time	ms	17,3
S05	Middle trapezius	Left	Thoracic	Concave	Standing	Creep (Deborah number ratio)	dimensionless	1,1
S05	Middle trapezius	Left	Thoracic	Concave	Prone	Creep (Deborah number ratio)	dimensionless	1,05
S05	Middle trapezius	Right	Thoracic	Convex	Standing	Tone (oscillation frequency)	Hz	14
S05	Middle trapezius	Right	Thoracic	Convex	Prone	Tone (oscillation frequency)	Hz	13,7
S05	Middle trapezius	Right	Thoracic	Convex	Standing	Dynamic stiffness	N/m	280
S05	Middle trapezius	Right	Thoracic	Convex	Prone	Dynamic stiffness	N/m	266
S05	Middle trapezius	Right	Thoracic	Convex	Standing	Logarithmic decrement (elasticity)	dimensionless	0,95
S05	Middle trapezius	Right	Thoracic	Convex	Prone	Logarithmic decrement (elasticity)	dimensionless	0,94
S05	Middle trapezius	Right	Thoracic	Convex	Standing	Mechanical stress relaxation time	ms	17,4
S05	Middle trapezius	Right	Thoracic	Convex	Prone	Mechanical stress relaxation time	ms	17,4
S05	Middle trapezius	Right	Thoracic	Convex	Standing	Creep (Deborah number ratio)	dimensionless	1,03
S05	Middle trapezius	Right	Thoracic	Convex	Prone	Creep (Deborah number ratio)	dimensionless	1,01
S05	Lower trapezius	Left	Thoracic	Concave	Standing	Tone (oscillation frequency)	Hz	14,4
S05	Lower trapezius	Left	Thoracic	Concave	Prone	Tone (oscillation frequency)	Hz	16,9
S05	Lower trapezius	Left	Thoracic	Concave	Standing	Dynamic stiffness	N/m	294
S05	Lower trapezius	Left	Thoracic	Concave	Prone	Dynamic stiffness	N/m	361
S05	Lower trapezius	Left	Thoracic	Concave	Standing	Logarithmic decrement (elasticity)	dimensionless	1,04
S05	Lower trapezius	Left	Thoracic	Concave	Prone	Logarithmic decrement (elasticity)	dimensionless	0,98
S05	Lower trapezius	Left	Thoracic	Concave	Standing	Mechanical stress relaxation time	ms	16,5
S05	Lower trapezius	Left	Thoracic	Concave	Prone	Mechanical stress relaxation time	ms	14
S05	Lower trapezius	Left	Thoracic	Concave	Standing	Creep (Deborah number ratio)	dimensionless	1
S05	Lower trapezius	Left	Thoracic	Concave	Prone	Creep (Deborah number ratio)	dimensionless	0,86
S05	Lower trapezius	Right	Thoracic	Convex	Standing	Tone (oscillation frequency)	Hz	14
S05	Lower trapezius	Right	Thoracic	Convex	Prone	Tone (oscillation frequency)	Hz	16,4
S05	Lower trapezius	Right	Thoracic	Convex	Standing	Dynamic stiffness	N/m	278
S05	Lower trapezius	Right	Thoracic	Convex	Prone	Dynamic stiffness	N/m	364
S05	Lower trapezius	Right	Thoracic	Convex	Standing	Logarithmic decrement (elasticity)	dimensionless	1,02
S05	Lower trapezius	Right	Thoracic	Convex	Prone	Logarithmic decrement (elasticity)	dimensionless	1
S05	Lower trapezius	Right	Thoracic	Convex	Standing	Mechanical stress relaxation time	ms	16,9
S05	Lower trapezius	Right	Thoracic	Convex	Prone	Mechanical stress relaxation time	ms	14,3
S05	Lower trapezius	Right	Thoracic	Convex	Standing	Creep (Deborah number ratio)	dimensionless	0,98

S05	Lower trapezius	Right	Thoracic	Convex	Prone	Creep (Deborah number ratio)	dimensionless	0,88
S05	Latissimus dorsi	Left	Thoracic/thoracolumbar	Concave	Standing	Tone (oscillation frequency)	Hz	13,5
S05	Latissimus dorsi	Left	Thoracic/thoracolumbar	Concave	Prone	Tone (oscillation frequency)	Hz	16,3
S05	Latissimus dorsi	Left	Thoracic/thoracolumbar	Concave	Standing	Dynamic stiffness	N/m	267
S05	Latissimus dorsi	Left	Thoracic/thoracolumbar	Concave	Prone	Dynamic stiffness	N/m	329
S05	Latissimus dorsi	Left	Thoracic/thoracolumbar	Concave	Standing	Logarithmic decrement (elasticity)	dimensionless	1,07
S05	Latissimus dorsi	Left	Thoracic/thoracolumbar	Concave	Prone	Logarithmic decrement (elasticity)	dimensionless	0,87
S05	Latissimus dorsi	Left	Thoracic/thoracolumbar	Concave	Standing	Mechanical stress relaxation time	ms	17,4
S05	Latissimus dorsi	Left	Thoracic/thoracolumbar	Concave	Prone	Mechanical stress relaxation time	ms	14,9
S05	Latissimus dorsi	Left	Thoracic/thoracolumbar	Concave	Standing	Creep (Deborah number ratio)	dimensionless	1,03
S05	Latissimus dorsi	Left	Thoracic/thoracolumbar	Concave	Prone	Creep (Deborah number ratio)	dimensionless	0,87
S05	Latissimus dorsi	Right	Thoracic/thoracolumbar	Convex	Standing	Tone (oscillation frequency)	Hz	13,8
S05	Latissimus dorsi	Right	Thoracic/thoracolumbar	Convex	Prone	Tone (oscillation frequency)	Hz	14,5
S05	Latissimus dorsi	Right	Thoracic/thoracolumbar	Convex	Standing	Dynamic stiffness	N/m	277
S05	Latissimus dorsi	Right	Thoracic/thoracolumbar	Convex	Prone	Dynamic stiffness	N/m	295
S05	Latissimus dorsi	Right	Thoracic/thoracolumbar	Convex	Standing	Logarithmic decrement (elasticity)	dimensionless	1,01
S05	Latissimus dorsi	Right	Thoracic/thoracolumbar	Convex	Prone	Logarithmic decrement (elasticity)	dimensionless	1
S05	Latissimus dorsi	Right	Thoracic/thoracolumbar	Convex	Standing	Mechanical stress relaxation time	ms	16,4
S05	Latissimus dorsi	Right	Thoracic/thoracolumbar	Convex	Prone	Mechanical stress relaxation time	ms	16,4
S05	Latissimus dorsi	Right	Thoracic/thoracolumbar	Convex	Standing	Creep (Deborah number ratio)	dimensionless	0,94
S05	Latissimus dorsi	Right	Thoracic/thoracolumbar	Convex	Prone	Creep (Deborah number ratio)	dimensionless	0,98
S05	Lumbar erector spinae	Left	Lumbar	Convex	Standing	Tone (oscillation frequency)	Hz	13,7
S05	Lumbar erector spinae	Left	Lumbar	Convex	Prone	Tone (oscillation frequency)	Hz	14,4
S05	Lumbar erector spinae	Left	Lumbar	Convex	Standing	Dynamic stiffness	N/m	216
S05	Lumbar erector spinae	Left	Lumbar	Convex	Prone	Dynamic stiffness	N/m	243
S05	Lumbar erector spinae	Left	Lumbar	Convex	Standing	Logarithmic decrement (elasticity)	dimensionless	0,84
S05	Lumbar erector spinae	Left	Lumbar	Convex	Prone	Logarithmic decrement (elasticity)	dimensionless	1,01
S05	Lumbar erector spinae	Left	Lumbar	Convex	Standing	Mechanical stress relaxation time	ms	19,5
S05	Lumbar erector spinae	Left	Lumbar	Convex	Prone	Mechanical stress relaxation time	ms	17,5
S05	Lumbar erector spinae	Left	Lumbar	Convex	Standing	Creep (Deborah number ratio)	dimensionless	1,07
S05	Lumbar erector spinae	Left	Lumbar	Convex	Prone	Creep (Deborah number ratio)	dimensionless	0,99
S05	Lumbar erector spinae	Right	Lumbar	Concave	Standing	Tone (oscillation frequency)	Hz	13,3
S05	Lumbar erector spinae	Right	Lumbar	Concave	Prone	Tone (oscillation frequency)	Hz	13,3
S05	Lumbar erector spinae	Right	Lumbar	Concave	Standing	Dynamic stiffness	N/m	176
S05	Lumbar erector spinae	Right	Lumbar	Concave	Prone	Dynamic stiffness	N/m	200
S05	Lumbar erector spinae	Right	Lumbar	Concave	Standing	Logarithmic decrement (elasticity)	dimensionless	0,76
S05	Lumbar erector spinae	Right	Lumbar	Concave	Prone	Logarithmic decrement (elasticity)	dimensionless	1,08
S05	Lumbar erector spinae	Right	Lumbar	Concave	Standing	Mechanical stress relaxation time	ms	20,3
S05	Lumbar erector spinae	Right	Lumbar	Concave	Prone	Mechanical stress relaxation time	ms	19,7
S05	Lumbar erector spinae	Right	Lumbar	Concave	Standing	Creep (Deborah number ratio)	dimensionless	1,06
S05	Lumbar erector spinae	Right	Lumbar	Concave	Prone	Creep (Deborah number ratio)	dimensionless	1,09
S06	Middle trapezius	Left	Thoracic	Concave	Standing	Tone (oscillation frequency)	Hz	15,1
S06	Middle trapezius	Left	Thoracic	Concave	Prone	Tone (oscillation frequency)	Hz	14,08
S06	Middle trapezius	Left	Thoracic	Concave	Standing	Dynamic stiffness	N/m	280
S06	Middle trapezius	Left	Thoracic	Concave	Prone	Dynamic stiffness	N/m	264
S06	Middle trapezius	Left	Thoracic	Concave	Standing	Logarithmic decrement (elasticity)	dimensionless	0,94
S06	Middle trapezius	Left	Thoracic	Concave	Prone	Logarithmic decrement (elasticity)	dimensionless	1
S06	Middle trapezius	Left	Thoracic	Concave	Standing	Mechanical stress relaxation time	ms	17,3
S06	Middle trapezius	Left	Thoracic	Concave	Prone	Mechanical stress relaxation time	ms	18
S06	Middle trapezius	Left	Thoracic	Concave	Standing	Creep (Deborah number ratio)	dimensionless	1,04
S06	Middle trapezius	Left	Thoracic	Concave	Prone	Creep (Deborah number ratio)	dimensionless	1,06
S06	Middle trapezius	Right	Thoracic	Convex	Standing	Tone (oscillation frequency)	Hz	16,28
S06	Middle trapezius	Right	Thoracic	Convex	Prone	Tone (oscillation frequency)	Hz	14,26
S06	Middle trapezius	Right	Thoracic	Convex	Standing	Dynamic stiffness	N/m	330
S06	Middle trapezius	Right	Thoracic	Convex	Prone	Dynamic stiffness	N/m	260
S06	Middle trapezius	Right	Thoracic	Convex	Standing	Logarithmic decrement (elasticity)	dimensionless	1,03
S06	Middle trapezius	Right	Thoracic	Convex	Prone	Logarithmic decrement (elasticity)	dimensionless	1,05
S06	Middle trapezius	Right	Thoracic	Convex	Standing	Mechanical stress relaxation time	ms	15,2
S06	Middle trapezius	Right	Thoracic	Convex	Prone	Mechanical stress relaxation time	ms	17,8
S06	Middle trapezius	Right	Thoracic	Convex	Standing	Creep (Deborah number ratio)	dimensionless	0,93
S06	Middle trapezius	Right	Thoracic	Convex	Prone	Creep (Deborah number ratio)	dimensionless	1,04
S06	Lower trapezius	Left	Thoracic	Concave	Standing	Tone (oscillation frequency)	Hz	14,94
S06	Lower trapezius	Left	Thoracic	Concave	Prone	Tone (oscillation frequency)	Hz	17,42
S06	Lower trapezius	Left	Thoracic	Concave	Standing	Dynamic stiffness	N/m	331
S06	Lower trapezius	Left	Thoracic	Concave	Prone	Dynamic stiffness	N/m	418
S06	Lower trapezius	Left	Thoracic	Concave	Standing	Logarithmic decrement (elasticity)	dimensionless	1,25
S06	Lower trapezius	Left	Thoracic	Concave	Prone	Logarithmic decrement (elasticity)	dimensionless	0,88
S06	Lower trapezius	Left	Thoracic	Concave	Standing	Mechanical stress relaxation time	ms	15,5
S06	Lower trapezius	Left	Thoracic	Concave	Prone	Mechanical stress relaxation time	ms	12,5
S06	Lower trapezius	Left	Thoracic	Concave	Standing	Creep (Deborah number ratio)	dimensionless	0,96
S06	Lower trapezius	Left	Thoracic	Concave	Prone	Creep (Deborah number ratio)	dimensionless	0,78
S06	Lower trapezius	Right	Thoracic	Convex	Standing	Tone (oscillation frequency)	Hz	16,64
S06	Lower trapezius	Right	Thoracic	Convex	Prone	Tone (oscillation frequency)	Hz	15,52
S06	Lower trapezius	Right	Thoracic	Convex	Standing	Dynamic stiffness	N/m	398
S06	Lower trapezius	Right	Thoracic	Convex	Prone	Dynamic stiffness	N/m	291
S06	Lower trapezius	Right	Thoracic	Convex	Standing	Logarithmic decrement (elasticity)	dimensionless	1,15
S06	Lower trapezius	Right	Thoracic	Convex	Prone	Logarithmic decrement (elasticity)	dimensionless	1,03
S06	Lower trapezius	Right	Thoracic	Convex	Standing	Mechanical stress relaxation time	ms	13,5
S06	Lower trapezius	Right	Thoracic	Convex	Prone	Mechanical stress relaxation time	ms	16,6
S06	Lower trapezius	Right	Thoracic	Convex	Standing	Creep (Deborah number ratio)	dimensionless	0,84
S06	Lower trapezius	Right	Thoracic	Convex	Prone	Creep (Deborah number ratio)	dimensionless	0,99
S06	Latissimus dorsi	Left	Thoracic/thoracolumbar	Concave	Standing	Tone (oscillation frequency)	Hz	14,86
S06	Latissimus dorsi	Left	Thoracic/thoracolumbar	Concave	Prone	Tone (oscillation frequency)	Hz	19,42
S06	Latissimus dorsi	Left	Thoracic/thoracolumbar	Concave	Standing	Dynamic stiffness	N/m	327
S06	Latissimus dorsi	Left	Thoracic/thoracolumbar	Concave	Prone	Dynamic stiffness	N/m	439
S06	Latissimus dorsi	Left	Thoracic/thoracolumbar	Concave	Standing	Logarithmic decrement (elasticity)	dimensionless	1,27
S06	Latissimus dorsi	Left	Thoracic/thoracolumbar	Concave	Prone	Logarithmic decrement (elasticity)	dimensionless	0,84
S06	Latissimus dorsi	Left	Thoracic/thoracolumbar	Concave	Standing	Mechanical stress relaxation time	ms	16,9
S06	Latissimus dorsi	Left	Thoracic/thoracolumbar	Concave	Prone	Mechanical stress relaxation time	ms	12,1
S06	Latissimus dorsi	Left	Thoracic/thoracolumbar	Concave	Standing	Creep (Deborah number ratio)	dimensionless	1,06

S06	Latissimus dorsi	Left	Thoracic/thoracolumbar	Concave	Prone	Creep (Deborah number ratio)	dimensionless	0,76
S06	Latissimus dorsi	Right	Thoracic/thoracolumbar	Convex	Standing	Tone (oscillation frequency)	Hz	12,66
S06	Latissimus dorsi	Right	Thoracic/thoracolumbar	Convex	Prone	Tone (oscillation frequency)	Hz	17,24
S06	Latissimus dorsi	Right	Thoracic/thoracolumbar	Convex	Standing	Dynamic stiffness	N/m	294
S06	Latissimus dorsi	Right	Thoracic/thoracolumbar	Convex	Prone	Dynamic stiffness	N/m	375
S06	Latissimus dorsi	Right	Thoracic/thoracolumbar	Convex	Standing	Logarithmic decrement (elasticity)	dimensionless	1,14
S06	Latissimus dorsi	Right	Thoracic/thoracolumbar	Convex	Prone	Logarithmic decrement (elasticity)	dimensionless	0,97
S06	Latissimus dorsi	Right	Thoracic/thoracolumbar	Convex	Standing	Mechanical stress relaxation time	ms	16,3
S06	Latissimus dorsi	Right	Thoracic/thoracolumbar	Convex	Prone	Mechanical stress relaxation time	ms	13,5
S06	Latissimus dorsi	Right	Thoracic/thoracolumbar	Convex	Standing	Creep (Deborah number ratio)	dimensionless	0,96
S06	Latissimus dorsi	Right	Thoracic/thoracolumbar	Convex	Prone	Creep (Deborah number ratio)	dimensionless	0,83
S06	Lumbar erector spinae	Left	Lumbar	Convex	Standing	Tone (oscillation frequency)	Hz	13,78
S06	Lumbar erector spinae	Left	Lumbar	Convex	Prone	Tone (oscillation frequency)	Hz	18,46
S06	Lumbar erector spinae	Left	Lumbar	Convex	Standing	Dynamic stiffness	N/m	303
S06	Lumbar erector spinae	Left	Lumbar	Convex	Prone	Dynamic stiffness	N/m	463
S06	Lumbar erector spinae	Left	Lumbar	Convex	Standing	Logarithmic decrement (elasticity)	dimensionless	1,33
S06	Lumbar erector spinae	Left	Lumbar	Convex	Prone	Logarithmic decrement (elasticity)	dimensionless	1,33
S06	Lumbar erector spinae	Left	Lumbar	Convex	Standing	Mechanical stress relaxation time	ms	17,5
S06	Lumbar erector spinae	Left	Lumbar	Convex	Prone	Mechanical stress relaxation time	ms	12,2
S06	Lumbar erector spinae	Left	Lumbar	Convex	Standing	Creep (Deborah number ratio)	dimensionless	1,09
S06	Lumbar erector spinae	Left	Lumbar	Convex	Prone	Creep (Deborah number ratio)	dimensionless	0,79
S06	Lumbar erector spinae	Right	Lumbar	Concave	Standing	Tone (oscillation frequency)	Hz	11,94
S06	Lumbar erector spinae	Right	Lumbar	Concave	Prone	Tone (oscillation frequency)	Hz	14,84
S06	Lumbar erector spinae	Right	Lumbar	Concave	Standing	Dynamic stiffness	N/m	222
S06	Lumbar erector spinae	Right	Lumbar	Concave	Prone	Dynamic stiffness	N/m	305
S06	Lumbar erector spinae	Right	Lumbar	Concave	Standing	Logarithmic decrement (elasticity)	dimensionless	1,17
S06	Lumbar erector spinae	Right	Lumbar	Concave	Prone	Logarithmic decrement (elasticity)	dimensionless	1,05
S06	Lumbar erector spinae	Right	Lumbar	Concave	Standing	Mechanical stress relaxation time	ms	19,1
S06	Lumbar erector spinae	Right	Lumbar	Concave	Prone	Mechanical stress relaxation time	ms	16,8
S06	Lumbar erector spinae	Right	Lumbar	Concave	Standing	Creep (Deborah number ratio)	dimensionless	1,1
S06	Lumbar erector spinae	Right	Lumbar	Concave	Prone	Creep (Deborah number ratio)	dimensionless	1,02
S07	Middle trapezius	Left	Thoracic	Concave	Standing	Tone (oscillation frequency)	Hz	16,18
S07	Middle trapezius	Left	Thoracic	Concave	Prone	Tone (oscillation frequency)	Hz	15,66
S07	Middle trapezius	Left	Thoracic	Concave	Standing	Dynamic stiffness	N/m	342
S07	Middle trapezius	Left	Thoracic	Concave	Prone	Dynamic stiffness	N/m	317
S07	Middle trapezius	Left	Thoracic	Concave	Standing	Logarithmic decrement (elasticity)	dimensionless	0,99
S07	Middle trapezius	Left	Thoracic	Concave	Prone	Logarithmic decrement (elasticity)	dimensionless	1,3
S07	Middle trapezius	Left	Thoracic	Concave	Standing	Mechanical stress relaxation time	ms	15,9
S07	Middle trapezius	Left	Thoracic	Concave	Prone	Mechanical stress relaxation time	ms	15,6
S07	Middle trapezius	Left	Thoracic	Concave	Standing	Creep (Deborah number ratio)	dimensionless	0,98
S07	Middle trapezius	Left	Thoracic	Concave	Prone	Creep (Deborah number ratio)	dimensionless	0,96
S07	Middle trapezius	Right	Thoracic	Convex	Standing	Tone (oscillation frequency)	Hz	16,66
S07	Middle trapezius	Right	Thoracic	Convex	Prone	Tone (oscillation frequency)	Hz	15,76
S07	Middle trapezius	Right	Thoracic	Convex	Standing	Dynamic stiffness	N/m	323
S07	Middle trapezius	Right	Thoracic	Convex	Prone	Dynamic stiffness	N/m	327
S07	Middle trapezius	Right	Thoracic	Convex	Standing	Logarithmic decrement (elasticity)	dimensionless	0,76
S07	Middle trapezius	Right	Thoracic	Convex	Prone	Logarithmic decrement (elasticity)	dimensionless	1,04
S07	Middle trapezius	Right	Thoracic	Convex	Standing	Mechanical stress relaxation time	ms	15,4
S07	Middle trapezius	Right	Thoracic	Convex	Prone	Mechanical stress relaxation time	ms	15,6
S07	Middle trapezius	Right	Thoracic	Convex	Standing	Creep (Deborah number ratio)	dimensionless	0,94
S07	Middle trapezius	Right	Thoracic	Convex	Prone	Creep (Deborah number ratio)	dimensionless	0,95
S07	Lower trapezius	Left	Thoracic	Concave	Standing	Tone (oscillation frequency)	Hz	15,62
S07	Lower trapezius	Left	Thoracic	Concave	Prone	Tone (oscillation frequency)	Hz	17,84
S07	Lower trapezius	Left	Thoracic	Concave	Standing	Dynamic stiffness	N/m	324
S07	Lower trapezius	Left	Thoracic	Concave	Prone	Dynamic stiffness	N/m	380
S07	Lower trapezius	Left	Thoracic	Concave	Standing	Logarithmic decrement (elasticity)	dimensionless	1,17
S07	Lower trapezius	Left	Thoracic	Concave	Prone	Logarithmic decrement (elasticity)	dimensionless	1,02
S07	Lower trapezius	Left	Thoracic	Concave	Standing	Mechanical stress relaxation time	ms	15,3
S07	Lower trapezius	Left	Thoracic	Concave	Prone	Mechanical stress relaxation time	ms	13,4
S07	Lower trapezius	Left	Thoracic	Concave	Standing	Creep (Deborah number ratio)	dimensionless	0,93
S07	Lower trapezius	Left	Thoracic	Concave	Prone	Creep (Deborah number ratio)	dimensionless	0,83
S07	Lower trapezius	Right	Thoracic	Convex	Standing	Tone (oscillation frequency)	Hz	15,58
S07	Lower trapezius	Right	Thoracic	Convex	Prone	Tone (oscillation frequency)	Hz	17,98
S07	Lower trapezius	Right	Thoracic	Convex	Standing	Dynamic stiffness	N/m	302
S07	Lower trapezius	Right	Thoracic	Convex	Prone	Dynamic stiffness	N/m	389
S07	Lower trapezius	Right	Thoracic	Convex	Standing	Logarithmic decrement (elasticity)	dimensionless	0,99
S07	Lower trapezius	Right	Thoracic	Convex	Prone	Logarithmic decrement (elasticity)	dimensionless	1,03
S07	Lower trapezius	Right	Thoracic	Convex	Standing	Mechanical stress relaxation time	ms	16,4
S07	Lower trapezius	Right	Thoracic	Convex	Prone	Mechanical stress relaxation time	ms	13,1
S07	Lower trapezius	Right	Thoracic	Convex	Standing	Creep (Deborah number ratio)	dimensionless	1
S07	Lower trapezius	Right	Thoracic	Convex	Prone	Creep (Deborah number ratio)	dimensionless	0,81
S07	Latissimus dorsi	Left	Thoracic/thoracolumbar	Concave	Standing	Tone (oscillation frequency)	Hz	14,1
S07	Latissimus dorsi	Left	Thoracic/thoracolumbar	Concave	Prone	Tone (oscillation frequency)	Hz	15,94
S07	Latissimus dorsi	Left	Thoracic/thoracolumbar	Concave	Standing	Dynamic stiffness	N/m	279
S07	Latissimus dorsi	Left	Thoracic/thoracolumbar	Concave	Prone	Dynamic stiffness	N/m	319
S07	Latissimus dorsi	Left	Thoracic/thoracolumbar	Concave	Standing	Logarithmic decrement (elasticity)	dimensionless	1,17
S07	Latissimus dorsi	Left	Thoracic/thoracolumbar	Concave	Prone	Logarithmic decrement (elasticity)	dimensionless	0,95
S07	Latissimus dorsi	Left	Thoracic/thoracolumbar	Concave	Standing	Mechanical stress relaxation time	ms	17,3
S07	Latissimus dorsi	Left	Thoracic/thoracolumbar	Concave	Prone	Mechanical stress relaxation time	ms	16
S07	Latissimus dorsi	Left	Thoracic/thoracolumbar	Concave	Standing	Creep (Deborah number ratio)	dimensionless	1,03
S07	Latissimus dorsi	Left	Thoracic/thoracolumbar	Concave	Prone	Creep (Deborah number ratio)	dimensionless	0,98
S07	Latissimus dorsi	Right	Thoracic/thoracolumbar	Convex	Standing	Tone (oscillation frequency)	Hz	13,64
S07	Latissimus dorsi	Right	Thoracic/thoracolumbar	Convex	Prone	Tone (oscillation frequency)	Hz	16,78
S07	Latissimus dorsi	Right	Thoracic/thoracolumbar	Convex	Standing	Dynamic stiffness	N/m	275
S07	Latissimus dorsi	Right	Thoracic/thoracolumbar	Convex	Prone	Dynamic stiffness	N/m	392
S07	Latissimus dorsi	Right	Thoracic/thoracolumbar	Convex	Standing	Logarithmic decrement (elasticity)	dimensionless	1,15
S07	Latissimus dorsi	Right	Thoracic/thoracolumbar	Convex	Prone	Logarithmic decrement (elasticity)	dimensionless	0,92
S07	Latissimus dorsi	Right	Thoracic/thoracolumbar	Convex	Standing	Mechanical stress relaxation time	ms	17,5
S07	Latissimus dorsi	Right	Thoracic/thoracolumbar	Convex	Prone	Mechanical stress relaxation time	ms	13,2
S07	Latissimus dorsi	Right	Thoracic/thoracolumbar	Convex	Standing	Creep (Deborah number ratio)	dimensionless	1,04

S07	Latissimus dorsi	Right	Thoracic/thoracolumbar	Convex	Prone	Creep (Deborah number ratio)	dimensionless	0,8
S07	Lumbar erector spinae	Left	Lumbar	Convex	Standing	Tone (oscillation frequency)	Hz	10,86
S07	Lumbar erector spinae	Left	Lumbar	Convex	Prone	Tone (oscillation frequency)	Hz	12,16
S07	Lumbar erector spinae	Left	Lumbar	Convex	Standing	Dynamic stiffness	N/m	145
S07	Lumbar erector spinae	Left	Lumbar	Convex	Prone	Dynamic stiffness	N/m	215
S07	Lumbar erector spinae	Left	Lumbar	Convex	Standing	Logarithmic decrement (elasticity)	dimensionless	1,04
S07	Lumbar erector spinae	Left	Lumbar	Convex	Prone	Logarithmic decrement (elasticity)	dimensionless	1,03
S07	Lumbar erector spinae	Left	Lumbar	Convex	Standing	Mechanical stress relaxation time	ms	22,9
S07	Lumbar erector spinae	Left	Lumbar	Convex	Prone	Mechanical stress relaxation time	ms	19,1
S07	Lumbar erector spinae	Left	Lumbar	Convex	Standing	Creep (Deborah number ratio)	dimensionless	1,22
S07	Lumbar erector spinae	Left	Lumbar	Convex	Prone	Creep (Deborah number ratio)	dimensionless	1,06
S07	Lumbar erector spinae	Right	Lumbar	Concave	Standing	Tone (oscillation frequency)	Hz	10,58
S07	Lumbar erector spinae	Right	Lumbar	Concave	Prone	Tone (oscillation frequency)	Hz	12,9
S07	Lumbar erector spinae	Right	Lumbar	Concave	Standing	Dynamic stiffness	N/m	140
S07	Lumbar erector spinae	Right	Lumbar	Concave	Prone	Dynamic stiffness	N/m	248
S07	Lumbar erector spinae	Right	Lumbar	Concave	Standing	Logarithmic decrement (elasticity)	dimensionless	0,96
S07	Lumbar erector spinae	Right	Lumbar	Concave	Prone	Logarithmic decrement (elasticity)	dimensionless	0,98
S07	Lumbar erector spinae	Right	Lumbar	Concave	Standing	Mechanical stress relaxation time	ms	25
S07	Lumbar erector spinae	Right	Lumbar	Concave	Prone	Mechanical stress relaxation time	ms	19,1
S07	Lumbar erector spinae	Right	Lumbar	Concave	Standing	Creep (Deborah number ratio)	dimensionless	1,35
S07	Lumbar erector spinae	Right	Lumbar	Concave	Prone	Creep (Deborah number ratio)	dimensionless	1,09
S08	Middle trapezius	Left	Thoracic	Concave	Standing	Tone (oscillation frequency)	Hz	13,58
S08	Middle trapezius	Left	Thoracic	Concave	Prone	Tone (oscillation frequency)	Hz	15,52
S08	Middle trapezius	Left	Thoracic	Concave	Standing	Dynamic stiffness	N/m	235
S08	Middle trapezius	Left	Thoracic	Concave	Prone	Dynamic stiffness	N/m	309
S08	Middle trapezius	Left	Thoracic	Concave	Standing	Logarithmic decrement (elasticity)	dimensionless	1,06
S08	Middle trapezius	Left	Thoracic	Concave	Prone	Logarithmic decrement (elasticity)	dimensionless	1,11
S08	Middle trapezius	Left	Thoracic	Concave	Standing	Mechanical stress relaxation time	ms	18,1
S08	Middle trapezius	Left	Thoracic	Concave	Prone	Mechanical stress relaxation time	ms	15,4
S08	Middle trapezius	Left	Thoracic	Concave	Standing	Creep (Deborah number ratio)	dimensionless	1,01
S08	Middle trapezius	Left	Thoracic	Concave	Prone	Creep (Deborah number ratio)	dimensionless	0,92
S08	Middle trapezius	Right	Thoracic	Convex	Standing	Tone (oscillation frequency)	Hz	17,23
S08	Middle trapezius	Right	Thoracic	Convex	Prone	Tone (oscillation frequency)	Hz	15,68
S08	Middle trapezius	Right	Thoracic	Convex	Standing	Dynamic stiffness	N/m	376
S08	Middle trapezius	Right	Thoracic	Convex	Prone	Dynamic stiffness	N/m	290
S08	Middle trapezius	Right	Thoracic	Convex	Standing	Logarithmic decrement (elasticity)	dimensionless	1,46
S08	Middle trapezius	Right	Thoracic	Convex	Prone	Logarithmic decrement (elasticity)	dimensionless	1,22
S08	Middle trapezius	Right	Thoracic	Convex	Standing	Mechanical stress relaxation time	ms	14,8
S08	Middle trapezius	Right	Thoracic	Convex	Prone	Mechanical stress relaxation time	ms	16,8
S08	Middle trapezius	Right	Thoracic	Convex	Standing	Creep (Deborah number ratio)	dimensionless	0,93
S08	Middle trapezius	Right	Thoracic	Convex	Prone	Creep (Deborah number ratio)	dimensionless	1,02
S08	Lower trapezius	Left	Thoracic	Concave	Standing	Tone (oscillation frequency)	Hz	18
S08	Lower trapezius	Left	Thoracic	Concave	Prone	Tone (oscillation frequency)	Hz	19,36
S08	Lower trapezius	Left	Thoracic	Concave	Standing	Dynamic stiffness	N/m	555
S08	Lower trapezius	Left	Thoracic	Concave	Prone	Dynamic stiffness	N/m	466
S08	Lower trapezius	Left	Thoracic	Concave	Standing	Logarithmic decrement (elasticity)	dimensionless	1,84
S08	Lower trapezius	Left	Thoracic	Concave	Prone	Logarithmic decrement (elasticity)	dimensionless	0,88
S08	Lower trapezius	Left	Thoracic	Concave	Standing	Mechanical stress relaxation time	ms	11,2
S08	Lower trapezius	Left	Thoracic	Concave	Prone	Mechanical stress relaxation time	ms	11,6
S08	Lower trapezius	Left	Thoracic	Concave	Standing	Creep (Deborah number ratio)	dimensionless	0,74
S08	Lower trapezius	Left	Thoracic	Concave	Prone	Creep (Deborah number ratio)	dimensionless	0,72
S08	Lower trapezius	Right	Thoracic	Convex	Standing	Tone (oscillation frequency)	Hz	16,86
S08	Lower trapezius	Right	Thoracic	Convex	Prone	Tone (oscillation frequency)	Hz	15,7
S08	Lower trapezius	Right	Thoracic	Convex	Standing	Dynamic stiffness	N/m	386
S08	Lower trapezius	Right	Thoracic	Convex	Prone	Dynamic stiffness	N/m	311
S08	Lower trapezius	Right	Thoracic	Convex	Standing	Logarithmic decrement (elasticity)	dimensionless	1,24
S08	Lower trapezius	Right	Thoracic	Convex	Prone	Logarithmic decrement (elasticity)	dimensionless	1,19
S08	Lower trapezius	Right	Thoracic	Convex	Standing	Mechanical stress relaxation time	ms	13,7
S08	Lower trapezius	Right	Thoracic	Convex	Prone	Mechanical stress relaxation time	ms	16,4
S08	Lower trapezius	Right	Thoracic	Convex	Standing	Creep (Deborah number ratio)	dimensionless	0,85
S08	Lower trapezius	Right	Thoracic	Convex	Prone	Creep (Deborah number ratio)	dimensionless	1,01
S08	Latissimus dorsi	Left	Thoracic/thoracolumbar	Concave	Standing	Tone (oscillation frequency)	Hz	10,62
S08	Latissimus dorsi	Left	Thoracic/thoracolumbar	Concave	Prone	Tone (oscillation frequency)	Hz	17,5
S08	Latissimus dorsi	Left	Thoracic/thoracolumbar	Concave	Standing	Dynamic stiffness	N/m	197
S08	Latissimus dorsi	Left	Thoracic/thoracolumbar	Concave	Prone	Dynamic stiffness	N/m	379
S08	Latissimus dorsi	Left	Thoracic/thoracolumbar	Concave	Standing	Logarithmic decrement (elasticity)	dimensionless	1,59
S08	Latissimus dorsi	Left	Thoracic/thoracolumbar	Concave	Prone	Logarithmic decrement (elasticity)	dimensionless	1,01
S08	Latissimus dorsi	Left	Thoracic/thoracolumbar	Concave	Standing	Mechanical stress relaxation time	ms	23,6
S08	Latissimus dorsi	Left	Thoracic/thoracolumbar	Concave	Prone	Mechanical stress relaxation time	ms	14,1
S08	Latissimus dorsi	Left	Thoracic/thoracolumbar	Concave	Standing	Creep (Deborah number ratio)	dimensionless	1,41
S08	Latissimus dorsi	Left	Thoracic/thoracolumbar	Concave	Prone	Creep (Deborah number ratio)	dimensionless	0,88
S08	Latissimus dorsi	Right	Thoracic/thoracolumbar	Convex	Standing	Tone (oscillation frequency)	Hz	13,44
S08	Latissimus dorsi	Right	Thoracic/thoracolumbar	Convex	Prone	Tone (oscillation frequency)	Hz	16,34
S08	Latissimus dorsi	Right	Thoracic/thoracolumbar	Convex	Standing	Dynamic stiffness	N/m	251
S08	Latissimus dorsi	Right	Thoracic/thoracolumbar	Convex	Prone	Dynamic stiffness	N/m	339
S08	Latissimus dorsi	Right	Thoracic/thoracolumbar	Convex	Standing	Logarithmic decrement (elasticity)	dimensionless	0,98
S08	Latissimus dorsi	Right	Thoracic/thoracolumbar	Convex	Prone	Logarithmic decrement (elasticity)	dimensionless	0,98
S08	Latissimus dorsi	Right	Thoracic/thoracolumbar	Convex	Standing	Mechanical stress relaxation time	ms	19
S08	Latissimus dorsi	Right	Thoracic/thoracolumbar	Convex	Prone	Mechanical stress relaxation time	ms	15,7
S08	Latissimus dorsi	Right	Thoracic/thoracolumbar	Convex	Standing	Creep (Deborah number ratio)	dimensionless	1,15
S08	Latissimus dorsi	Right	Thoracic/thoracolumbar	Convex	Prone	Creep (Deborah number ratio)	dimensionless	0,97
S08	Lumbar erector spinae	Left	Lumbar	Convex	Standing	Tone (oscillation frequency)	Hz	12,34
S08	Lumbar erector spinae	Left	Lumbar	Convex	Prone	Tone (oscillation frequency)	Hz	13,74
S08	Lumbar erector spinae	Left	Lumbar	Convex	Standing	Dynamic stiffness	N/m	221
S08	Lumbar erector spinae	Left	Lumbar	Convex	Prone	Dynamic stiffness	N/m	300
S08	Lumbar erector spinae	Left	Lumbar	Convex	Standing	Logarithmic decrement (elasticity)	dimensionless	1,05
S08	Lumbar erector spinae	Left	Lumbar	Convex	Prone	Logarithmic decrement (elasticity)	dimensionless	1,11
S08	Lumbar erector spinae	Left	Lumbar	Convex	Standing	Mechanical stress relaxation time	ms	21,5
S08	Lumbar erector spinae	Left	Lumbar	Convex	Prone	Mechanical stress relaxation time	ms	17
S08	Lumbar erector spinae	Left	Lumbar	Convex	Standing	Creep (Deborah number ratio)	dimensionless	1,29

S08	Lumbar erector spinae	Left	Lumbar	Convex	Prone	Creep (Deborah number ratio)	dimensionless	1,03
S08	Lumbar erector spinae	Right	Lumbar	Concave	Standing	Tone (oscillation frequency)	Hz	13,04
S08	Lumbar erector spinae	Right	Lumbar	Concave	Prone	Tone (oscillation frequency)	Hz	12,46
S08	Lumbar erector spinae	Right	Lumbar	Concave	Standing	Dynamic stiffness	N/m	239
S08	Lumbar erector spinae	Right	Lumbar	Concave	Prone	Dynamic stiffness	N/m	193
S08	Lumbar erector spinae	Right	Lumbar	Concave	Standing	Logarithmic decrement (elasticity)	dimensionless	0,96
S08	Lumbar erector spinae	Right	Lumbar	Concave	Prone	Logarithmic decrement (elasticity)	dimensionless	0,89
S08	Lumbar erector spinae	Right	Lumbar	Concave	Standing	Mechanical stress relaxation time	ms	18,2
S08	Lumbar erector spinae	Right	Lumbar	Concave	Prone	Mechanical stress relaxation time	ms	22
S08	Lumbar erector spinae	Right	Lumbar	Concave	Standing	Creep (Deborah number ratio)	dimensionless	1,02
S08	Lumbar erector spinae	Right	Lumbar	Concave	Prone	Creep (Deborah number ratio)	dimensionless	1,23
S09	Middle trapezius	Left	Thoracic	Concave	Standing	Tone (oscillation frequency)	Hz	15,22
S09	Middle trapezius	Left	Thoracic	Concave	Prone	Tone (oscillation frequency)	Hz	13,44
S09	Middle trapezius	Left	Thoracic	Concave	Standing	Dynamic stiffness	N/m	305
S09	Middle trapezius	Left	Thoracic	Concave	Prone	Dynamic stiffness	N/m	238
S09	Middle trapezius	Left	Thoracic	Concave	Standing	Logarithmic decrement (elasticity)	dimensionless	1,14
S09	Middle trapezius	Left	Thoracic	Concave	Prone	Logarithmic decrement (elasticity)	dimensionless	1,03
S09	Middle trapezius	Left	Thoracic	Concave	Standing	Mechanical stress relaxation time	ms	16,2
S09	Middle trapezius	Left	Thoracic	Concave	Prone	Mechanical stress relaxation time	ms	18,5
S09	Middle trapezius	Left	Thoracic	Concave	Standing	Creep (Deborah number ratio)	dimensionless	0,98
S09	Middle trapezius	Left	Thoracic	Concave	Prone	Creep (Deborah number ratio)	dimensionless	1,07
S09	Middle trapezius	Right	Thoracic	Convex	Standing	Tone (oscillation frequency)	Hz	14,18
S09	Middle trapezius	Right	Thoracic	Convex	Prone	Tone (oscillation frequency)	Hz	12,32
S09	Middle trapezius	Right	Thoracic	Convex	Standing	Dynamic stiffness	N/m	246
S09	Middle trapezius	Right	Thoracic	Convex	Prone	Dynamic stiffness	N/m	185
S09	Middle trapezius	Right	Thoracic	Convex	Standing	Logarithmic decrement (elasticity)	dimensionless	0,95
S09	Middle trapezius	Right	Thoracic	Convex	Prone	Logarithmic decrement (elasticity)	dimensionless	1,03
S09	Middle trapezius	Right	Thoracic	Convex	Standing	Mechanical stress relaxation time	ms	18,4
S09	Middle trapezius	Right	Thoracic	Convex	Prone	Mechanical stress relaxation time	ms	20,5
S09	Middle trapezius	Right	Thoracic	Convex	Standing	Creep (Deborah number ratio)	dimensionless	1,08
S09	Middle trapezius	Right	Thoracic	Convex	Prone	Creep (Deborah number ratio)	dimensionless	1,15
S09	Lower trapezius	Left	Thoracic	Concave	Standing	Tone (oscillation frequency)	Hz	13,86
S09	Lower trapezius	Left	Thoracic	Concave	Prone	Tone (oscillation frequency)	Hz	15,22
S09	Lower trapezius	Left	Thoracic	Concave	Standing	Dynamic stiffness	N/m	277
S09	Lower trapezius	Left	Thoracic	Concave	Prone	Dynamic stiffness	N/m	301
S09	Lower trapezius	Left	Thoracic	Concave	Standing	Logarithmic decrement (elasticity)	dimensionless	1,28
S09	Lower trapezius	Left	Thoracic	Concave	Prone	Logarithmic decrement (elasticity)	dimensionless	1,16
S09	Lower trapezius	Left	Thoracic	Concave	Standing	Mechanical stress relaxation time	ms	18
S09	Lower trapezius	Left	Thoracic	Concave	Prone	Mechanical stress relaxation time	ms	15,9
S09	Lower trapezius	Left	Thoracic	Concave	Standing	Creep (Deborah number ratio)	dimensionless	1,09
S09	Lower trapezius	Left	Thoracic	Concave	Prone	Creep (Deborah number ratio)	dimensionless	0,93
S09	Lower trapezius	Right	Thoracic	Convex	Standing	Tone (oscillation frequency)	Hz	14,06
S09	Lower trapezius	Right	Thoracic	Convex	Prone	Tone (oscillation frequency)	Hz	14,2
S09	Lower trapezius	Right	Thoracic	Convex	Standing	Dynamic stiffness	N/m	258
S09	Lower trapezius	Right	Thoracic	Convex	Prone	Dynamic stiffness	N/m	246
S09	Lower trapezius	Right	Thoracic	Convex	Standing	Logarithmic decrement (elasticity)	dimensionless	1,15
S09	Lower trapezius	Right	Thoracic	Convex	Prone	Logarithmic decrement (elasticity)	dimensionless	1,13
S09	Lower trapezius	Right	Thoracic	Convex	Standing	Mechanical stress relaxation time	ms	17,9
S09	Lower trapezius	Right	Thoracic	Convex	Prone	Mechanical stress relaxation time	ms	17,9
S09	Lower trapezius	Right	Thoracic	Convex	Standing	Creep (Deborah number ratio)	dimensionless	1,08
S09	Lower trapezius	Right	Thoracic	Convex	Prone	Creep (Deborah number ratio)	dimensionless	1,04
S09	Latissimus dorsi	Left	Thoracic/thoracolumbar	Concave	Standing	Tone (oscillation frequency)	Hz	12,38
S09	Latissimus dorsi	Left	Thoracic/thoracolumbar	Concave	Prone	Tone (oscillation frequency)	Hz	15,22
S09	Latissimus dorsi	Left	Thoracic/thoracolumbar	Concave	Standing	Dynamic stiffness	N/m	230
S09	Latissimus dorsi	Left	Thoracic/thoracolumbar	Concave	Prone	Dynamic stiffness	N/m	301
S09	Latissimus dorsi	Left	Thoracic/thoracolumbar	Concave	Standing	Logarithmic decrement (elasticity)	dimensionless	1,11
S09	Latissimus dorsi	Left	Thoracic/thoracolumbar	Concave	Prone	Logarithmic decrement (elasticity)	dimensionless	1,16
S09	Latissimus dorsi	Left	Thoracic/thoracolumbar	Concave	Standing	Mechanical stress relaxation time	ms	19,7
S09	Latissimus dorsi	Left	Thoracic/thoracolumbar	Concave	Prone	Mechanical stress relaxation time	ms	15,9
S09	Latissimus dorsi	Left	Thoracic/thoracolumbar	Concave	Standing	Creep (Deborah number ratio)	dimensionless	1,17
S09	Latissimus dorsi	Left	Thoracic/thoracolumbar	Concave	Prone	Creep (Deborah number ratio)	dimensionless	0,93
S09	Latissimus dorsi	Right	Thoracic/thoracolumbar	Convex	Standing	Tone (oscillation frequency)	Hz	14,58
S09	Latissimus dorsi	Right	Thoracic/thoracolumbar	Convex	Prone	Tone (oscillation frequency)	Hz	15,94
S09	Latissimus dorsi	Right	Thoracic/thoracolumbar	Convex	Standing	Dynamic stiffness	N/m	359
S09	Latissimus dorsi	Right	Thoracic/thoracolumbar	Convex	Prone	Dynamic stiffness	N/m	305
S09	Latissimus dorsi	Right	Thoracic/thoracolumbar	Convex	Standing	Logarithmic decrement (elasticity)	dimensionless	1,13
S09	Latissimus dorsi	Right	Thoracic/thoracolumbar	Convex	Prone	Logarithmic decrement (elasticity)	dimensionless	0,85
S09	Latissimus dorsi	Right	Thoracic/thoracolumbar	Convex	Standing	Mechanical stress relaxation time	ms	14
S09	Latissimus dorsi	Right	Thoracic/thoracolumbar	Convex	Prone	Mechanical stress relaxation time	ms	16,4
S09	Latissimus dorsi	Right	Thoracic/thoracolumbar	Convex	Standing	Creep (Deborah number ratio)	dimensionless	0,84
S09	Latissimus dorsi	Right	Thoracic/thoracolumbar	Convex	Prone	Creep (Deborah number ratio)	dimensionless	0,99
S09	Lumbar erector spinae	Left	Lumbar	Convex	Standing	Tone (oscillation frequency)	Hz	12,44
S09	Lumbar erector spinae	Left	Lumbar	Convex	Prone	Tone (oscillation frequency)	Hz	12,98
S09	Lumbar erector spinae	Left	Lumbar	Convex	Standing	Dynamic stiffness	N/m	198
S09	Lumbar erector spinae	Left	Lumbar	Convex	Prone	Dynamic stiffness	N/m	221
S09	Lumbar erector spinae	Left	Lumbar	Convex	Standing	Logarithmic decrement (elasticity)	dimensionless	0,87
S09	Lumbar erector spinae	Left	Lumbar	Convex	Prone	Logarithmic decrement (elasticity)	dimensionless	0,97
S09	Lumbar erector spinae	Left	Lumbar	Convex	Standing	Mechanical stress relaxation time	ms	19,6
S09	Lumbar erector spinae	Left	Lumbar	Convex	Prone	Mechanical stress relaxation time	ms	18,7
S09	Lumbar erector spinae	Left	Lumbar	Convex	Standing	Creep (Deborah number ratio)	dimensionless	1,04
S09	Lumbar erector spinae	Left	Lumbar	Convex	Prone	Creep (Deborah number ratio)	dimensionless	1,02
S09	Lumbar erector spinae	Right	Lumbar	Concave	Standing	Tone (oscillation frequency)	Hz	12,74
S09	Lumbar erector spinae	Right	Lumbar	Concave	Prone	Tone (oscillation frequency)	Hz	12,78
S09	Lumbar erector spinae	Right	Lumbar	Concave	Standing	Dynamic stiffness	N/m	230
S09	Lumbar erector spinae	Right	Lumbar	Concave	Prone	Dynamic stiffness	N/m	181
S09	Lumbar erector spinae	Right	Lumbar	Concave	Standing	Logarithmic decrement (elasticity)	dimensionless	0,84
S09	Lumbar erector spinae	Right	Lumbar	Concave	Prone	Logarithmic decrement (elasticity)	dimensionless	0,77
S09	Lumbar erector spinae	Right	Lumbar	Concave	Standing	Mechanical stress relaxation time	ms	18,2
S09	Lumbar erector spinae	Right	Lumbar	Concave	Prone	Mechanical stress relaxation time	ms	20,1
S09	Lumbar erector spinae	Right	Lumbar	Concave	Standing	Creep (Deborah number ratio)	dimensionless	0,97

S09	Lumbar erector spinae	Right	Lumbar	Concave	Prone	Creep (Deborah number ratio)	dimensionless	1,04
S10	Middle trapezius	Left	Thoracic	Concave	Standing	Tone (oscillation frequency)	Hz	15,12
S10	Middle trapezius	Left	Thoracic	Concave	Prone	Tone (oscillation frequency)	Hz	14,82
S10	Middle trapezius	Left	Thoracic	Concave	Standing	Dynamic stiffness	N/m	306
S10	Middle trapezius	Left	Thoracic	Concave	Prone	Dynamic stiffness	N/m	292
S10	Middle trapezius	Left	Thoracic	Concave	Standing	Logarithmic decrement (elasticity)	dimensionless	1,08
S10	Middle trapezius	Left	Thoracic	Concave	Prone	Logarithmic decrement (elasticity)	dimensionless	1,11
S10	Middle trapezius	Left	Thoracic	Concave	Standing	Mechanical stress relaxation time	ms	16,3
S10	Middle trapezius	Left	Thoracic	Concave	Prone	Mechanical stress relaxation time	ms	16,3
S10	Middle trapezius	Left	Thoracic	Concave	Standing	Creep (Deborah number ratio)	dimensionless	0,99
S10	Middle trapezius	Left	Thoracic	Concave	Prone	Creep (Deborah number ratio)	dimensionless	0,97
S10	Middle trapezius	Right	Thoracic	Convex	Standing	Tone (oscillation frequency)	Hz	15,18
S10	Middle trapezius	Right	Thoracic	Convex	Prone	Tone (oscillation frequency)	Hz	14,92
S10	Middle trapezius	Right	Thoracic	Convex	Standing	Dynamic stiffness	N/m	297
S10	Middle trapezius	Right	Thoracic	Convex	Prone	Dynamic stiffness	N/m	276
S10	Middle trapezius	Right	Thoracic	Convex	Standing	Logarithmic decrement (elasticity)	dimensionless	0,92
S10	Middle trapezius	Right	Thoracic	Convex	Prone	Logarithmic decrement (elasticity)	dimensionless	1,08
S10	Middle trapezius	Right	Thoracic	Convex	Standing	Mechanical stress relaxation time	ms	16,9
S10	Middle trapezius	Right	Thoracic	Convex	Prone	Mechanical stress relaxation time	ms	17,2
S10	Middle trapezius	Right	Thoracic	Convex	Standing	Creep (Deborah number ratio)	dimensionless	1,03
S10	Middle trapezius	Right	Thoracic	Convex	Prone	Creep (Deborah number ratio)	dimensionless	1,02
S10	Lower trapezius	Left	Thoracic	Concave	Standing	Tone (oscillation frequency)	Hz	13,82
S10	Lower trapezius	Left	Thoracic	Concave	Prone	Tone (oscillation frequency)	Hz	14,32
S10	Lower trapezius	Left	Thoracic	Concave	Standing	Dynamic stiffness	N/m	265
S10	Lower trapezius	Left	Thoracic	Concave	Prone	Dynamic stiffness	N/m	280
S10	Lower trapezius	Left	Thoracic	Concave	Standing	Logarithmic decrement (elasticity)	dimensionless	1
S10	Lower trapezius	Left	Thoracic	Concave	Prone	Logarithmic decrement (elasticity)	dimensionless	1,07
S10	Lower trapezius	Left	Thoracic	Concave	Standing	Mechanical stress relaxation time	ms	18,7
S10	Lower trapezius	Left	Thoracic	Concave	Prone	Mechanical stress relaxation time	ms	17,5
S10	Lower trapezius	Left	Thoracic	Concave	Standing	Creep (Deborah number ratio)	dimensionless	1,14
S10	Lower trapezius	Left	Thoracic	Concave	Prone	Creep (Deborah number ratio)	dimensionless	1,04
S10	Lower trapezius	Right	Thoracic	Convex	Standing	Tone (oscillation frequency)	Hz	15,98
S10	Lower trapezius	Right	Thoracic	Convex	Prone	Tone (oscillation frequency)	Hz	13,74
S10	Lower trapezius	Right	Thoracic	Convex	Standing	Dynamic stiffness	N/m	363
S10	Lower trapezius	Right	Thoracic	Convex	Prone	Dynamic stiffness	N/m	245
S10	Lower trapezius	Right	Thoracic	Convex	Standing	Logarithmic decrement (elasticity)	dimensionless	0,87
S10	Lower trapezius	Right	Thoracic	Convex	Prone	Logarithmic decrement (elasticity)	dimensionless	0,97
S10	Lower trapezius	Right	Thoracic	Convex	Standing	Mechanical stress relaxation time	ms	13,8
S10	Lower trapezius	Right	Thoracic	Convex	Prone	Mechanical stress relaxation time	ms	18,9
S10	Lower trapezius	Right	Thoracic	Convex	Standing	Creep (Deborah number ratio)	dimensionless	0,85
S10	Lower trapezius	Right	Thoracic	Convex	Prone	Creep (Deborah number ratio)	dimensionless	1,1
S10	Latissimus dorsi	Left	Thoracic/thoracolumbar	Concave	Standing	Tone (oscillation frequency)	Hz	12,3
S10	Latissimus dorsi	Left	Thoracic/thoracolumbar	Concave	Prone	Tone (oscillation frequency)	Hz	14,9
S10	Latissimus dorsi	Left	Thoracic/thoracolumbar	Concave	Standing	Dynamic stiffness	N/m	259
S10	Latissimus dorsi	Left	Thoracic/thoracolumbar	Concave	Prone	Dynamic stiffness	N/m	319
S10	Latissimus dorsi	Left	Thoracic/thoracolumbar	Concave	Standing	Logarithmic decrement (elasticity)	dimensionless	1,3
S10	Latissimus dorsi	Left	Thoracic/thoracolumbar	Concave	Prone	Logarithmic decrement (elasticity)	dimensionless	0,97
S10	Latissimus dorsi	Left	Thoracic/thoracolumbar	Concave	Standing	Mechanical stress relaxation time	ms	17,8
S10	Latissimus dorsi	Left	Thoracic/thoracolumbar	Concave	Prone	Mechanical stress relaxation time	ms	15,8
S10	Latissimus dorsi	Left	Thoracic/thoracolumbar	Concave	Standing	Creep (Deborah number ratio)	dimensionless	1,05
S10	Latissimus dorsi	Left	Thoracic/thoracolumbar	Concave	Prone	Creep (Deborah number ratio)	dimensionless	0,96
S10	Latissimus dorsi	Right	Thoracic/thoracolumbar	Convex	Standing	Tone (oscillation frequency)	Hz	11,94
S10	Latissimus dorsi	Right	Thoracic/thoracolumbar	Convex	Prone	Tone (oscillation frequency)	Hz	13,46
S10	Latissimus dorsi	Right	Thoracic/thoracolumbar	Convex	Standing	Dynamic stiffness	N/m	215
S10	Latissimus dorsi	Right	Thoracic/thoracolumbar	Convex	Prone	Dynamic stiffness	N/m	227
S10	Latissimus dorsi	Right	Thoracic/thoracolumbar	Convex	Standing	Logarithmic decrement (elasticity)	dimensionless	1,1
S10	Latissimus dorsi	Right	Thoracic/thoracolumbar	Convex	Prone	Logarithmic decrement (elasticity)	dimensionless	0,9
S10	Latissimus dorsi	Right	Thoracic/thoracolumbar	Convex	Standing	Mechanical stress relaxation time	ms	19,4
S10	Latissimus dorsi	Right	Thoracic/thoracolumbar	Convex	Prone	Mechanical stress relaxation time	ms	18,5
S10	Latissimus dorsi	Right	Thoracic/thoracolumbar	Convex	Standing	Creep (Deborah number ratio)	dimensionless	1,07
S10	Latissimus dorsi	Right	Thoracic/thoracolumbar	Convex	Prone	Creep (Deborah number ratio)	dimensionless	0,99
S10	Lumbar erector spinae	Left	Lumbar	Convex	Standing	Tone (oscillation frequency)	Hz	12,38
S10	Lumbar erector spinae	Left	Lumbar	Convex	Prone	Tone (oscillation frequency)	Hz	13,34
S10	Lumbar erector spinae	Left	Lumbar	Convex	Standing	Dynamic stiffness	N/m	228
S10	Lumbar erector spinae	Left	Lumbar	Convex	Prone	Dynamic stiffness	N/m	269
S10	Lumbar erector spinae	Left	Lumbar	Convex	Standing	Logarithmic decrement (elasticity)	dimensionless	0,91
S10	Lumbar erector spinae	Left	Lumbar	Convex	Prone	Logarithmic decrement (elasticity)	dimensionless	1,35
S10	Lumbar erector spinae	Left	Lumbar	Convex	Standing	Mechanical stress relaxation time	ms	18,8
S10	Lumbar erector spinae	Left	Lumbar	Convex	Prone	Mechanical stress relaxation time	ms	17,9
S10	Lumbar erector spinae	Left	Lumbar	Convex	Standing	Creep (Deborah number ratio)	dimensionless	1,05
S10	Lumbar erector spinae	Left	Lumbar	Convex	Prone	Creep (Deborah number ratio)	dimensionless	1,06
S10	Lumbar erector spinae	Right	Lumbar	Concave	Standing	Tone (oscillation frequency)	Hz	12,36
S10	Lumbar erector spinae	Right	Lumbar	Concave	Prone	Tone (oscillation frequency)	Hz	12,4
S10	Lumbar erector spinae	Right	Lumbar	Concave	Standing	Dynamic stiffness	N/m	228
S10	Lumbar erector spinae	Right	Lumbar	Concave	Prone	Dynamic stiffness	N/m	227
S10	Lumbar erector spinae	Right	Lumbar	Concave	Standing	Logarithmic decrement (elasticity)	dimensionless	0,94
S10	Lumbar erector spinae	Right	Lumbar	Concave	Prone	Logarithmic decrement (elasticity)	dimensionless	1,1
S10	Lumbar erector spinae	Right	Lumbar	Concave	Standing	Mechanical stress relaxation time	ms	18,9
S10	Lumbar erector spinae	Right	Lumbar	Concave	Prone	Mechanical stress relaxation time	ms	20,1
S10	Lumbar erector spinae	Right	Lumbar	Concave	Standing	Creep (Deborah number ratio)	dimensionless	1,04
S10	Lumbar erector spinae	Right	Lumbar	Concave	Prone	Creep (Deborah number ratio)	dimensionless	1,16
S11	Middle trapezius	Left	Thoracic	Concave	Standing	Tone (oscillation frequency)	Hz	17,8
S11	Middle trapezius	Left	Thoracic	Concave	Prone	Tone (oscillation frequency)	Hz	15,54
S11	Middle trapezius	Left	Thoracic	Concave	Standing	Dynamic stiffness	N/m	381
S11	Middle trapezius	Left	Thoracic	Concave	Prone	Dynamic stiffness	N/m	295
S11	Middle trapezius	Left	Thoracic	Concave	Standing	Logarithmic decrement (elasticity)	dimensionless	0,77
S11	Middle trapezius	Left	Thoracic	Concave	Prone	Logarithmic decrement (elasticity)	dimensionless	1
S11	Middle trapezius	Left	Thoracic	Concave	Standing	Mechanical stress relaxation time	ms	13,4
S11	Middle trapezius	Left	Thoracic	Concave	Prone	Mechanical stress relaxation time	ms	17,4
S11	Middle trapezius	Left	Thoracic	Concave	Standing	Creep (Deborah number ratio)	dimensionless	0,82

S11	Middle trapezius	Left	Thoracic	Concave	Prone	Creep (Deborah number ratio)	dimensionless	1,05
S11	Middle trapezius	Right	Thoracic	Convex	Standing	Tone (oscillation frequency)	Hz	15,94
S11	Middle trapezius	Right	Thoracic	Convex	Prone	Tone (oscillation frequency)	Hz	14,42
S11	Middle trapezius	Right	Thoracic	Convex	Standing	Dynamic stiffness	N/m	287
S11	Middle trapezius	Right	Thoracic	Convex	Prone	Dynamic stiffness	N/m	261
S11	Middle trapezius	Right	Thoracic	Convex	Standing	Logarithmic decrement (elasticity)	dimensionless	0,83
S11	Middle trapezius	Right	Thoracic	Convex	Prone	Logarithmic decrement (elasticity)	dimensionless	1,08
S11	Middle trapezius	Right	Thoracic	Convex	Standing	Mechanical stress relaxation time	ms	16,9
S11	Middle trapezius	Right	Thoracic	Convex	Prone	Mechanical stress relaxation time	ms	18,7
S11	Middle trapezius	Right	Thoracic	Convex	Standing	Creep (Deborah number ratio)	dimensionless	1,03
S11	Middle trapezius	Right	Thoracic	Convex	Prone	Creep (Deborah number ratio)	dimensionless	1,13
S11	Lower trapezius	Left	Thoracic	Concave	Standing	Tone (oscillation frequency)	Hz	14,64
S11	Lower trapezius	Left	Thoracic	Concave	Prone	Tone (oscillation frequency)	Hz	16,42
S11	Lower trapezius	Left	Thoracic	Concave	Standing	Dynamic stiffness	N/m	275
S11	Lower trapezius	Left	Thoracic	Concave	Prone	Dynamic stiffness	N/m	373
S11	Lower trapezius	Left	Thoracic	Concave	Standing	Logarithmic decrement (elasticity)	dimensionless	1,13
S11	Lower trapezius	Left	Thoracic	Concave	Prone	Logarithmic decrement (elasticity)	dimensionless	1,14
S11	Lower trapezius	Left	Thoracic	Concave	Standing	Mechanical stress relaxation time	ms	17,5
S11	Lower trapezius	Left	Thoracic	Concave	Prone	Mechanical stress relaxation time	ms	14,6
S11	Lower trapezius	Left	Thoracic	Concave	Standing	Creep (Deborah number ratio)	dimensionless	1,05
S11	Lower trapezius	Left	Thoracic	Concave	Prone	Creep (Deborah number ratio)	dimensionless	0,91
S11	Lower trapezius	Right	Thoracic	Convex	Standing	Tone (oscillation frequency)	Hz	16,46
S11	Lower trapezius	Right	Thoracic	Convex	Prone	Tone (oscillation frequency)	Hz	14,86
S11	Lower trapezius	Right	Thoracic	Convex	Standing	Dynamic stiffness	N/m	356
S11	Lower trapezius	Right	Thoracic	Convex	Prone	Dynamic stiffness	N/m	279
S11	Lower trapezius	Right	Thoracic	Convex	Standing	Logarithmic decrement (elasticity)	dimensionless	1,08
S11	Lower trapezius	Right	Thoracic	Convex	Prone	Logarithmic decrement (elasticity)	dimensionless	1,1
S11	Lower trapezius	Right	Thoracic	Convex	Standing	Mechanical stress relaxation time	ms	14,6
S11	Lower trapezius	Right	Thoracic	Convex	Prone	Mechanical stress relaxation time	ms	17,8
S11	Lower trapezius	Right	Thoracic	Convex	Standing	Creep (Deborah number ratio)	dimensionless	0,91
S11	Lower trapezius	Right	Thoracic	Convex	Prone	Creep (Deborah number ratio)	dimensionless	1,07
S11	Latissimus dorsi	Left	Thoracic/thoracolumbar	Concave	Standing	Tone (oscillation frequency)	Hz	12,22
S11	Latissimus dorsi	Left	Thoracic/thoracolumbar	Concave	Prone	Tone (oscillation frequency)	Hz	13,88
S11	Latissimus dorsi	Left	Thoracic/thoracolumbar	Concave	Standing	Dynamic stiffness	N/m	235
S11	Latissimus dorsi	Left	Thoracic/thoracolumbar	Concave	Prone	Dynamic stiffness	N/m	272
S11	Latissimus dorsi	Left	Thoracic/thoracolumbar	Concave	Standing	Logarithmic decrement (elasticity)	dimensionless	1,16
S11	Latissimus dorsi	Left	Thoracic/thoracolumbar	Concave	Prone	Logarithmic decrement (elasticity)	dimensionless	1,14
S11	Latissimus dorsi	Left	Thoracic/thoracolumbar	Concave	Standing	Mechanical stress relaxation time	ms	19,1
S11	Latissimus dorsi	Left	Thoracic/thoracolumbar	Concave	Prone	Mechanical stress relaxation time	ms	17,4
S11	Latissimus dorsi	Left	Thoracic/thoracolumbar	Concave	Standing	Creep (Deborah number ratio)	dimensionless	1,09
S11	Latissimus dorsi	Left	Thoracic/thoracolumbar	Concave	Prone	Creep (Deborah number ratio)	dimensionless	1,02
S11	Latissimus dorsi	Right	Thoracic/thoracolumbar	Convex	Standing	Tone (oscillation frequency)	Hz	12,52
S11	Latissimus dorsi	Right	Thoracic/thoracolumbar	Convex	Prone	Tone (oscillation frequency)	Hz	13
S11	Latissimus dorsi	Right	Thoracic/thoracolumbar	Convex	Standing	Dynamic stiffness	N/m	222
S11	Latissimus dorsi	Right	Thoracic/thoracolumbar	Convex	Prone	Dynamic stiffness	N/m	221
S11	Latissimus dorsi	Right	Thoracic/thoracolumbar	Convex	Standing	Logarithmic decrement (elasticity)	dimensionless	1,17
S11	Latissimus dorsi	Right	Thoracic/thoracolumbar	Convex	Prone	Logarithmic decrement (elasticity)	dimensionless	0,92
S11	Latissimus dorsi	Right	Thoracic/thoracolumbar	Convex	Standing	Mechanical stress relaxation time	ms	19,6
S11	Latissimus dorsi	Right	Thoracic/thoracolumbar	Convex	Prone	Mechanical stress relaxation time	ms	19,1
S11	Latissimus dorsi	Right	Thoracic/thoracolumbar	Convex	Standing	Creep (Deborah number ratio)	dimensionless	1,11
S11	Latissimus dorsi	Right	Thoracic/thoracolumbar	Convex	Prone	Creep (Deborah number ratio)	dimensionless	1,07
S11	Lumbar erector spinae	Left	Lumbar	Convex	Standing	Tone (oscillation frequency)	Hz	10,92
S11	Lumbar erector spinae	Left	Lumbar	Convex	Prone	Tone (oscillation frequency)	Hz	11,4
S11	Lumbar erector spinae	Left	Lumbar	Convex	Standing	Dynamic stiffness	N/m	140
S11	Lumbar erector spinae	Left	Lumbar	Convex	Prone	Dynamic stiffness	N/m	154
S11	Lumbar erector spinae	Left	Lumbar	Convex	Standing	Logarithmic decrement (elasticity)	dimensionless	0,82
S11	Lumbar erector spinae	Left	Lumbar	Convex	Prone	Logarithmic decrement (elasticity)	dimensionless	0,76
S11	Lumbar erector spinae	Left	Lumbar	Convex	Standing	Mechanical stress relaxation time	ms	24,3
S11	Lumbar erector spinae	Left	Lumbar	Convex	Prone	Mechanical stress relaxation time	ms	22,8
S11	Lumbar erector spinae	Left	Lumbar	Convex	Standing	Creep (Deborah number ratio)	dimensionless	1,27
S11	Lumbar erector spinae	Left	Lumbar	Convex	Prone	Creep (Deborah number ratio)	dimensionless	1,16
S11	Lumbar erector spinae	Right	Lumbar	Concave	Standing	Tone (oscillation frequency)	Hz	11,1
S11	Lumbar erector spinae	Right	Lumbar	Concave	Prone	Tone (oscillation frequency)	Hz	10,5
S11	Lumbar erector spinae	Right	Lumbar	Concave	Standing	Dynamic stiffness	N/m	169
S11	Lumbar erector spinae	Right	Lumbar	Concave	Prone	Dynamic stiffness	N/m	117
S11	Lumbar erector spinae	Right	Lumbar	Concave	Standing	Logarithmic decrement (elasticity)	dimensionless	0,87
S11	Lumbar erector spinae	Right	Lumbar	Concave	Prone	Logarithmic decrement (elasticity)	dimensionless	0,79
S11	Lumbar erector spinae	Right	Lumbar	Concave	Standing	Mechanical stress relaxation time	ms	20,6
S11	Lumbar erector spinae	Right	Lumbar	Concave	Prone	Mechanical stress relaxation time	ms	26,4
S11	Lumbar erector spinae	Right	Lumbar	Concave	Standing	Creep (Deborah number ratio)	dimensionless	1,03
S11	Lumbar erector spinae	Right	Lumbar	Concave	Prone	Creep (Deborah number ratio)	dimensionless	1,3
S12	Middle trapezius	Left	Thoracic	Concave	Standing	Tone (oscillation frequency)	Hz	14,2
S12	Middle trapezius	Left	Thoracic	Concave	Prone	Tone (oscillation frequency)	Hz	13,38
S12	Middle trapezius	Left	Thoracic	Concave	Standing	Dynamic stiffness	N/m	270
S12	Middle trapezius	Left	Thoracic	Concave	Prone	Dynamic stiffness	N/m	223
S12	Middle trapezius	Left	Thoracic	Concave	Standing	Logarithmic decrement (elasticity)	dimensionless	1,11
S12	Middle trapezius	Left	Thoracic	Concave	Prone	Logarithmic decrement (elasticity)	dimensionless	0,94
S12	Middle trapezius	Left	Thoracic	Concave	Standing	Mechanical stress relaxation time	ms	18,3
S12	Middle trapezius	Left	Thoracic	Concave	Prone	Mechanical stress relaxation time	ms	20,2
S12	Middle trapezius	Left	Thoracic	Concave	Standing	Creep (Deborah number ratio)	dimensionless	1,1
S12	Middle trapezius	Left	Thoracic	Concave	Prone	Creep (Deborah number ratio)	dimensionless	1,2
S12	Middle trapezius	Right	Thoracic	Convex	Standing	Tone (oscillation frequency)	Hz	13,1
S12	Middle trapezius	Right	Thoracic	Convex	Prone	Tone (oscillation frequency)	Hz	13,6
S12	Middle trapezius	Right	Thoracic	Convex	Standing	Dynamic stiffness	N/m	209
S12	Middle trapezius	Right	Thoracic	Convex	Prone	Dynamic stiffness	N/m	238
S12	Middle trapezius	Right	Thoracic	Convex	Standing	Logarithmic decrement (elasticity)	dimensionless	0,87
S12	Middle trapezius	Right	Thoracic	Convex	Prone	Logarithmic decrement (elasticity)	dimensionless	1,04
S12	Middle trapezius	Right	Thoracic	Convex	Standing	Mechanical stress relaxation time	ms	21,7
S12	Middle trapezius	Right	Thoracic	Convex	Prone	Mechanical stress relaxation time	ms	19,1
S12	Middle trapezius	Right	Thoracic	Convex	Standing	Creep (Deborah number ratio)	dimensionless	1,29

S12	Middle trapezius	Right	Thoracic	Convex	Prone	Creep (Deborah number ratio)	dimensionless	1,11
S12	Lower trapezius	Left	Thoracic	Concave	Standing	Tone (oscillation frequency)	Hz	14,24
S12	Lower trapezius	Left	Thoracic	Concave	Prone	Tone (oscillation frequency)	Hz	17,72
S12	Lower trapezius	Left	Thoracic	Concave	Standing	Dynamic stiffness	N/m	265
S12	Lower trapezius	Left	Thoracic	Concave	Prone	Dynamic stiffness	N/m	415
S12	Lower trapezius	Left	Thoracic	Concave	Standing	Logarithmic decrement (elasticity)	dimensionless	1,07
S12	Lower trapezius	Left	Thoracic	Concave	Prone	Logarithmic decrement (elasticity)	dimensionless	0,83
S12	Lower trapezius	Left	Thoracic	Concave	Standing	Mechanical stress relaxation time	ms	17,8
S12	Lower trapezius	Left	Thoracic	Concave	Prone	Mechanical stress relaxation time	ms	12,4
S12	Lower trapezius	Left	Thoracic	Concave	Standing	Creep (Deborah number ratio)	dimensionless	1,07
S12	Lower trapezius	Left	Thoracic	Concave	Prone	Creep (Deborah number ratio)	dimensionless	0,77
S12	Lower trapezius	Right	Thoracic	Convex	Standing	Tone (oscillation frequency)	Hz	14,66
S12	Lower trapezius	Right	Thoracic	Convex	Prone	Tone (oscillation frequency)	Hz	16,5
S12	Lower trapezius	Right	Thoracic	Convex	Standing	Dynamic stiffness	N/m	275
S12	Lower trapezius	Right	Thoracic	Convex	Prone	Dynamic stiffness	N/m	359
S12	Lower trapezius	Right	Thoracic	Convex	Standing	Logarithmic decrement (elasticity)	dimensionless	1,04
S12	Lower trapezius	Right	Thoracic	Convex	Prone	Logarithmic decrement (elasticity)	dimensionless	0,93
S12	Lower trapezius	Right	Thoracic	Convex	Standing	Mechanical stress relaxation time	ms	18,5
S12	Lower trapezius	Right	Thoracic	Convex	Prone	Mechanical stress relaxation time	ms	14,2
S12	Lower trapezius	Right	Thoracic	Convex	Standing	Creep (Deborah number ratio)	dimensionless	1,12
S12	Lower trapezius	Right	Thoracic	Convex	Prone	Creep (Deborah number ratio)	dimensionless	0,85
S12	Latissimus dorsi	Left	Thoracic/thoracolumbar	Concave	Standing	Tone (oscillation frequency)	Hz	13,12
S12	Latissimus dorsi	Left	Thoracic/thoracolumbar	Concave	Prone	Tone (oscillation frequency)	Hz	14,66
S12	Latissimus dorsi	Left	Thoracic/thoracolumbar	Concave	Standing	Dynamic stiffness	N/m	246
S12	Latissimus dorsi	Left	Thoracic/thoracolumbar	Concave	Prone	Dynamic stiffness	N/m	282
S12	Latissimus dorsi	Left	Thoracic/thoracolumbar	Concave	Standing	Logarithmic decrement (elasticity)	dimensionless	1,19
S12	Latissimus dorsi	Left	Thoracic/thoracolumbar	Concave	Prone	Logarithmic decrement (elasticity)	dimensionless	1,12
S12	Latissimus dorsi	Left	Thoracic/thoracolumbar	Concave	Standing	Mechanical stress relaxation time	ms	19
S12	Latissimus dorsi	Left	Thoracic/thoracolumbar	Concave	Prone	Mechanical stress relaxation time	ms	17,7
S12	Latissimus dorsi	Left	Thoracic/thoracolumbar	Concave	Standing	Creep (Deborah number ratio)	dimensionless	1,13
S12	Latissimus dorsi	Left	Thoracic/thoracolumbar	Concave	Prone	Creep (Deborah number ratio)	dimensionless	1,07
S12	Latissimus dorsi	Right	Thoracic/thoracolumbar	Convex	Standing	Tone (oscillation frequency)	Hz	16,86
S12	Latissimus dorsi	Right	Thoracic/thoracolumbar	Convex	Prone	Tone (oscillation frequency)	Hz	14,88
S12	Latissimus dorsi	Right	Thoracic/thoracolumbar	Convex	Standing	Dynamic stiffness	N/m	421
S12	Latissimus dorsi	Right	Thoracic/thoracolumbar	Convex	Prone	Dynamic stiffness	N/m	283
S12	Latissimus dorsi	Right	Thoracic/thoracolumbar	Convex	Standing	Logarithmic decrement (elasticity)	dimensionless	0,89
S12	Latissimus dorsi	Right	Thoracic/thoracolumbar	Convex	Prone	Logarithmic decrement (elasticity)	dimensionless	0,91
S12	Latissimus dorsi	Right	Thoracic/thoracolumbar	Convex	Standing	Mechanical stress relaxation time	ms	12,4
S12	Latissimus dorsi	Right	Thoracic/thoracolumbar	Convex	Prone	Mechanical stress relaxation time	ms	16,2
S12	Latissimus dorsi	Right	Thoracic/thoracolumbar	Convex	Standing	Creep (Deborah number ratio)	dimensionless	0,77
S12	Latissimus dorsi	Right	Thoracic/thoracolumbar	Convex	Prone	Creep (Deborah number ratio)	dimensionless	0,93
S12	Lumbar erector spinae	Left	Lumbar	Convex	Standing	Tone (oscillation frequency)	Hz	21
S12	Lumbar erector spinae	Left	Lumbar	Convex	Prone	Tone (oscillation frequency)	Hz	13,52
S12	Lumbar erector spinae	Left	Lumbar	Convex	Standing	Dynamic stiffness	N/m	581
S12	Lumbar erector spinae	Left	Lumbar	Convex	Prone	Dynamic stiffness	N/m	282
S12	Lumbar erector spinae	Left	Lumbar	Convex	Standing	Logarithmic decrement (elasticity)	dimensionless	0,73
S12	Lumbar erector spinae	Left	Lumbar	Convex	Prone	Logarithmic decrement (elasticity)	dimensionless	1,01
S12	Lumbar erector spinae	Left	Lumbar	Convex	Standing	Mechanical stress relaxation time	ms	9,6
S12	Lumbar erector spinae	Left	Lumbar	Convex	Prone	Mechanical stress relaxation time	ms	18,5
S12	Lumbar erector spinae	Left	Lumbar	Convex	Standing	Creep (Deborah number ratio)	dimensionless	0,61
S12	Lumbar erector spinae	Left	Lumbar	Convex	Prone	Creep (Deborah number ratio)	dimensionless	1,12
S12	Lumbar erector spinae	Right	Lumbar	Concave	Standing	Tone (oscillation frequency)	Hz	15,98
S12	Lumbar erector spinae	Right	Lumbar	Concave	Prone	Tone (oscillation frequency)	Hz	12,44
S12	Lumbar erector spinae	Right	Lumbar	Concave	Standing	Dynamic stiffness	N/m	424
S12	Lumbar erector spinae	Right	Lumbar	Concave	Prone	Dynamic stiffness	N/m	237
S12	Lumbar erector spinae	Right	Lumbar	Concave	Standing	Logarithmic decrement (elasticity)	dimensionless	0,96
S12	Lumbar erector spinae	Right	Lumbar	Concave	Prone	Logarithmic decrement (elasticity)	dimensionless	1
S12	Lumbar erector spinae	Right	Lumbar	Concave	Standing	Mechanical stress relaxation time	ms	13
S12	Lumbar erector spinae	Right	Lumbar	Concave	Prone	Mechanical stress relaxation time	ms	21,4
S12	Lumbar erector spinae	Right	Lumbar	Concave	Standing	Creep (Deborah number ratio)	dimensionless	0,82
S12	Lumbar erector spinae	Right	Lumbar	Concave	Prone	Creep (Deborah number ratio)	dimensionless	1,27
S13	Middle trapezius	Left	Thoracic	Concave	Standing	Tone (oscillation frequency)	Hz	13,78
S13	Middle trapezius	Left	Thoracic	Concave	Prone	Tone (oscillation frequency)	Hz	11,2
S13	Middle trapezius	Left	Thoracic	Concave	Standing	Dynamic stiffness	N/m	276
S13	Middle trapezius	Left	Thoracic	Concave	Prone	Dynamic stiffness	N/m	161
S13	Middle trapezius	Left	Thoracic	Concave	Standing	Logarithmic decrement (elasticity)	dimensionless	1,08
S13	Middle trapezius	Left	Thoracic	Concave	Prone	Logarithmic decrement (elasticity)	dimensionless	1,02
S13	Middle trapezius	Left	Thoracic	Concave	Standing	Mechanical stress relaxation time	ms	17,6
S13	Middle trapezius	Left	Thoracic	Concave	Prone	Mechanical stress relaxation time	ms	23,5
S13	Middle trapezius	Left	Thoracic	Concave	Standing	Creep (Deborah number ratio)	dimensionless	1,07
S13	Middle trapezius	Left	Thoracic	Concave	Prone	Creep (Deborah number ratio)	dimensionless	1,32
S13	Middle trapezius	Right	Thoracic	Convex	Standing	Tone (oscillation frequency)	Hz	14,74
S13	Middle trapezius	Right	Thoracic	Convex	Prone	Tone (oscillation frequency)	Hz	11,8
S13	Middle trapezius	Right	Thoracic	Convex	Standing	Dynamic stiffness	N/m	297
S13	Middle trapezius	Right	Thoracic	Convex	Prone	Dynamic stiffness	N/m	176
S13	Middle trapezius	Right	Thoracic	Convex	Standing	Logarithmic decrement (elasticity)	dimensionless	0,99
S13	Middle trapezius	Right	Thoracic	Convex	Prone	Logarithmic decrement (elasticity)	dimensionless	1,26
S13	Middle trapezius	Right	Thoracic	Convex	Standing	Mechanical stress relaxation time	ms	16,7
S13	Middle trapezius	Right	Thoracic	Convex	Prone	Mechanical stress relaxation time	ms	22,9
S13	Middle trapezius	Right	Thoracic	Convex	Standing	Creep (Deborah number ratio)	dimensionless	1,02
S13	Middle trapezius	Right	Thoracic	Convex	Prone	Creep (Deborah number ratio)	dimensionless	1,34
S13	Lower trapezius	Left	Thoracic	Concave	Standing	Tone (oscillation frequency)	Hz	14,92
S13	Lower trapezius	Left	Thoracic	Concave	Prone	Tone (oscillation frequency)	Hz	14,52
S13	Lower trapezius	Left	Thoracic	Concave	Standing	Dynamic stiffness	N/m	312
S13	Lower trapezius	Left	Thoracic	Concave	Prone	Dynamic stiffness	N/m	333
S13	Lower trapezius	Left	Thoracic	Concave	Standing	Logarithmic decrement (elasticity)	dimensionless	1,27
S13	Lower trapezius	Left	Thoracic	Concave	Prone	Logarithmic decrement (elasticity)	dimensionless	1,39
S13	Lower trapezius	Left	Thoracic	Concave	Standing	Mechanical stress relaxation time	ms	16,4
S13	Lower trapezius	Left	Thoracic	Concave	Prone	Mechanical stress relaxation time	ms	15,4
S13	Lower trapezius	Left	Thoracic	Concave	Standing	Creep (Deborah number ratio)	dimensionless	0,99

S13	Lower trapezius	Left	Thoracic	Concave	Prone	Creep (Deborah number ratio)	dimensionless	0,93
S13	Lower trapezius	Right	Thoracic	Convex	Standing	Tone (oscillation frequency)	Hz	16,68
S13	Lower trapezius	Right	Thoracic	Convex	Prone	Tone (oscillation frequency)	Hz	13,38
S13	Lower trapezius	Right	Thoracic	Convex	Standing	Dynamic stiffness	N/m	432
S13	Lower trapezius	Right	Thoracic	Convex	Prone	Dynamic stiffness	N/m	227
S13	Lower trapezius	Right	Thoracic	Convex	Standing	Logarithmic decrement (elasticity)	dimensionless	1,13
S13	Lower trapezius	Right	Thoracic	Convex	Prone	Logarithmic decrement (elasticity)	dimensionless	1,22
S13	Lower trapezius	Right	Thoracic	Convex	Standing	Mechanical stress relaxation time	ms	11,9
S13	Lower trapezius	Right	Thoracic	Convex	Prone	Mechanical stress relaxation time	ms	19,4
S13	Lower trapezius	Right	Thoracic	Convex	Standing	Creep (Deborah number ratio)	dimensionless	0,74
S13	Lower trapezius	Right	Thoracic	Convex	Prone	Creep (Deborah number ratio)	dimensionless	1,11
S13	Latissimus dorsi	Left	Thoracic/thoracolumbar	Concave	Standing	Tone (oscillation frequency)	Hz	11,7
S13	Latissimus dorsi	Left	Thoracic/thoracolumbar	Concave	Prone	Tone (oscillation frequency)	Hz	15,44
S13	Latissimus dorsi	Left	Thoracic/thoracolumbar	Concave	Standing	Dynamic stiffness	N/m	207
S13	Latissimus dorsi	Left	Thoracic/thoracolumbar	Concave	Prone	Dynamic stiffness	N/m	336
S13	Latissimus dorsi	Left	Thoracic/thoracolumbar	Concave	Standing	Logarithmic decrement (elasticity)	dimensionless	1,28
S13	Latissimus dorsi	Left	Thoracic/thoracolumbar	Concave	Prone	Logarithmic decrement (elasticity)	dimensionless	1,21
S13	Latissimus dorsi	Left	Thoracic/thoracolumbar	Concave	Standing	Mechanical stress relaxation time	ms	22,3
S13	Latissimus dorsi	Left	Thoracic/thoracolumbar	Concave	Prone	Mechanical stress relaxation time	ms	16,4
S13	Latissimus dorsi	Left	Thoracic/thoracolumbar	Concave	Standing	Creep (Deborah number ratio)	dimensionless	1,35
S13	Latissimus dorsi	Left	Thoracic/thoracolumbar	Concave	Prone	Creep (Deborah number ratio)	dimensionless	1,03
S13	Latissimus dorsi	Right	Thoracic/thoracolumbar	Convex	Standing	Tone (oscillation frequency)	Hz	12,18
S13	Latissimus dorsi	Right	Thoracic/thoracolumbar	Convex	Prone	Tone (oscillation frequency)	Hz	14,88
S13	Latissimus dorsi	Right	Thoracic/thoracolumbar	Convex	Standing	Dynamic stiffness	N/m	247
S13	Latissimus dorsi	Right	Thoracic/thoracolumbar	Convex	Prone	Dynamic stiffness	N/m	311
S13	Latissimus dorsi	Right	Thoracic/thoracolumbar	Convex	Standing	Logarithmic decrement (elasticity)	dimensionless	1,32
S13	Latissimus dorsi	Right	Thoracic/thoracolumbar	Convex	Prone	Logarithmic decrement (elasticity)	dimensionless	1,14
S13	Latissimus dorsi	Right	Thoracic/thoracolumbar	Convex	Standing	Mechanical stress relaxation time	ms	18,7
S13	Latissimus dorsi	Right	Thoracic/thoracolumbar	Convex	Prone	Mechanical stress relaxation time	ms	16,3
S13	Latissimus dorsi	Right	Thoracic/thoracolumbar	Convex	Standing	Creep (Deborah number ratio)	dimensionless	1,09
S13	Latissimus dorsi	Right	Thoracic/thoracolumbar	Convex	Prone	Creep (Deborah number ratio)	dimensionless	0,99
S13	Lumbar erector spinae	Left	Lumbar	Convex	Standing	Tone (oscillation frequency)	Hz	10,62
S13	Lumbar erector spinae	Left	Lumbar	Convex	Prone	Tone (oscillation frequency)	Hz	10,5
S13	Lumbar erector spinae	Left	Lumbar	Convex	Standing	Dynamic stiffness	N/m	148
S13	Lumbar erector spinae	Left	Lumbar	Convex	Prone	Dynamic stiffness	N/m	147
S13	Lumbar erector spinae	Left	Lumbar	Convex	Standing	Logarithmic decrement (elasticity)	dimensionless	0,81
S13	Lumbar erector spinae	Left	Lumbar	Convex	Prone	Logarithmic decrement (elasticity)	dimensionless	0,92
S13	Lumbar erector spinae	Left	Lumbar	Convex	Standing	Mechanical stress relaxation time	ms	21,6
S13	Lumbar erector spinae	Left	Lumbar	Convex	Prone	Mechanical stress relaxation time	ms	23,4
S13	Lumbar erector spinae	Left	Lumbar	Convex	Standing	Creep (Deborah number ratio)	dimensionless	1,05
S13	Lumbar erector spinae	Left	Lumbar	Convex	Prone	Creep (Deborah number ratio)	dimensionless	1,22
S13	Lumbar erector spinae	Right	Lumbar	Concave	Standing	Tone (oscillation frequency)	Hz	10,68
S13	Lumbar erector spinae	Right	Lumbar	Concave	Prone	Tone (oscillation frequency)	Hz	9,9
S13	Lumbar erector spinae	Right	Lumbar	Concave	Standing	Dynamic stiffness	N/m	136
S13	Lumbar erector spinae	Right	Lumbar	Concave	Prone	Dynamic stiffness	N/m	103
S13	Lumbar erector spinae	Right	Lumbar	Concave	Standing	Logarithmic decrement (elasticity)	dimensionless	0,77
S13	Lumbar erector spinae	Right	Lumbar	Concave	Prone	Logarithmic decrement (elasticity)	dimensionless	0,74
S13	Lumbar erector spinae	Right	Lumbar	Concave	Standing	Mechanical stress relaxation time	ms	22,2
S13	Lumbar erector spinae	Right	Lumbar	Concave	Prone	Mechanical stress relaxation time	ms	29
S13	Lumbar erector spinae	Right	Lumbar	Concave	Standing	Creep (Deborah number ratio)	dimensionless	1,04
S13	Lumbar erector spinae	Right	Lumbar	Concave	Prone	Creep (Deborah number ratio)	dimensionless	1,49
S14	Middle trapezius	Left	Thoracic	Concave	Standing	Tone (oscillation frequency)	Hz	13,8
S14	Middle trapezius	Left	Thoracic	Concave	Prone	Tone (oscillation frequency)	Hz	13,32
S14	Middle trapezius	Left	Thoracic	Concave	Standing	Dynamic stiffness	N/m	217
S14	Middle trapezius	Left	Thoracic	Concave	Prone	Dynamic stiffness	N/m	210
S14	Middle trapezius	Left	Thoracic	Concave	Standing	Logarithmic decrement (elasticity)	dimensionless	0,87
S14	Middle trapezius	Left	Thoracic	Concave	Prone	Logarithmic decrement (elasticity)	dimensionless	0,95
S14	Middle trapezius	Left	Thoracic	Concave	Standing	Mechanical stress relaxation time	ms	20,8
S14	Middle trapezius	Left	Thoracic	Concave	Prone	Mechanical stress relaxation time	ms	19,9
S14	Middle trapezius	Left	Thoracic	Concave	Standing	Creep (Deborah number ratio)	dimensionless	1,22
S14	Middle trapezius	Left	Thoracic	Concave	Prone	Creep (Deborah number ratio)	dimensionless	1,13
S14	Middle trapezius	Right	Thoracic	Convex	Standing	Tone (oscillation frequency)	Hz	14,74
S14	Middle trapezius	Right	Thoracic	Convex	Prone	Tone (oscillation frequency)	Hz	12,86
S14	Middle trapezius	Right	Thoracic	Convex	Standing	Dynamic stiffness	N/m	245
S14	Middle trapezius	Right	Thoracic	Convex	Prone	Dynamic stiffness	N/m	180
S14	Middle trapezius	Right	Thoracic	Convex	Standing	Logarithmic decrement (elasticity)	dimensionless	0,85
S14	Middle trapezius	Right	Thoracic	Convex	Prone	Logarithmic decrement (elasticity)	dimensionless	0,87
S14	Middle trapezius	Right	Thoracic	Convex	Standing	Mechanical stress relaxation time	ms	19,4
S14	Middle trapezius	Right	Thoracic	Convex	Prone	Mechanical stress relaxation time	ms	22,3
S14	Middle trapezius	Right	Thoracic	Convex	Standing	Creep (Deborah number ratio)	dimensionless	1,15
S14	Middle trapezius	Right	Thoracic	Convex	Prone	Creep (Deborah number ratio)	dimensionless	1,28
S14	Lower trapezius	Left	Thoracic	Concave	Standing	Tone (oscillation frequency)	Hz	14,34
S14	Lower trapezius	Left	Thoracic	Concave	Prone	Tone (oscillation frequency)	Hz	15,28
S14	Lower trapezius	Left	Thoracic	Concave	Standing	Dynamic stiffness	N/m	257
S14	Lower trapezius	Left	Thoracic	Concave	Prone	Dynamic stiffness	N/m	290
S14	Lower trapezius	Left	Thoracic	Concave	Standing	Logarithmic decrement (elasticity)	dimensionless	1,05
S14	Lower trapezius	Left	Thoracic	Concave	Prone	Logarithmic decrement (elasticity)	dimensionless	1,02
S14	Lower trapezius	Left	Thoracic	Concave	Standing	Mechanical stress relaxation time	ms	18,7
S14	Lower trapezius	Left	Thoracic	Concave	Prone	Mechanical stress relaxation time	ms	16,2
S14	Lower trapezius	Left	Thoracic	Concave	Standing	Creep (Deborah number ratio)	dimensionless	1,11
S14	Lower trapezius	Left	Thoracic	Concave	Prone	Creep (Deborah number ratio)	dimensionless	0,93
S14	Lower trapezius	Right	Thoracic	Convex	Standing	Tone (oscillation frequency)	Hz	15,68
S14	Lower trapezius	Right	Thoracic	Convex	Prone	Tone (oscillation frequency)	Hz	14,68
S14	Lower trapezius	Right	Thoracic	Convex	Standing	Dynamic stiffness	N/m	296
S14	Lower trapezius	Right	Thoracic	Convex	Prone	Dynamic stiffness	N/m	269
S14	Lower trapezius	Right	Thoracic	Convex	Standing	Logarithmic decrement (elasticity)	dimensionless	0,9
S14	Lower trapezius	Right	Thoracic	Convex	Prone	Logarithmic decrement (elasticity)	dimensionless	1,05
S14	Lower trapezius	Right	Thoracic	Convex	Standing	Mechanical stress relaxation time	ms	17,1
S14	Lower trapezius	Right	Thoracic	Convex	Prone	Mechanical stress relaxation time	ms	17,6
S14	Lower trapezius	Right	Thoracic	Convex	Standing	Creep (Deborah number ratio)	dimensionless	1,02

S14	Lower trapezius	Right	Thoracic	Convex	Prone	Creep (Deborah number ratio)	dimensionless	1,02
S14	Latissimus dorsi	Left	Thoracic/thoracolumbar	Concave	Standing	Tone (oscillation frequency)	Hz	11,74
S14	Latissimus dorsi	Left	Thoracic/thoracolumbar	Concave	Prone	Tone (oscillation frequency)	Hz	12,8
S14	Latissimus dorsi	Left	Thoracic/thoracolumbar	Concave	Standing	Dynamic stiffness	N/m	194
S14	Latissimus dorsi	Left	Thoracic/thoracolumbar	Concave	Prone	Dynamic stiffness	N/m	224
S14	Latissimus dorsi	Left	Thoracic/thoracolumbar	Concave	Standing	Logarithmic decrement (elasticity)	dimensionless	1,14
S14	Latissimus dorsi	Left	Thoracic/thoracolumbar	Concave	Prone	Logarithmic decrement (elasticity)	dimensionless	1,07
S14	Latissimus dorsi	Left	Thoracic/thoracolumbar	Concave	Standing	Mechanical stress relaxation time	ms	21,5
S14	Latissimus dorsi	Left	Thoracic/thoracolumbar	Concave	Prone	Mechanical stress relaxation time	ms	19,1
S14	Latissimus dorsi	Left	Thoracic/thoracolumbar	Concave	Standing	Creep (Deborah number ratio)	dimensionless	1,23
S14	Latissimus dorsi	Left	Thoracic/thoracolumbar	Concave	Prone	Creep (Deborah number ratio)	dimensionless	1,06
S14	Latissimus dorsi	Right	Thoracic/thoracolumbar	Convex	Standing	Tone (oscillation frequency)	Hz	13,22
S14	Latissimus dorsi	Right	Thoracic/thoracolumbar	Convex	Prone	Tone (oscillation frequency)	Hz	12,82
S14	Latissimus dorsi	Right	Thoracic/thoracolumbar	Convex	Standing	Dynamic stiffness	N/m	251
S14	Latissimus dorsi	Right	Thoracic/thoracolumbar	Convex	Prone	Dynamic stiffness	N/m	198
S14	Latissimus dorsi	Right	Thoracic/thoracolumbar	Convex	Standing	Logarithmic decrement (elasticity)	dimensionless	1,05
S14	Latissimus dorsi	Right	Thoracic/thoracolumbar	Convex	Prone	Logarithmic decrement (elasticity)	dimensionless	0,95
S14	Latissimus dorsi	Right	Thoracic/thoracolumbar	Convex	Standing	Mechanical stress relaxation time	ms	18,1
S14	Latissimus dorsi	Right	Thoracic/thoracolumbar	Convex	Prone	Mechanical stress relaxation time	ms	20,8
S14	Latissimus dorsi	Right	Thoracic/thoracolumbar	Convex	Standing	Creep (Deborah number ratio)	dimensionless	1,04
S14	Latissimus dorsi	Right	Thoracic/thoracolumbar	Convex	Prone	Creep (Deborah number ratio)	dimensionless	1,17
S14	Lumbar erector spinae	Left	Lumbar	Convex	Standing	Tone (oscillation frequency)	Hz	11,68
S14	Lumbar erector spinae	Left	Lumbar	Convex	Prone	Tone (oscillation frequency)	Hz	13,08
S14	Lumbar erector spinae	Left	Lumbar	Convex	Standing	Dynamic stiffness	N/m	187
S14	Lumbar erector spinae	Left	Lumbar	Convex	Prone	Dynamic stiffness	N/m	256
S14	Lumbar erector spinae	Left	Lumbar	Convex	Standing	Logarithmic decrement (elasticity)	dimensionless	0,91
S14	Lumbar erector spinae	Left	Lumbar	Convex	Prone	Logarithmic decrement (elasticity)	dimensionless	0,97
S14	Lumbar erector spinae	Left	Lumbar	Convex	Standing	Mechanical stress relaxation time	ms	21,2
S14	Lumbar erector spinae	Left	Lumbar	Convex	Prone	Mechanical stress relaxation time	ms	18
S14	Lumbar erector spinae	Left	Lumbar	Convex	Standing	Creep (Deborah number ratio)	dimensionless	1,18
S14	Lumbar erector spinae	Left	Lumbar	Convex	Prone	Creep (Deborah number ratio)	dimensionless	1,04
S14	Lumbar erector spinae	Right	Lumbar	Concave	Standing	Tone (oscillation frequency)	Hz	10,54
S14	Lumbar erector spinae	Right	Lumbar	Concave	Prone	Tone (oscillation frequency)	Hz	11,94
S14	Lumbar erector spinae	Right	Lumbar	Concave	Standing	Dynamic stiffness	N/m	131
S14	Lumbar erector spinae	Right	Lumbar	Concave	Prone	Dynamic stiffness	N/m	188
S14	Lumbar erector spinae	Right	Lumbar	Concave	Standing	Logarithmic decrement (elasticity)	dimensionless	0,76
S14	Lumbar erector spinae	Right	Lumbar	Concave	Prone	Logarithmic decrement (elasticity)	dimensionless	0,96
S14	Lumbar erector spinae	Right	Lumbar	Concave	Standing	Mechanical stress relaxation time	ms	25,2
S14	Lumbar erector spinae	Right	Lumbar	Concave	Prone	Mechanical stress relaxation time	ms	22,9
S14	Lumbar erector spinae	Right	Lumbar	Concave	Standing	Creep (Deborah number ratio)	dimensionless	1,33
S14	Lumbar erector spinae	Right	Lumbar	Concave	Prone	Creep (Deborah number ratio)	dimensionless	1,3
S15	Middle trapezius	Left	Thoracic	Concave	Standing	Tone (oscillation frequency)	Hz	16,44
S15	Middle trapezius	Left	Thoracic	Concave	Prone	Tone (oscillation frequency)	Hz	14,94
S15	Middle trapezius	Left	Thoracic	Concave	Standing	Dynamic stiffness	N/m	344
S15	Middle trapezius	Left	Thoracic	Concave	Prone	Dynamic stiffness	N/m	281
S15	Middle trapezius	Left	Thoracic	Concave	Standing	Logarithmic decrement (elasticity)	dimensionless	0,7
S15	Middle trapezius	Left	Thoracic	Concave	Prone	Logarithmic decrement (elasticity)	dimensionless	0,97
S15	Middle trapezius	Left	Thoracic	Concave	Standing	Mechanical stress relaxation time	ms	13,8
S15	Middle trapezius	Left	Thoracic	Concave	Prone	Mechanical stress relaxation time	ms	16,8
S15	Middle trapezius	Left	Thoracic	Concave	Standing	Creep (Deborah number ratio)	dimensionless	0,82
S15	Middle trapezius	Left	Thoracic	Concave	Prone	Creep (Deborah number ratio)	dimensionless	0,99
S15	Middle trapezius	Right	Thoracic	Convex	Standing	Tone (oscillation frequency)	Hz	17,28
S15	Middle trapezius	Right	Thoracic	Convex	Prone	Tone (oscillation frequency)	Hz	14,68
S15	Middle trapezius	Right	Thoracic	Convex	Standing	Dynamic stiffness	N/m	383
S15	Middle trapezius	Right	Thoracic	Convex	Prone	Dynamic stiffness	N/m	270
S15	Middle trapezius	Right	Thoracic	Convex	Standing	Logarithmic decrement (elasticity)	dimensionless	0,76
S15	Middle trapezius	Right	Thoracic	Convex	Prone	Logarithmic decrement (elasticity)	dimensionless	0,94
S15	Middle trapezius	Right	Thoracic	Convex	Standing	Mechanical stress relaxation time	ms	13
S15	Middle trapezius	Right	Thoracic	Convex	Prone	Mechanical stress relaxation time	ms	18,1
S15	Middle trapezius	Right	Thoracic	Convex	Standing	Creep (Deborah number ratio)	dimensionless	0,8
S15	Middle trapezius	Right	Thoracic	Convex	Prone	Creep (Deborah number ratio)	dimensionless	1,06
S15	Lower trapezius	Left	Thoracic	Concave	Standing	Tone (oscillation frequency)	Hz	14,88
S15	Lower trapezius	Left	Thoracic	Concave	Prone	Tone (oscillation frequency)	Hz	18,32
S15	Lower trapezius	Left	Thoracic	Concave	Standing	Dynamic stiffness	N/m	260
S15	Lower trapezius	Left	Thoracic	Concave	Prone	Dynamic stiffness	N/m	431
S15	Lower trapezius	Left	Thoracic	Concave	Standing	Logarithmic decrement (elasticity)	dimensionless	0,85
S15	Lower trapezius	Left	Thoracic	Concave	Prone	Logarithmic decrement (elasticity)	dimensionless	1,06
S15	Lower trapezius	Left	Thoracic	Concave	Standing	Mechanical stress relaxation time	ms	17,5
S15	Lower trapezius	Left	Thoracic	Concave	Prone	Mechanical stress relaxation time	ms	12,6
S15	Lower trapezius	Left	Thoracic	Concave	Standing	Creep (Deborah number ratio)	dimensionless	1,01
S15	Lower trapezius	Left	Thoracic	Concave	Prone	Creep (Deborah number ratio)	dimensionless	0,78
S15	Lower trapezius	Right	Thoracic	Convex	Standing	Tone (oscillation frequency)	Hz	16,04
S15	Lower trapezius	Right	Thoracic	Convex	Prone	Tone (oscillation frequency)	Hz	16,8
S15	Lower trapezius	Right	Thoracic	Convex	Standing	Dynamic stiffness	N/m	318
S15	Lower trapezius	Right	Thoracic	Convex	Prone	Dynamic stiffness	N/m	348
S15	Lower trapezius	Right	Thoracic	Convex	Standing	Logarithmic decrement (elasticity)	dimensionless	1,06
S15	Lower trapezius	Right	Thoracic	Convex	Prone	Logarithmic decrement (elasticity)	dimensionless	1,06
S15	Lower trapezius	Right	Thoracic	Convex	Standing	Mechanical stress relaxation time	ms	15,5
S15	Lower trapezius	Right	Thoracic	Convex	Prone	Mechanical stress relaxation time	ms	14,5
S15	Lower trapezius	Right	Thoracic	Convex	Standing	Creep (Deborah number ratio)	dimensionless	0,95
S15	Lower trapezius	Right	Thoracic	Convex	Prone	Creep (Deborah number ratio)	dimensionless	0,89
S15	Latissimus dorsi	Left	Thoracic/thoracolumbar	Concave	Standing	Tone (oscillation frequency)	Hz	12,14
S15	Latissimus dorsi	Left	Thoracic/thoracolumbar	Concave	Prone	Tone (oscillation frequency)	Hz	18,28
S15	Latissimus dorsi	Left	Thoracic/thoracolumbar	Concave	Standing	Dynamic stiffness	N/m	201
S15	Latissimus dorsi	Left	Thoracic/thoracolumbar	Concave	Prone	Dynamic stiffness	N/m	481
S15	Latissimus dorsi	Left	Thoracic/thoracolumbar	Concave	Standing	Logarithmic decrement (elasticity)	dimensionless	0,97
S15	Latissimus dorsi	Left	Thoracic/thoracolumbar	Concave	Prone	Logarithmic decrement (elasticity)	dimensionless	0,93
S15	Latissimus dorsi	Left	Thoracic/thoracolumbar	Concave	Standing	Mechanical stress relaxation time	ms	20,2
S15	Latissimus dorsi	Left	Thoracic/thoracolumbar	Concave	Prone	Mechanical stress relaxation time	ms	11,1
S15	Latissimus dorsi	Left	Thoracic/thoracolumbar	Concave	Standing	Creep (Deborah number ratio)	dimensionless	1,16

S15	Latissimus dorsi	Left	Thoracic/thoracolumbar	Concave	Prone	Creep (Deborah number ratio)	dimensionless	0,69
S15	Latissimus dorsi	Right	Thoracic/thoracolumbar	Convex	Standing	Tone (oscillation frequency)	Hz	11,44
S15	Latissimus dorsi	Right	Thoracic/thoracolumbar	Convex	Prone	Tone (oscillation frequency)	Hz	15,72
S15	Latissimus dorsi	Right	Thoracic/thoracolumbar	Convex	Standing	Dynamic stiffness	N/m	173
S15	Latissimus dorsi	Right	Thoracic/thoracolumbar	Convex	Prone	Dynamic stiffness	N/m	299
S15	Latissimus dorsi	Right	Thoracic/thoracolumbar	Convex	Standing	Logarithmic decrement (elasticity)	dimensionless	1,11
S15	Latissimus dorsi	Right	Thoracic/thoracolumbar	Convex	Prone	Logarithmic decrement (elasticity)	dimensionless	0,83
S15	Latissimus dorsi	Right	Thoracic/thoracolumbar	Convex	Standing	Mechanical stress relaxation time	ms	22,2
S15	Latissimus dorsi	Right	Thoracic/thoracolumbar	Convex	Prone	Mechanical stress relaxation time	ms	14,9
S15	Latissimus dorsi	Right	Thoracic/thoracolumbar	Convex	Standing	Creep (Deborah number ratio)	dimensionless	1,22
S15	Latissimus dorsi	Right	Thoracic/thoracolumbar	Convex	Prone	Creep (Deborah number ratio)	dimensionless	0,84
S15	Lumbar erector spinae	Left	Lumbar	Convex	Standing	Tone (oscillation frequency)	Hz	11,98
S15	Lumbar erector spinae	Left	Lumbar	Convex	Prone	Tone (oscillation frequency)	Hz	13,22
S15	Lumbar erector spinae	Left	Lumbar	Convex	Standing	Dynamic stiffness	N/m	153
S15	Lumbar erector spinae	Left	Lumbar	Convex	Prone	Dynamic stiffness	N/m	197
S15	Lumbar erector spinae	Left	Lumbar	Convex	Standing	Logarithmic decrement (elasticity)	dimensionless	0,7
S15	Lumbar erector spinae	Left	Lumbar	Convex	Prone	Logarithmic decrement (elasticity)	dimensionless	0,63
S15	Lumbar erector spinae	Left	Lumbar	Convex	Standing	Mechanical stress relaxation time	ms	19,8
S15	Lumbar erector spinae	Left	Lumbar	Convex	Prone	Mechanical stress relaxation time	ms	17,9
S15	Lumbar erector spinae	Left	Lumbar	Convex	Standing	Creep (Deborah number ratio)	dimensionless	0,95
S15	Lumbar erector spinae	Left	Lumbar	Convex	Prone	Creep (Deborah number ratio)	dimensionless	0,86
S15	Lumbar erector spinae	Right	Lumbar	Concave	Standing	Tone (oscillation frequency)	Hz	12,4
S15	Lumbar erector spinae	Right	Lumbar	Concave	Prone	Tone (oscillation frequency)	Hz	12,26
S15	Lumbar erector spinae	Right	Lumbar	Concave	Standing	Dynamic stiffness	N/m	176
S15	Lumbar erector spinae	Right	Lumbar	Concave	Prone	Dynamic stiffness	N/m	167
S15	Lumbar erector spinae	Right	Lumbar	Concave	Standing	Logarithmic decrement (elasticity)	dimensionless	0,7
S15	Lumbar erector spinae	Right	Lumbar	Concave	Prone	Logarithmic decrement (elasticity)	dimensionless	0,59
S15	Lumbar erector spinae	Right	Lumbar	Concave	Standing	Mechanical stress relaxation time	ms	18,4
S15	Lumbar erector spinae	Right	Lumbar	Concave	Prone	Mechanical stress relaxation time	ms	21
S15	Lumbar erector spinae	Right	Lumbar	Concave	Standing	Creep (Deborah number ratio)	dimensionless	0,89
S15	Lumbar erector spinae	Right	Lumbar	Concave	Prone	Creep (Deborah number ratio)	dimensionless	1,06
S16	Middle trapezius	Left	Thoracic	Concave	Standing	Tone (oscillation frequency)	Hz	17,26
S16	Middle trapezius	Left	Thoracic	Concave	Prone	Tone (oscillation frequency)	Hz	15,74
S16	Middle trapezius	Left	Thoracic	Concave	Standing	Dynamic stiffness	N/m	415
S16	Middle trapezius	Left	Thoracic	Concave	Prone	Dynamic stiffness	N/m	327
S16	Middle trapezius	Left	Thoracic	Concave	Standing	Logarithmic decrement (elasticity)	dimensionless	1,21
S16	Middle trapezius	Left	Thoracic	Concave	Prone	Logarithmic decrement (elasticity)	dimensionless	1,15
S16	Middle trapezius	Left	Thoracic	Concave	Standing	Mechanical stress relaxation time	ms	13,1
S16	Middle trapezius	Left	Thoracic	Concave	Prone	Mechanical stress relaxation time	ms	15,2
S16	Middle trapezius	Left	Thoracic	Concave	Standing	Creep (Deborah number ratio)	dimensionless	0,82
S16	Middle trapezius	Left	Thoracic	Concave	Prone	Creep (Deborah number ratio)	dimensionless	0,92
S16	Middle trapezius	Right	Thoracic	Convex	Standing	Tone (oscillation frequency)	Hz	15,98
S16	Middle trapezius	Right	Thoracic	Convex	Prone	Tone (oscillation frequency)	Hz	15,32
S16	Middle trapezius	Right	Thoracic	Convex	Standing	Dynamic stiffness	N/m	303
S16	Middle trapezius	Right	Thoracic	Convex	Prone	Dynamic stiffness	N/m	338
S16	Middle trapezius	Right	Thoracic	Convex	Standing	Logarithmic decrement (elasticity)	dimensionless	0,9
S16	Middle trapezius	Right	Thoracic	Convex	Prone	Logarithmic decrement (elasticity)	dimensionless	1,31
S16	Middle trapezius	Right	Thoracic	Convex	Standing	Mechanical stress relaxation time	ms	16,4
S16	Middle trapezius	Right	Thoracic	Convex	Prone	Mechanical stress relaxation time	ms	15,1
S16	Middle trapezius	Right	Thoracic	Convex	Standing	Creep (Deborah number ratio)	dimensionless	1
S16	Middle trapezius	Right	Thoracic	Convex	Prone	Creep (Deborah number ratio)	dimensionless	0,92
S16	Lower trapezius	Left	Thoracic	Concave	Standing	Tone (oscillation frequency)	Hz	16,06
S16	Lower trapezius	Left	Thoracic	Concave	Prone	Tone (oscillation frequency)	Hz	15,62
S16	Lower trapezius	Left	Thoracic	Concave	Standing	Dynamic stiffness	N/m	356
S16	Lower trapezius	Left	Thoracic	Concave	Prone	Dynamic stiffness	N/m	345
S16	Lower trapezius	Left	Thoracic	Concave	Standing	Logarithmic decrement (elasticity)	dimensionless	1,3
S16	Lower trapezius	Left	Thoracic	Concave	Prone	Logarithmic decrement (elasticity)	dimensionless	1,21
S16	Lower trapezius	Left	Thoracic	Concave	Standing	Mechanical stress relaxation time	ms	14,8
S16	Lower trapezius	Left	Thoracic	Concave	Prone	Mechanical stress relaxation time	ms	14,8
S16	Lower trapezius	Left	Thoracic	Concave	Standing	Creep (Deborah number ratio)	dimensionless	0,92
S16	Lower trapezius	Left	Thoracic	Concave	Prone	Creep (Deborah number ratio)	dimensionless	0,91
S16	Lower trapezius	Right	Thoracic	Convex	Standing	Tone (oscillation frequency)	Hz	15,3
S16	Lower trapezius	Right	Thoracic	Convex	Prone	Tone (oscillation frequency)	Hz	14,58
S16	Lower trapezius	Right	Thoracic	Convex	Standing	Dynamic stiffness	N/m	299
S16	Lower trapezius	Right	Thoracic	Convex	Prone	Dynamic stiffness	N/m	244
S16	Lower trapezius	Right	Thoracic	Convex	Standing	Logarithmic decrement (elasticity)	dimensionless	1,04
S16	Lower trapezius	Right	Thoracic	Convex	Prone	Logarithmic decrement (elasticity)	dimensionless	0,94
S16	Lower trapezius	Right	Thoracic	Convex	Standing	Mechanical stress relaxation time	ms	16,6
S16	Lower trapezius	Right	Thoracic	Convex	Prone	Mechanical stress relaxation time	ms	18,4
S16	Lower trapezius	Right	Thoracic	Convex	Standing	Creep (Deborah number ratio)	dimensionless	1
S16	Lower trapezius	Right	Thoracic	Convex	Prone	Creep (Deborah number ratio)	dimensionless	1,06
S16	Latissimus dorsi	Left	Thoracic/thoracolumbar	Concave	Standing	Tone (oscillation frequency)	Hz	14,16
S16	Latissimus dorsi	Left	Thoracic/thoracolumbar	Concave	Prone	Tone (oscillation frequency)	Hz	16,62
S16	Latissimus dorsi	Left	Thoracic/thoracolumbar	Concave	Standing	Dynamic stiffness	N/m	298
S16	Latissimus dorsi	Left	Thoracic/thoracolumbar	Concave	Prone	Dynamic stiffness	N/m	405
S16	Latissimus dorsi	Left	Thoracic/thoracolumbar	Concave	Standing	Logarithmic decrement (elasticity)	dimensionless	1,23
S16	Latissimus dorsi	Left	Thoracic/thoracolumbar	Concave	Prone	Logarithmic decrement (elasticity)	dimensionless	1,1
S16	Latissimus dorsi	Left	Thoracic/thoracolumbar	Concave	Standing	Mechanical stress relaxation time	ms	16,7
S16	Latissimus dorsi	Left	Thoracic/thoracolumbar	Concave	Prone	Mechanical stress relaxation time	ms	13,4
S16	Latissimus dorsi	Left	Thoracic/thoracolumbar	Concave	Standing	Creep (Deborah number ratio)	dimensionless	1,01
S16	Latissimus dorsi	Left	Thoracic/thoracolumbar	Concave	Prone	Creep (Deborah number ratio)	dimensionless	0,84
S16	Latissimus dorsi	Right	Thoracic/thoracolumbar	Convex	Standing	Tone (oscillation frequency)	Hz	11,84
S16	Latissimus dorsi	Right	Thoracic/thoracolumbar	Convex	Prone	Tone (oscillation frequency)	Hz	13,84
S16	Latissimus dorsi	Right	Thoracic/thoracolumbar	Convex	Standing	Dynamic stiffness	N/m	200
S16	Latissimus dorsi	Right	Thoracic/thoracolumbar	Convex	Prone	Dynamic stiffness	N/m	243
S16	Latissimus dorsi	Right	Thoracic/thoracolumbar	Convex	Standing	Logarithmic decrement (elasticity)	dimensionless	0,93
S16	Latissimus dorsi	Right	Thoracic/thoracolumbar	Convex	Prone	Logarithmic decrement (elasticity)	dimensionless	0,99
S16	Latissimus dorsi	Right	Thoracic/thoracolumbar	Convex	Standing	Mechanical stress relaxation time	ms	19,2
S16	Latissimus dorsi	Right	Thoracic/thoracolumbar	Convex	Prone	Mechanical stress relaxation time	ms	18,4
S16	Latissimus dorsi	Right	Thoracic/thoracolumbar	Convex	Standing	Creep (Deborah number ratio)	dimensionless	1,04

S16	Latissimus dorsi	Right	Thoracic/thoracolumbar	Convex	Prone	Creep (Deborah number ratio)	dimensionless	1,05
S16	Lumbar erector spinae	Left	Lumbar	Convex	Standing	Tone (oscillation frequency)	Hz	10,64
S16	Lumbar erector spinae	Left	Lumbar	Convex	Prone	Tone (oscillation frequency)	Hz	14,08
S16	Lumbar erector spinae	Left	Lumbar	Convex	Standing	Dynamic stiffness	N/m	198
S16	Lumbar erector spinae	Left	Lumbar	Convex	Prone	Dynamic stiffness	N/m	332
S16	Lumbar erector spinae	Left	Lumbar	Convex	Standing	Logarithmic decrement (elasticity)	dimensionless	1,57
S16	Lumbar erector spinae	Left	Lumbar	Convex	Prone	Logarithmic decrement (elasticity)	dimensionless	1,53
S16	Lumbar erector spinae	Left	Lumbar	Convex	Standing	Mechanical stress relaxation time	ms	20,3
S16	Lumbar erector spinae	Left	Lumbar	Convex	Prone	Mechanical stress relaxation time	ms	15,8
S16	Lumbar erector spinae	Left	Lumbar	Convex	Standing	Creep (Deborah number ratio)	dimensionless	1,17
S16	Lumbar erector spinae	Left	Lumbar	Convex	Prone	Creep (Deborah number ratio)	dimensionless	0,96
S16	Lumbar erector spinae	Right	Lumbar	Concave	Standing	Tone (oscillation frequency)	Hz	11,8
S16	Lumbar erector spinae	Right	Lumbar	Concave	Prone	Tone (oscillation frequency)	Hz	13,9
S16	Lumbar erector spinae	Right	Lumbar	Concave	Standing	Dynamic stiffness	N/m	193
S16	Lumbar erector spinae	Right	Lumbar	Concave	Prone	Dynamic stiffness	N/m	268
S16	Lumbar erector spinae	Right	Lumbar	Concave	Standing	Logarithmic decrement (elasticity)	dimensionless	1,29
S16	Lumbar erector spinae	Right	Lumbar	Concave	Prone	Logarithmic decrement (elasticity)	dimensionless	1,42
S16	Lumbar erector spinae	Right	Lumbar	Concave	Standing	Mechanical stress relaxation time	ms	24,3
S16	Lumbar erector spinae	Right	Lumbar	Concave	Prone	Mechanical stress relaxation time	ms	19,7
S16	Lumbar erector spinae	Right	Lumbar	Concave	Standing	Creep (Deborah number ratio)	dimensionless	1,5
S16	Lumbar erector spinae	Right	Lumbar	Concave	Prone	Creep (Deborah number ratio)	dimensionless	1,22
S17	Middle trapezius	Left	Thoracic	Concave	Standing	Tone (oscillation frequency)	Hz	17,14
S17	Middle trapezius	Left	Thoracic	Concave	Prone	Tone (oscillation frequency)	Hz	14,7
S17	Middle trapezius	Left	Thoracic	Concave	Standing	Dynamic stiffness	N/m	362
S17	Middle trapezius	Left	Thoracic	Concave	Prone	Dynamic stiffness	N/m	250
S17	Middle trapezius	Left	Thoracic	Concave	Standing	Logarithmic decrement (elasticity)	dimensionless	0,64
S17	Middle trapezius	Left	Thoracic	Concave	Prone	Logarithmic decrement (elasticity)	dimensionless	0,8
S17	Middle trapezius	Left	Thoracic	Concave	Standing	Mechanical stress relaxation time	ms	13,8
S17	Middle trapezius	Left	Thoracic	Concave	Prone	Mechanical stress relaxation time	ms	19,1
S17	Middle trapezius	Left	Thoracic	Concave	Standing	Creep (Deborah number ratio)	dimensionless	0,83
S17	Middle trapezius	Left	Thoracic	Concave	Prone	Creep (Deborah number ratio)	dimensionless	1,14
S17	Middle trapezius	Right	Thoracic	Convex	Standing	Tone (oscillation frequency)	Hz	16,74
S17	Middle trapezius	Right	Thoracic	Convex	Prone	Tone (oscillation frequency)	Hz	14,84
S17	Middle trapezius	Right	Thoracic	Convex	Standing	Dynamic stiffness	N/m	343
S17	Middle trapezius	Right	Thoracic	Convex	Prone	Dynamic stiffness	N/m	260
S17	Middle trapezius	Right	Thoracic	Convex	Standing	Logarithmic decrement (elasticity)	dimensionless	0,66
S17	Middle trapezius	Right	Thoracic	Convex	Prone	Logarithmic decrement (elasticity)	dimensionless	0,99
S17	Middle trapezius	Right	Thoracic	Convex	Standing	Mechanical stress relaxation time	ms	14,3
S17	Middle trapezius	Right	Thoracic	Convex	Prone	Mechanical stress relaxation time	ms	18,5
S17	Middle trapezius	Right	Thoracic	Convex	Standing	Creep (Deborah number ratio)	dimensionless	0,87
S17	Middle trapezius	Right	Thoracic	Convex	Prone	Creep (Deborah number ratio)	dimensionless	1,12
S17	Lower trapezius	Left	Thoracic	Concave	Standing	Tone (oscillation frequency)	Hz	13,22
S17	Lower trapezius	Left	Thoracic	Concave	Prone	Tone (oscillation frequency)	Hz	16,88
S17	Lower trapezius	Left	Thoracic	Concave	Standing	Dynamic stiffness	N/m	207
S17	Lower trapezius	Left	Thoracic	Concave	Prone	Dynamic stiffness	N/m	364
S17	Lower trapezius	Left	Thoracic	Concave	Standing	Logarithmic decrement (elasticity)	dimensionless	0,86
S17	Lower trapezius	Left	Thoracic	Concave	Prone	Logarithmic decrement (elasticity)	dimensionless	0,91
S17	Lower trapezius	Left	Thoracic	Concave	Standing	Mechanical stress relaxation time	ms	21,6
S17	Lower trapezius	Left	Thoracic	Concave	Prone	Mechanical stress relaxation time	ms	14,4
S17	Lower trapezius	Left	Thoracic	Concave	Standing	Creep (Deborah number ratio)	dimensionless	1,28
S17	Lower trapezius	Left	Thoracic	Concave	Prone	Creep (Deborah number ratio)	dimensionless	0,87
S17	Lower trapezius	Right	Thoracic	Convex	Standing	Tone (oscillation frequency)	Hz	16,76
S17	Lower trapezius	Right	Thoracic	Convex	Prone	Tone (oscillation frequency)	Hz	15,88
S17	Lower trapezius	Right	Thoracic	Convex	Standing	Dynamic stiffness	N/m	463
S17	Lower trapezius	Right	Thoracic	Convex	Prone	Dynamic stiffness	N/m	300
S17	Lower trapezius	Right	Thoracic	Convex	Standing	Logarithmic decrement (elasticity)	dimensionless	1,11
S17	Lower trapezius	Right	Thoracic	Convex	Prone	Logarithmic decrement (elasticity)	dimensionless	0,94
S17	Lower trapezius	Right	Thoracic	Convex	Standing	Mechanical stress relaxation time	ms	12,7
S17	Lower trapezius	Right	Thoracic	Convex	Prone	Mechanical stress relaxation time	ms	16,4
S17	Lower trapezius	Right	Thoracic	Convex	Standing	Creep (Deborah number ratio)	dimensionless	0,81
S17	Lower trapezius	Right	Thoracic	Convex	Prone	Creep (Deborah number ratio)	dimensionless	0,99
S17	Latissimus dorsi	Left	Thoracic/thoracolumbar	Concave	Standing	Tone (oscillation frequency)	Hz	13,14
S17	Latissimus dorsi	Left	Thoracic/thoracolumbar	Concave	Prone	Tone (oscillation frequency)	Hz	15,12
S17	Latissimus dorsi	Left	Thoracic/thoracolumbar	Concave	Standing	Dynamic stiffness	N/m	267
S17	Latissimus dorsi	Left	Thoracic/thoracolumbar	Concave	Prone	Dynamic stiffness	N/m	352
S17	Latissimus dorsi	Left	Thoracic/thoracolumbar	Concave	Standing	Logarithmic decrement (elasticity)	dimensionless	0,91
S17	Latissimus dorsi	Left	Thoracic/thoracolumbar	Concave	Prone	Logarithmic decrement (elasticity)	dimensionless	1,12
S17	Latissimus dorsi	Left	Thoracic/thoracolumbar	Concave	Standing	Mechanical stress relaxation time	ms	17,3
S17	Latissimus dorsi	Left	Thoracic/thoracolumbar	Concave	Prone	Mechanical stress relaxation time	ms	15,4
S17	Latissimus dorsi	Left	Thoracic/thoracolumbar	Concave	Standing	Creep (Deborah number ratio)	dimensionless	1
S17	Latissimus dorsi	Left	Thoracic/thoracolumbar	Concave	Prone	Creep (Deborah number ratio)	dimensionless	0,93
S17	Latissimus dorsi	Right	Thoracic/thoracolumbar	Convex	Standing	Tone (oscillation frequency)	Hz	12,88
S17	Latissimus dorsi	Right	Thoracic/thoracolumbar	Convex	Prone	Tone (oscillation frequency)	Hz	17,16
S17	Latissimus dorsi	Right	Thoracic/thoracolumbar	Convex	Standing	Dynamic stiffness	N/m	215
S17	Latissimus dorsi	Right	Thoracic/thoracolumbar	Convex	Prone	Dynamic stiffness	N/m	384
S17	Latissimus dorsi	Right	Thoracic/thoracolumbar	Convex	Standing	Logarithmic decrement (elasticity)	dimensionless	0,81
S17	Latissimus dorsi	Right	Thoracic/thoracolumbar	Convex	Prone	Logarithmic decrement (elasticity)	dimensionless	0,78
S17	Latissimus dorsi	Right	Thoracic/thoracolumbar	Convex	Standing	Mechanical stress relaxation time	ms	19,2
S17	Latissimus dorsi	Right	Thoracic/thoracolumbar	Convex	Prone	Mechanical stress relaxation time	ms	13,1
S17	Latissimus dorsi	Right	Thoracic/thoracolumbar	Convex	Standing	Creep (Deborah number ratio)	dimensionless	1,07
S17	Latissimus dorsi	Right	Thoracic/thoracolumbar	Convex	Prone	Creep (Deborah number ratio)	dimensionless	0,79
S17	Lumbar erector spinae	Left	Lumbar	Convex	Standing	Tone (oscillation frequency)	Hz	16,76
S17	Lumbar erector spinae	Left	Lumbar	Convex	Prone	Tone (oscillation frequency)	Hz	13,58
S17	Lumbar erector spinae	Left	Lumbar	Convex	Standing	Dynamic stiffness	N/m	592
S17	Lumbar erector spinae	Left	Lumbar	Convex	Prone	Dynamic stiffness	N/m	246
S17	Lumbar erector spinae	Left	Lumbar	Convex	Standing	Logarithmic decrement (elasticity)	dimensionless	1,07
S17	Lumbar erector spinae	Left	Lumbar	Convex	Prone	Logarithmic decrement (elasticity)	dimensionless	0,72
S17	Lumbar erector spinae	Left	Lumbar	Convex	Standing	Mechanical stress relaxation time	ms	10,8
S17	Lumbar erector spinae	Left	Lumbar	Convex	Prone	Mechanical stress relaxation time	ms	19
S17	Lumbar erector spinae	Left	Lumbar	Convex	Standing	Creep (Deborah number ratio)	dimensionless	0,7

S17	Lumbar erector spinae	Left	Lumbar	Convex	Prone	Creep (Deborah number ratio)	dimensionless	1,08
S17	Lumbar erector spinae	Right	Lumbar	Concave	Standing	Tone (oscillation frequency)	Hz	12,82
S17	Lumbar erector spinae	Right	Lumbar	Concave	Prone	Tone (oscillation frequency)	Hz	12,8
S17	Lumbar erector spinae	Right	Lumbar	Concave	Standing	Dynamic stiffness	N/m	204
S17	Lumbar erector spinae	Right	Lumbar	Concave	Prone	Dynamic stiffness	N/m	163
S17	Lumbar erector spinae	Right	Lumbar	Concave	Standing	Logarithmic decrement (elasticity)	dimensionless	0,75
S17	Lumbar erector spinae	Right	Lumbar	Concave	Prone	Logarithmic decrement (elasticity)	dimensionless	0,51
S17	Lumbar erector spinae	Right	Lumbar	Concave	Standing	Mechanical stress relaxation time	ms	18,5
S17	Lumbar erector spinae	Right	Lumbar	Concave	Prone	Mechanical stress relaxation time	ms	20,9
S17	Lumbar erector spinae	Right	Lumbar	Concave	Standing	Creep (Deborah number ratio)	dimensionless	0,97
S17	Lumbar erector spinae	Right	Lumbar	Concave	Prone	Creep (Deborah number ratio)	dimensionless	1,05
S18	Middle trapezius	Left	Thoracic	Concave	Standing	Tone (oscillation frequency)	Hz	16,18
S18	Middle trapezius	Left	Thoracic	Concave	Prone	Tone (oscillation frequency)	Hz	16,74
S18	Middle trapezius	Left	Thoracic	Concave	Standing	Dynamic stiffness	N/m	325
S18	Middle trapezius	Left	Thoracic	Concave	Prone	Dynamic stiffness	N/m	381
S18	Middle trapezius	Left	Thoracic	Concave	Standing	Logarithmic decrement (elasticity)	dimensionless	0,81
S18	Middle trapezius	Left	Thoracic	Concave	Prone	Logarithmic decrement (elasticity)	dimensionless	1,02
S18	Middle trapezius	Left	Thoracic	Concave	Standing	Mechanical stress relaxation time	ms	14,9
S18	Middle trapezius	Left	Thoracic	Concave	Prone	Mechanical stress relaxation time	ms	13,9
S18	Middle trapezius	Left	Thoracic	Concave	Standing	Creep (Deborah number ratio)	dimensionless	0,88
S18	Middle trapezius	Left	Thoracic	Concave	Prone	Creep (Deborah number ratio)	dimensionless	0,86
S18	Middle trapezius	Right	Thoracic	Convex	Standing	Tone (oscillation frequency)	Hz	18,32
S18	Middle trapezius	Right	Thoracic	Convex	Prone	Tone (oscillation frequency)	Hz	13,9
S18	Middle trapezius	Right	Thoracic	Convex	Standing	Dynamic stiffness	N/m	430
S18	Middle trapezius	Right	Thoracic	Convex	Prone	Dynamic stiffness	N/m	271
S18	Middle trapezius	Right	Thoracic	Convex	Standing	Logarithmic decrement (elasticity)	dimensionless	0,73
S18	Middle trapezius	Right	Thoracic	Convex	Prone	Logarithmic decrement (elasticity)	dimensionless	1,01
S18	Middle trapezius	Right	Thoracic	Convex	Standing	Mechanical stress relaxation time	ms	12,1
S18	Middle trapezius	Right	Thoracic	Convex	Prone	Mechanical stress relaxation time	ms	17,6
S18	Middle trapezius	Right	Thoracic	Convex	Standing	Creep (Deborah number ratio)	dimensionless	0,75
S18	Middle trapezius	Right	Thoracic	Convex	Prone	Creep (Deborah number ratio)	dimensionless	1,05
S18	Lower trapezius	Left	Thoracic	Concave	Standing	Tone (oscillation frequency)	Hz	16,28
S18	Lower trapezius	Left	Thoracic	Concave	Prone	Tone (oscillation frequency)	Hz	15,88
S18	Lower trapezius	Left	Thoracic	Concave	Standing	Dynamic stiffness	N/m	350
S18	Lower trapezius	Left	Thoracic	Concave	Prone	Dynamic stiffness	N/m	333
S18	Lower trapezius	Left	Thoracic	Concave	Standing	Logarithmic decrement (elasticity)	dimensionless	1,01
S18	Lower trapezius	Left	Thoracic	Concave	Prone	Logarithmic decrement (elasticity)	dimensionless	0,87
S18	Lower trapezius	Left	Thoracic	Concave	Standing	Mechanical stress relaxation time	ms	14,8
S18	Lower trapezius	Left	Thoracic	Concave	Prone	Mechanical stress relaxation time	ms	15,1
S18	Lower trapezius	Left	Thoracic	Concave	Standing	Creep (Deborah number ratio)	dimensionless	0,9
S18	Lower trapezius	Left	Thoracic	Concave	Prone	Creep (Deborah number ratio)	dimensionless	0,91
S18	Lower trapezius	Right	Thoracic	Convex	Standing	Tone (oscillation frequency)	Hz	15,02
S18	Lower trapezius	Right	Thoracic	Convex	Prone	Tone (oscillation frequency)	Hz	14
S18	Lower trapezius	Right	Thoracic	Convex	Standing	Dynamic stiffness	N/m	280
S18	Lower trapezius	Right	Thoracic	Convex	Prone	Dynamic stiffness	N/m	341
S18	Lower trapezius	Right	Thoracic	Convex	Standing	Logarithmic decrement (elasticity)	dimensionless	0,93
S18	Lower trapezius	Right	Thoracic	Convex	Prone	Logarithmic decrement (elasticity)	dimensionless	1,14
S18	Lower trapezius	Right	Thoracic	Convex	Standing	Mechanical stress relaxation time	ms	16,8
S18	Lower trapezius	Right	Thoracic	Convex	Prone	Mechanical stress relaxation time	ms	15,5
S18	Lower trapezius	Right	Thoracic	Convex	Standing	Creep (Deborah number ratio)	dimensionless	0,99
S18	Lower trapezius	Right	Thoracic	Convex	Prone	Creep (Deborah number ratio)	dimensionless	0,93
S18	Latissimus dorsi	Left	Thoracic/thoracolumbar	Concave	Standing	Tone (oscillation frequency)	Hz	14,64
S18	Latissimus dorsi	Left	Thoracic/thoracolumbar	Concave	Prone	Tone (oscillation frequency)	Hz	10,86
S18	Latissimus dorsi	Left	Thoracic/thoracolumbar	Concave	Standing	Dynamic stiffness	N/m	302
S18	Latissimus dorsi	Left	Thoracic/thoracolumbar	Concave	Prone	Dynamic stiffness	N/m	124
S18	Latissimus dorsi	Left	Thoracic/thoracolumbar	Concave	Standing	Logarithmic decrement (elasticity)	dimensionless	1,1
S18	Latissimus dorsi	Left	Thoracic/thoracolumbar	Concave	Prone	Logarithmic decrement (elasticity)	dimensionless	0,59
S18	Latissimus dorsi	Left	Thoracic/thoracolumbar	Concave	Standing	Mechanical stress relaxation time	ms	16
S18	Latissimus dorsi	Left	Thoracic/thoracolumbar	Concave	Prone	Mechanical stress relaxation time	ms	23,9
S18	Latissimus dorsi	Left	Thoracic/thoracolumbar	Concave	Standing	Creep (Deborah number ratio)	dimensionless	0,96
S18	Latissimus dorsi	Left	Thoracic/thoracolumbar	Concave	Prone	Creep (Deborah number ratio)	dimensionless	1,13
S18	Latissimus dorsi	Right	Thoracic/thoracolumbar	Convex	Standing	Tone (oscillation frequency)	Hz	14,42
S18	Latissimus dorsi	Right	Thoracic/thoracolumbar	Convex	Prone	Tone (oscillation frequency)	Hz	11,02
S18	Latissimus dorsi	Right	Thoracic/thoracolumbar	Convex	Standing	Dynamic stiffness	N/m	305
S18	Latissimus dorsi	Right	Thoracic/thoracolumbar	Convex	Prone	Dynamic stiffness	N/m	125
S18	Latissimus dorsi	Right	Thoracic/thoracolumbar	Convex	Standing	Logarithmic decrement (elasticity)	dimensionless	1,03
S18	Latissimus dorsi	Right	Thoracic/thoracolumbar	Convex	Prone	Logarithmic decrement (elasticity)	dimensionless	0,61
S18	Latissimus dorsi	Right	Thoracic/thoracolumbar	Convex	Standing	Mechanical stress relaxation time	ms	15,9
S18	Latissimus dorsi	Right	Thoracic/thoracolumbar	Convex	Prone	Mechanical stress relaxation time	ms	25,1
S18	Latissimus dorsi	Right	Thoracic/thoracolumbar	Convex	Standing	Creep (Deborah number ratio)	dimensionless	0,94
S18	Latissimus dorsi	Right	Thoracic/thoracolumbar	Convex	Prone	Creep (Deborah number ratio)	dimensionless	1,27
S18	Lumbar erector spinae	Left	Lumbar	Convex	Standing	Tone (oscillation frequency)	Hz	11,18
S18	Lumbar erector spinae	Left	Lumbar	Convex	Prone	Tone (oscillation frequency)	Hz	10,9
S18	Lumbar erector spinae	Left	Lumbar	Convex	Standing	Dynamic stiffness	N/m	142
S18	Lumbar erector spinae	Left	Lumbar	Convex	Prone	Dynamic stiffness	N/m	119
S18	Lumbar erector spinae	Left	Lumbar	Convex	Standing	Logarithmic decrement (elasticity)	dimensionless	0,58
S18	Lumbar erector spinae	Left	Lumbar	Convex	Prone	Logarithmic decrement (elasticity)	dimensionless	0,66
S18	Lumbar erector spinae	Left	Lumbar	Convex	Standing	Mechanical stress relaxation time	ms	22,1
S18	Lumbar erector spinae	Left	Lumbar	Convex	Prone	Mechanical stress relaxation time	ms	24,5
S18	Lumbar erector spinae	Left	Lumbar	Convex	Standing	Creep (Deborah number ratio)	dimensionless	1,06
S18	Lumbar erector spinae	Left	Lumbar	Convex	Prone	Creep (Deborah number ratio)	dimensionless	1,13
S18	Lumbar erector spinae	Right	Lumbar	Concave	Standing	Tone (oscillation frequency)	Hz	10,68
S18	Lumbar erector spinae	Right	Lumbar	Concave	Prone	Tone (oscillation frequency)	Hz	10,84
S18	Lumbar erector spinae	Right	Lumbar	Concave	Standing	Dynamic stiffness	N/m	117
S18	Lumbar erector spinae	Right	Lumbar	Concave	Prone	Dynamic stiffness	N/m	120
S18	Lumbar erector spinae	Right	Lumbar	Concave	Standing	Logarithmic decrement (elasticity)	dimensionless	0,72
S18	Lumbar erector spinae	Right	Lumbar	Concave	Prone	Logarithmic decrement (elasticity)	dimensionless	0,61
S18	Lumbar erector spinae	Right	Lumbar	Concave	Standing	Mechanical stress relaxation time	ms	24,2
S18	Lumbar erector spinae	Right	Lumbar	Concave	Prone	Mechanical stress relaxation time	ms	24,7
S18	Lumbar erector spinae	Right	Lumbar	Concave	Standing	Creep (Deborah number ratio)	dimensionless	1,12

S18	Lumbar erector spinae	Right	Lumbar	Concave	Prone	Creep (Deborah number ratio)	dimensionless	1,2
S19	Middle trapezius	Left	Thoracic	Concave	Standing	Tone (oscillation frequency)	Hz	14,44
S19	Middle trapezius	Left	Thoracic	Concave	Prone	Tone (oscillation frequency)	Hz	12,1
S19	Middle trapezius	Left	Thoracic	Concave	Standing	Dynamic stiffness	N/m	263
S19	Middle trapezius	Left	Thoracic	Concave	Prone	Dynamic stiffness	N/m	180
S19	Middle trapezius	Left	Thoracic	Concave	Standing	Logarithmic decrement (elasticity)	dimensionless	1,02
S19	Middle trapezius	Left	Thoracic	Concave	Prone	Logarithmic decrement (elasticity)	dimensionless	1,14
S19	Middle trapezius	Left	Thoracic	Concave	Standing	Mechanical stress relaxation time	ms	19,1
S19	Middle trapezius	Left	Thoracic	Concave	Prone	Mechanical stress relaxation time	ms	23,5
S19	Middle trapezius	Left	Thoracic	Concave	Standing	Creep (Deborah number ratio)	dimensionless	1,16
S19	Middle trapezius	Left	Thoracic	Concave	Prone	Creep (Deborah number ratio)	dimensionless	1,4
S19	Middle trapezius	Right	Thoracic	Convex	Standing	Tone (oscillation frequency)	Hz	15,52
S19	Middle trapezius	Right	Thoracic	Convex	Prone	Tone (oscillation frequency)	Hz	12,7
S19	Middle trapezius	Right	Thoracic	Convex	Standing	Dynamic stiffness	N/m	286
S19	Middle trapezius	Right	Thoracic	Convex	Prone	Dynamic stiffness	N/m	235
S19	Middle trapezius	Right	Thoracic	Convex	Standing	Logarithmic decrement (elasticity)	dimensionless	1,03
S19	Middle trapezius	Right	Thoracic	Convex	Prone	Logarithmic decrement (elasticity)	dimensionless	1,26
S19	Middle trapezius	Right	Thoracic	Convex	Standing	Mechanical stress relaxation time	ms	18,2
S19	Middle trapezius	Right	Thoracic	Convex	Prone	Mechanical stress relaxation time	ms	19,7
S19	Middle trapezius	Right	Thoracic	Convex	Standing	Creep (Deborah number ratio)	dimensionless	1,11
S19	Middle trapezius	Right	Thoracic	Convex	Prone	Creep (Deborah number ratio)	dimensionless	1,17
S19	Lower trapezius	Left	Thoracic	Concave	Standing	Tone (oscillation frequency)	Hz	15,08
S19	Lower trapezius	Left	Thoracic	Concave	Prone	Tone (oscillation frequency)	Hz	17,18
S19	Lower trapezius	Left	Thoracic	Concave	Standing	Dynamic stiffness	N/m	320
S19	Lower trapezius	Left	Thoracic	Concave	Prone	Dynamic stiffness	N/m	466
S19	Lower trapezius	Left	Thoracic	Concave	Standing	Logarithmic decrement (elasticity)	dimensionless	1,2
S19	Lower trapezius	Left	Thoracic	Concave	Prone	Logarithmic decrement (elasticity)	dimensionless	1,4
S19	Lower trapezius	Left	Thoracic	Concave	Standing	Mechanical stress relaxation time	ms	15,9
S19	Lower trapezius	Left	Thoracic	Concave	Prone	Mechanical stress relaxation time	ms	12,5
S19	Lower trapezius	Left	Thoracic	Concave	Standing	Creep (Deborah number ratio)	dimensionless	0,98
S19	Lower trapezius	Left	Thoracic	Concave	Prone	Creep (Deborah number ratio)	dimensionless	0,79
S19	Lower trapezius	Right	Thoracic	Convex	Standing	Tone (oscillation frequency)	Hz	17,24
S19	Lower trapezius	Right	Thoracic	Convex	Prone	Tone (oscillation frequency)	Hz	14,52
S19	Lower trapezius	Right	Thoracic	Convex	Standing	Dynamic stiffness	N/m	395
S19	Lower trapezius	Right	Thoracic	Convex	Prone	Dynamic stiffness	N/m	293
S19	Lower trapezius	Right	Thoracic	Convex	Standing	Logarithmic decrement (elasticity)	dimensionless	1,05
S19	Lower trapezius	Right	Thoracic	Convex	Prone	Logarithmic decrement (elasticity)	dimensionless	1,22
S19	Lower trapezius	Right	Thoracic	Convex	Standing	Mechanical stress relaxation time	ms	13,5
S19	Lower trapezius	Right	Thoracic	Convex	Prone	Mechanical stress relaxation time	ms	17,2
S19	Lower trapezius	Right	Thoracic	Convex	Standing	Creep (Deborah number ratio)	dimensionless	0,85
S19	Lower trapezius	Right	Thoracic	Convex	Prone	Creep (Deborah number ratio)	dimensionless	1,04
S19	Latissimus dorsi	Left	Thoracic/thoracolumbar	Concave	Standing	Tone (oscillation frequency)	Hz	12,36
S19	Latissimus dorsi	Left	Thoracic/thoracolumbar	Concave	Prone	Tone (oscillation frequency)	Hz	12,8
S19	Latissimus dorsi	Left	Thoracic/thoracolumbar	Concave	Standing	Dynamic stiffness	N/m	230
S19	Latissimus dorsi	Left	Thoracic/thoracolumbar	Concave	Prone	Dynamic stiffness	N/m	240
S19	Latissimus dorsi	Left	Thoracic/thoracolumbar	Concave	Standing	Logarithmic decrement (elasticity)	dimensionless	1,08
S19	Latissimus dorsi	Left	Thoracic/thoracolumbar	Concave	Prone	Logarithmic decrement (elasticity)	dimensionless	1,1
S19	Latissimus dorsi	Left	Thoracic/thoracolumbar	Concave	Standing	Mechanical stress relaxation time	ms	19,1
S19	Latissimus dorsi	Left	Thoracic/thoracolumbar	Concave	Prone	Mechanical stress relaxation time	ms	20,2
S19	Latissimus dorsi	Left	Thoracic/thoracolumbar	Concave	Standing	Creep (Deborah number ratio)	dimensionless	1,08
S19	Latissimus dorsi	Left	Thoracic/thoracolumbar	Concave	Prone	Creep (Deborah number ratio)	dimensionless	1,21
S19	Latissimus dorsi	Right	Thoracic/thoracolumbar	Convex	Standing	Tone (oscillation frequency)	Hz	12,3
S19	Latissimus dorsi	Right	Thoracic/thoracolumbar	Convex	Prone	Tone (oscillation frequency)	Hz	12,84
S19	Latissimus dorsi	Right	Thoracic/thoracolumbar	Convex	Standing	Dynamic stiffness	N/m	261
S19	Latissimus dorsi	Right	Thoracic/thoracolumbar	Convex	Prone	Dynamic stiffness	N/m	237
S19	Latissimus dorsi	Right	Thoracic/thoracolumbar	Convex	Standing	Logarithmic decrement (elasticity)	dimensionless	1,17
S19	Latissimus dorsi	Right	Thoracic/thoracolumbar	Convex	Prone	Logarithmic decrement (elasticity)	dimensionless	1,14
S19	Latissimus dorsi	Right	Thoracic/thoracolumbar	Convex	Standing	Mechanical stress relaxation time	ms	19,3
S19	Latissimus dorsi	Right	Thoracic/thoracolumbar	Convex	Prone	Mechanical stress relaxation time	ms	19,7
S19	Latissimus dorsi	Right	Thoracic/thoracolumbar	Convex	Standing	Creep (Deborah number ratio)	dimensionless	1,13
S19	Latissimus dorsi	Right	Thoracic/thoracolumbar	Convex	Prone	Creep (Deborah number ratio)	dimensionless	1,15
S19	Lumbar erector spinae	Left	Lumbar	Convex	Standing	Tone (oscillation frequency)	Hz	10,6
S19	Lumbar erector spinae	Left	Lumbar	Convex	Prone	Tone (oscillation frequency)	Hz	11
S19	Lumbar erector spinae	Left	Lumbar	Convex	Standing	Dynamic stiffness	N/m	132
S19	Lumbar erector spinae	Left	Lumbar	Convex	Prone	Dynamic stiffness	N/m	131
S19	Lumbar erector spinae	Left	Lumbar	Convex	Standing	Logarithmic decrement (elasticity)	dimensionless	0,66
S19	Lumbar erector spinae	Left	Lumbar	Convex	Prone	Logarithmic decrement (elasticity)	dimensionless	0,68
S19	Lumbar erector spinae	Left	Lumbar	Convex	Standing	Mechanical stress relaxation time	ms	22,9
S19	Lumbar erector spinae	Left	Lumbar	Convex	Prone	Mechanical stress relaxation time	ms	25,6
S19	Lumbar erector spinae	Left	Lumbar	Convex	Standing	Creep (Deborah number ratio)	dimensionless	1,12
S19	Lumbar erector spinae	Left	Lumbar	Convex	Prone	Creep (Deborah number ratio)	dimensionless	1,34
S19	Lumbar erector spinae	Right	Lumbar	Concave	Standing	Tone (oscillation frequency)	Hz	10,54
S19	Lumbar erector spinae	Right	Lumbar	Concave	Prone	Tone (oscillation frequency)	Hz	11,06
S19	Lumbar erector spinae	Right	Lumbar	Concave	Standing	Dynamic stiffness	N/m	116
S19	Lumbar erector spinae	Right	Lumbar	Concave	Prone	Dynamic stiffness	N/m	128
S19	Lumbar erector spinae	Right	Lumbar	Concave	Standing	Logarithmic decrement (elasticity)	dimensionless	0,73
S19	Lumbar erector spinae	Right	Lumbar	Concave	Prone	Logarithmic decrement (elasticity)	dimensionless	0,84
S19	Lumbar erector spinae	Right	Lumbar	Concave	Standing	Mechanical stress relaxation time	ms	25,3
S19	Lumbar erector spinae	Right	Lumbar	Concave	Prone	Mechanical stress relaxation time	ms	26,3
S19	Lumbar erector spinae	Right	Lumbar	Concave	Standing	Creep (Deborah number ratio)	dimensionless	1,19
S19	Lumbar erector spinae	Right	Lumbar	Concave	Prone	Creep (Deborah number ratio)	dimensionless	1,42
